# Supplementary material for: A reproducible and physiologically relevant human iPSC-derived platform for in vitro modeling of the neurocardiac junction
Source: Mol Ther. 2026 Apr 1;34(8):4921–41. doi: 10.1016/j.ymthe.2026.03.034 (PMC13464303; doi:10.1016/j.ymthe.2026.03.034)
Supplement: Supplementary file 1 [file mmc1.pdf]

YMTHE, Volume *34*

Supplemental Information

A reproducible and physiologically relevant human iPSC-derived platform for *in vitro* modeling of the neurocardiac junction

Giada Cattelan, Claudia Altomare, Giovanna Gentile, Alexandros A. Lavdas, Laura Sophie Frommelt, Chiara Volani, Luisa Foco, Pietro Girardi, Elisa Peducci, Amparo Guerrero Gerboles, Michele Miragoli, Luisa Petti, Peter P. Pramstaller, Lucio Barile, Serena Zacchigna, Irene Pichler, Alessandra Rossini, Alessandra Zanon, and Marzia De Bortoli

SUPPLEMENTAL FILES

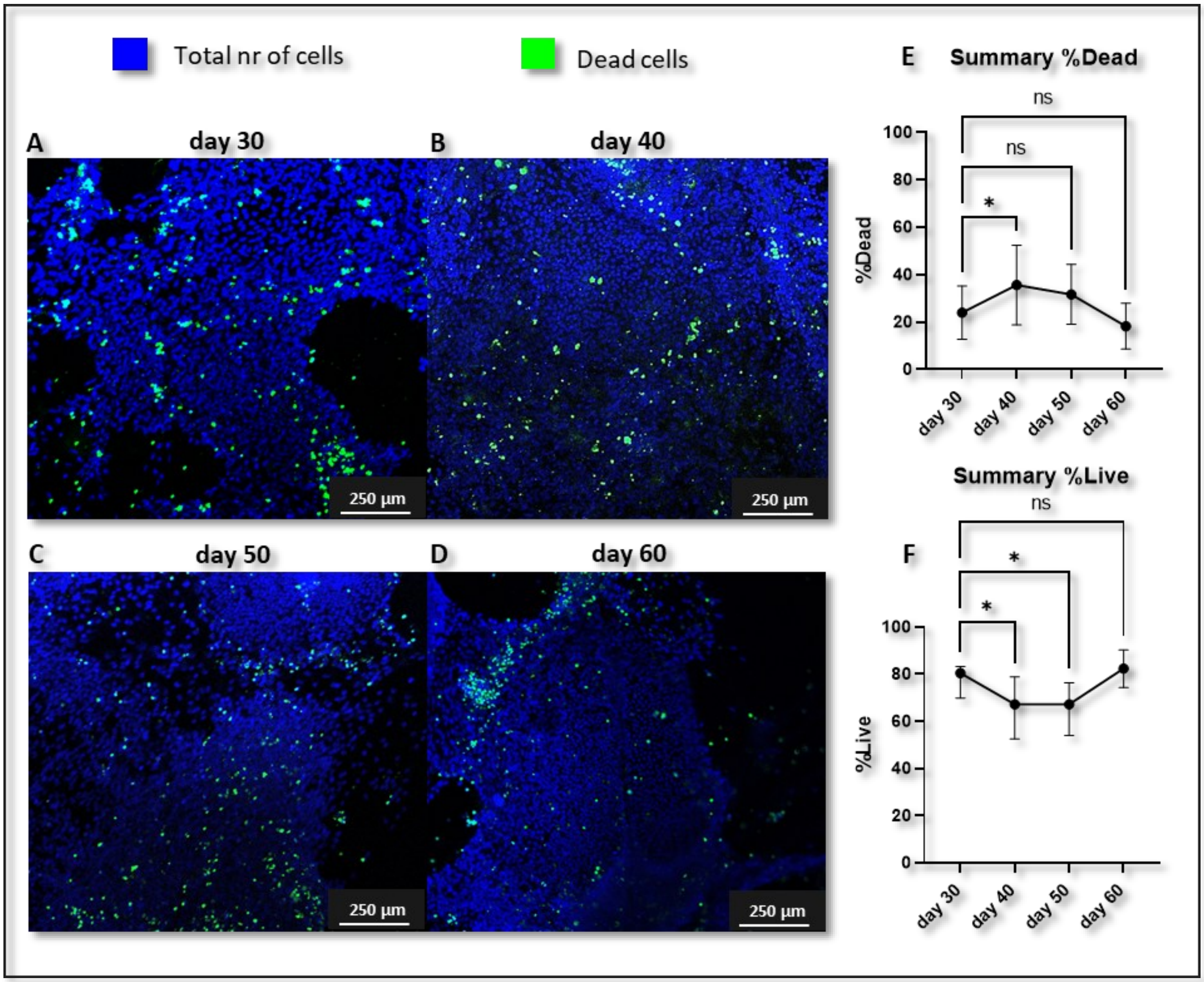

Figure S1: Live and Dead immunostaining of hiPSC-SNs at different timepoints does not show an excessive cell death during the prolonged time in culture.

A-D: Representative images of hiPSC-SN at day 30, day 40, day 50 and day 60 stained in live with the Live and Dead assay. Blue= NucBlue (total number of cells). Green= NucGreen (dead cells). Live cells were obtained subtracting to the total nr. of cells the amount of dead cells. Scale bar 250 µm.

E: Quantitative analysis of the percentage of Dead Cells. IQR with median of 3 differentiations. Test used Kruskal-Wallis: day 30 vs. day 40 p= 0.028

F: Quantitative analysis of the percentage of Live Cells. IQR with median of 3 differentiations. Test used Kruskal-Wallis: day 30 vs. day 40 p= 0.034; day 30 vs. day 50 p= 0.048

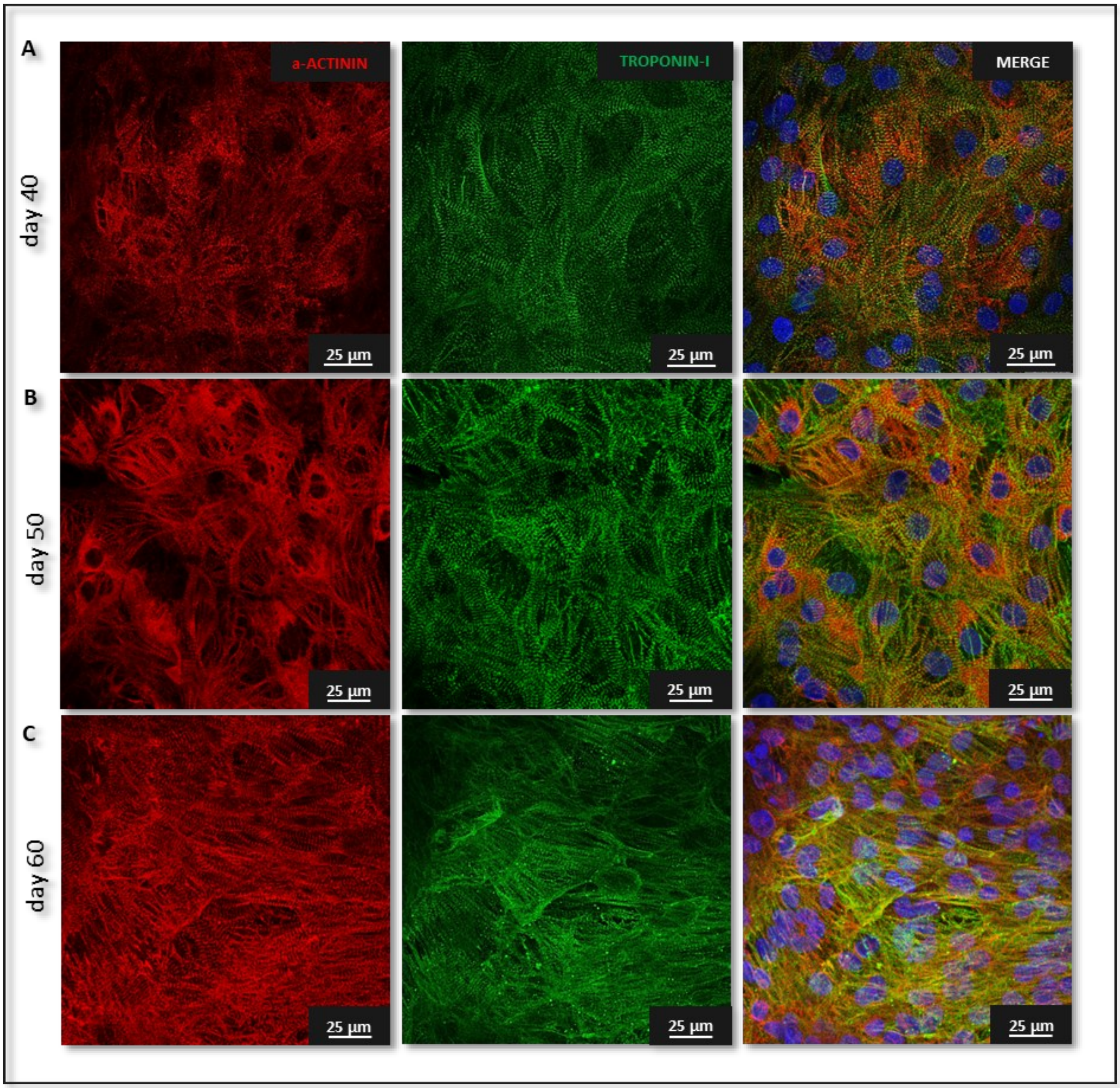

Figure S2: Representative images of hiPSC-CMs at different timepoints stained with sarcomeric markers.

A: hiPSC-CMs at day 40, B: hiPSC-CMs at day 50, C: hiPSC-CMs at day 60. α-actinin is stained in red, troponin-I in green and nuclei with DAPI. Scale bar 25 μm.

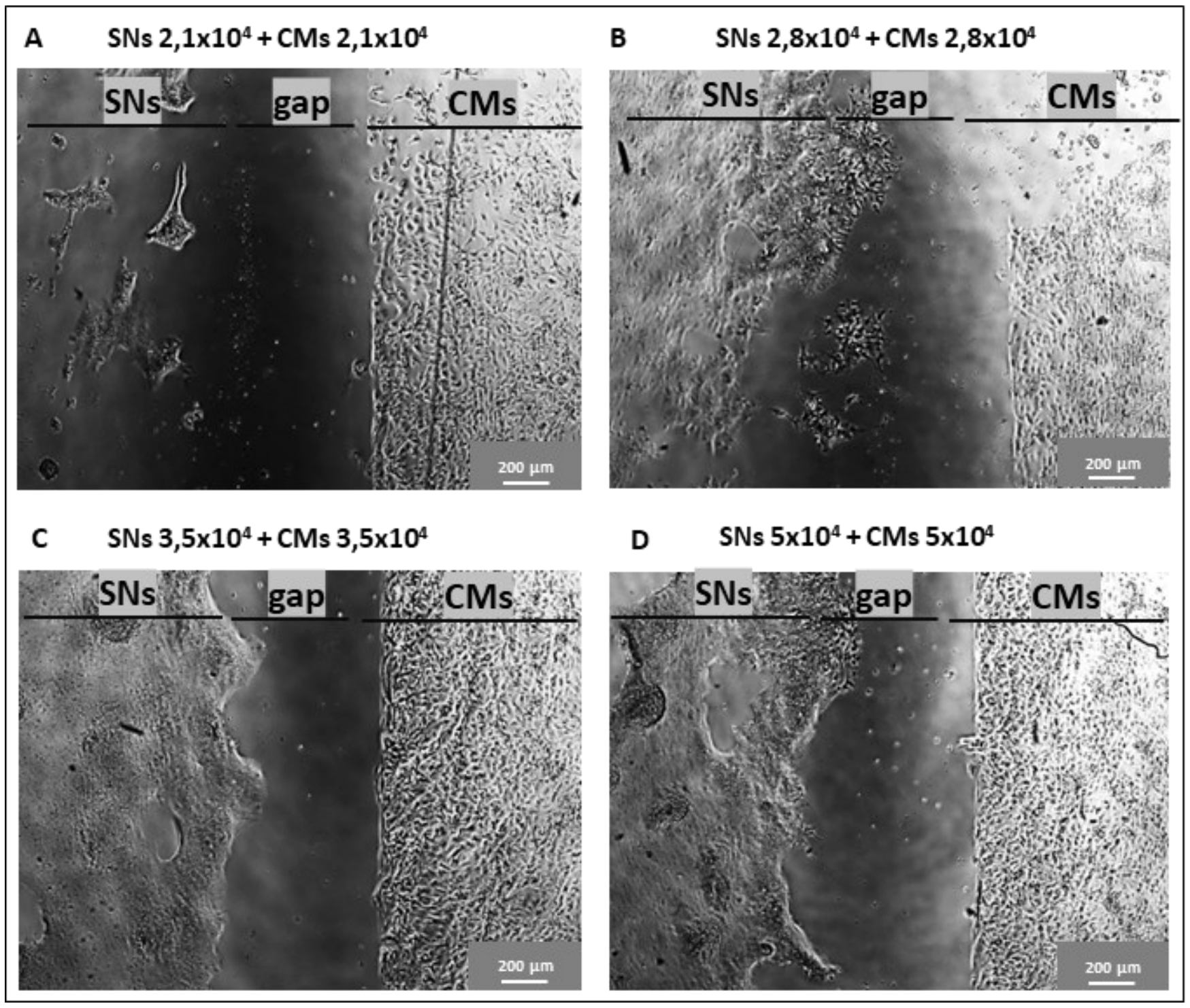

Figure S3: Different cell densities plated in the insert.

A: hiPSC-derived SNs and hiPSC-derived CMs plated at 2,1x10^4 cell/chamber. Scale bar 200 µm.

B: hiPSC-derived SNs and iPSC-derived CMs plated at 2,8x10^4 cell/chamber. Scale bar 200 µm.

C: hiPSC-derived SNs and iPSC-derived CMs plated at 3.5x10^4 cell/chamber. Scale bar 200 µm.

D: hiPSC-derived SNs and iPSC-derived CMs plated at 5x10^4 cell/chamber. Scale bar 200 µm.

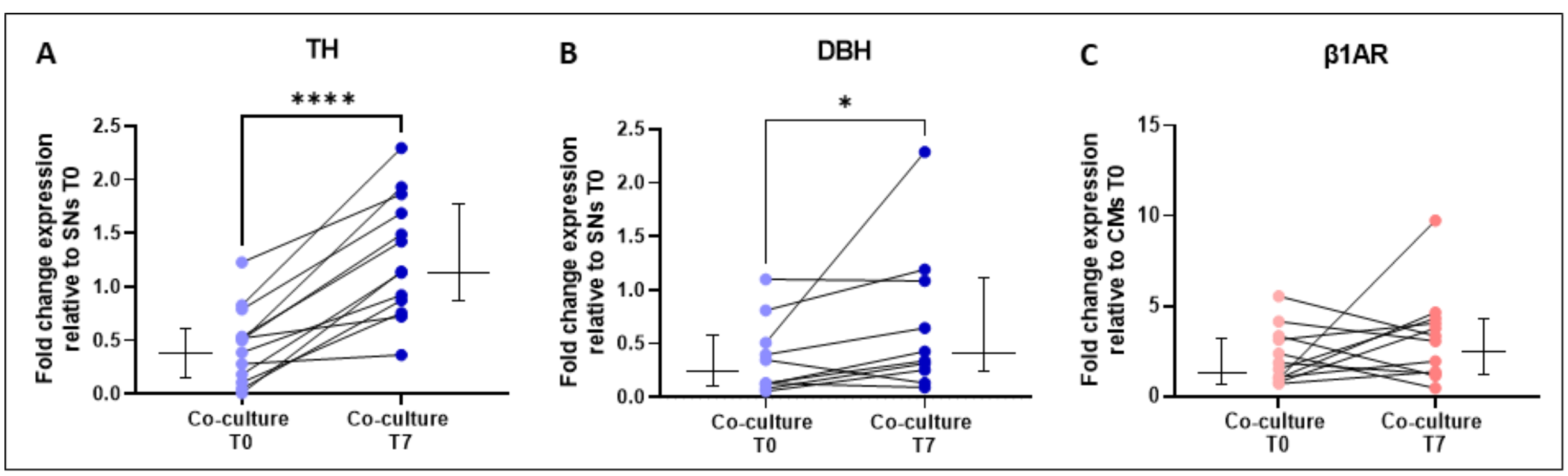

Figure S4: *TH, DBH and β1AR* gene expression analysis after 7 days of co-cultures.

A-C: qRT-PCR of co-cultures at timepoint T0 and timepoint T7.

A: *TH* expression of co-cultures at timepoint T0 vs. timepoint T7. Median with IQR of 13 differentiations with 1 replicate. Wilcoxon test; p value: co-cultures at timepoint T0 vs. timepoint T7 = 0.0002

B: *DBH* expression of co-cultures at timepoint T0 vs. timepoint T7. Median with IQR of 10 differentiations with 1 replicate. Wilcoxon test; p value: co-cultures at timepoint T0 vs. timepoint T7 =0.048

C: *β1AR* expression of co-cultures at timepoint T0 vs. timepoint T7. Median with IQR of 12 differentiations with 1 replicate. Wilcoxon test; p value: co-cultures at timepoint T0 vs. timepoint T7 = Not significant.

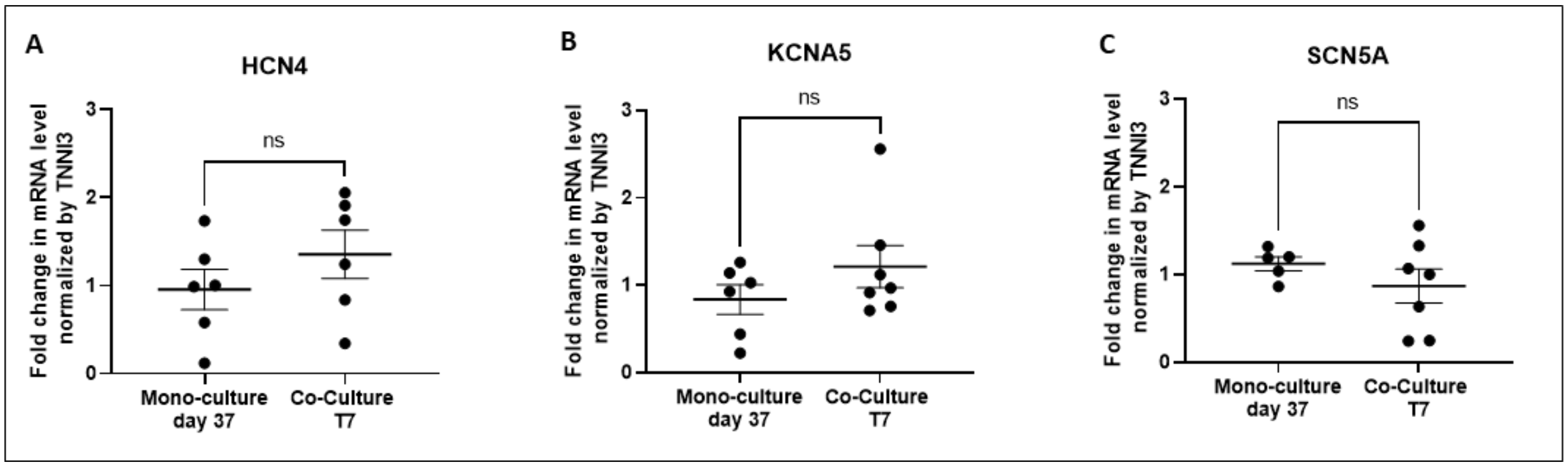

Figure S5: Ion channel genes expression analysis in mono and co-culture hiPSC-CMs.

A-C: qRT-PCR of mono-cultures day 37 and co-cultures T7, corresponding to the same differentiation age.
A: *HCN4* expression of mono-cultures day 37 vs. co-cultures T7. Mean with SEM of 5-7 differentiations with 1 replicate. Unpaired t-test; p value: mono-cultures day 37 and co-cultures T7 = Not significant.
B: *KCNA5* expression of mono-cultures day 37 vs. co-cultures T7. Median with IQR of 5-7 differentiations with 1 replicate. Mann-Whitney test; p value: mono-cultures day 37 and co-cultures T7 = Not significant.
C: *SCN5A* expression of mono-cultures day 37 vs. co-cultures T7. Mean with SEM of 5-7 differentiations with 1 replicate. Unpaired t-test; p value: mono-cultures day 37 and co-cultures T7 = Not significant.

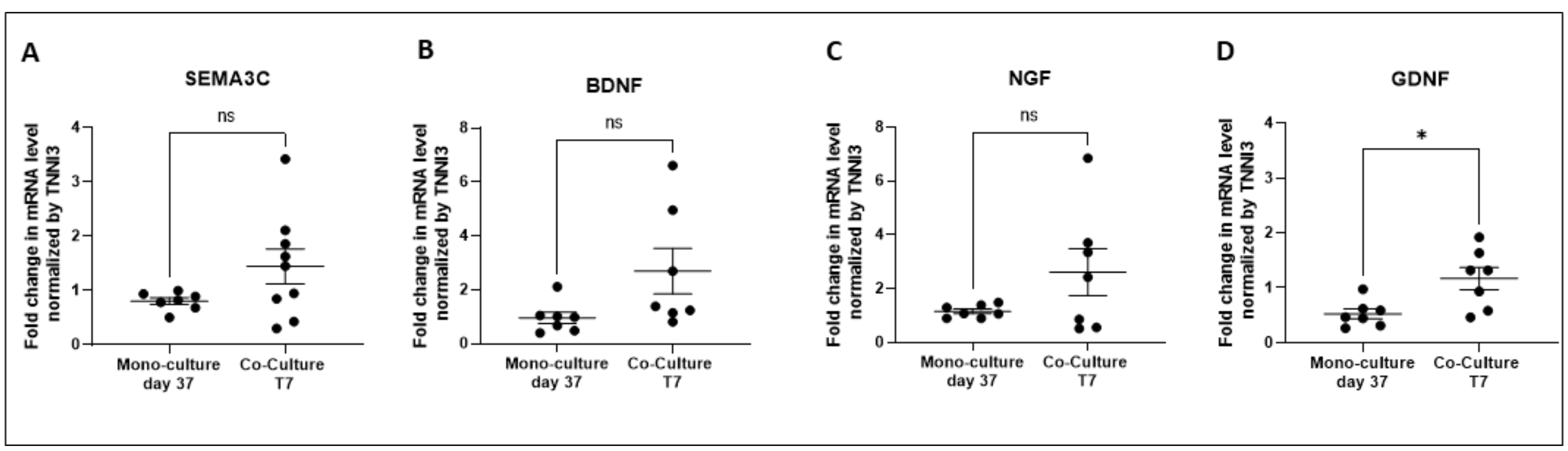

Figure S6: Axon guidance genes expression analysis in mono and co-culture hiPSC-CMs.

A-C: qRT-PCR of mono-culture day 37 and co-cultures T7, corresponding to the same differentiation age
A: *SEMA3C* expression of mono-cultures day 37 vs. co-cultures T7. Mean with SEM of 7-9 differentiations with 1 replicate. Unpaired t-test; p value: mono-cultures day 37 and co-cultures T7 = Not significant.
B: *BDNF* expression of mono-cultures day 37 vs. co-cultures T7. Mean with SEM of 7-9 differentiations with 1 replicate. Unpaired t-test; p value: mono-cultures day 37 and co-cultures T7=Not significant.
C: *NGF* expression of mono-cultures day 37 vs. co-cultures T7. Mean with SEM of 7-9 differentiations with 1 replicate. Unpaired t-test; p value: mono-cultures day 37 and co-cultures T7 = Not significant.
D: *GDNF* expression of mono-cultures day 37 vs. co-cultures T7. Mean with SEM of 7-9 differentiations with 1 replicate. Unpaired t-test; p value: mono-cultures day 37 and co-cultures T7 = 0.013.

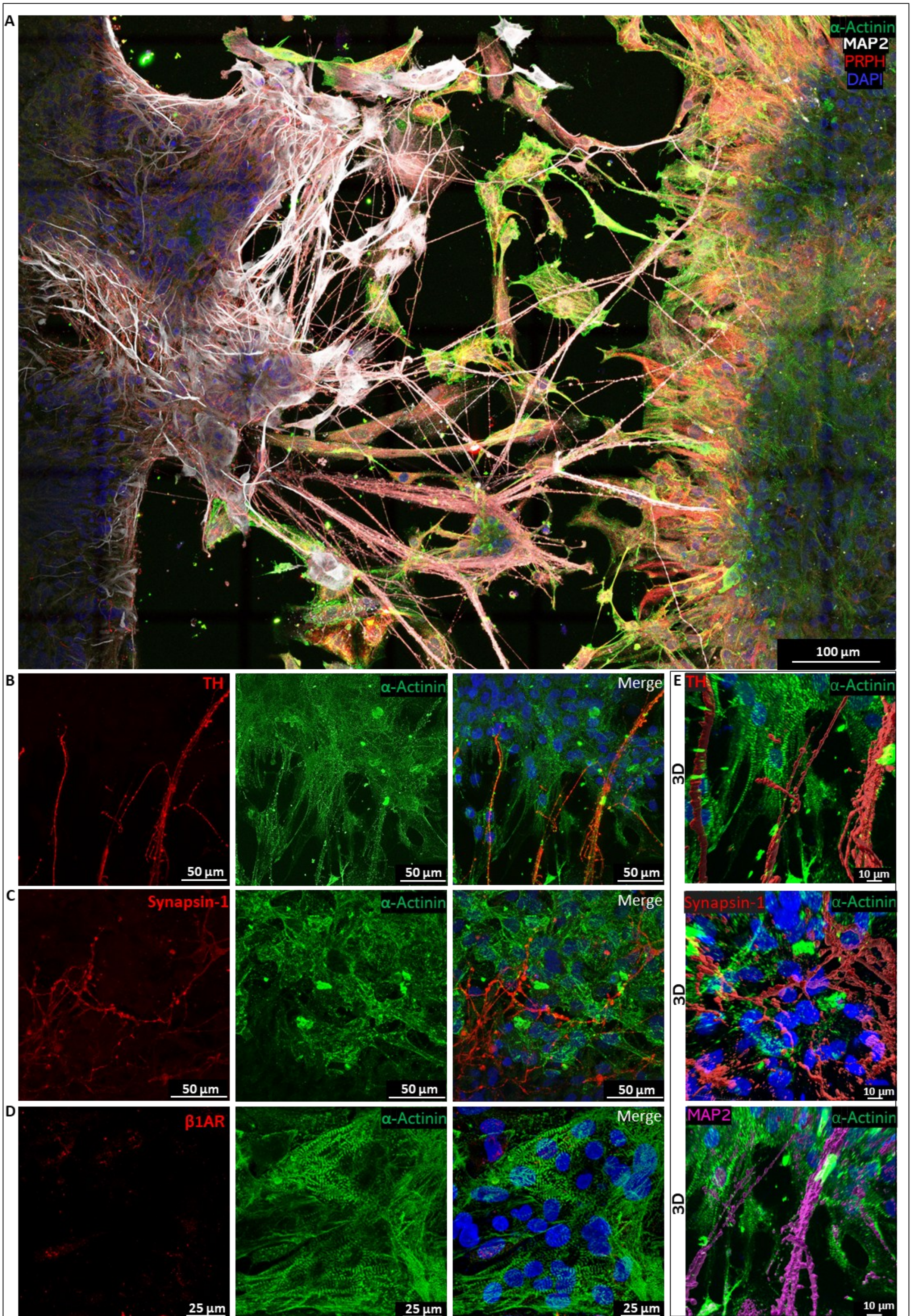

A
α-Actinin
MAP2
PRPH
DAPI
100 µm
B
TH
50 µm
α-Actinin
50 µm
Merge
50 µm
C
Synapsin-1
50 µm
α-Actinin
50 µm
Merge
50 µm
D
β1AR
25 µm
α-Actinin
25 µm
Merge
25 µm
E
TH
α-Actinin
3D
10 µm
Synapsin-1
α-Actinin
3D
10 µm
MAP2
α-Actinin
3D
10 µm

Figure S7: Immunocytochemical images of CTR co-cultures.

A: Reconstruction of a large area of a neurocardiac co-culture. Immunostaining experiment showing neuronal axons in red (PRPH) and white (MAP2), and cardiomyocytes in green (sarcomeric α-actinin). Nuclei were stained with DAPI (blue). Scale bar 100 µm.

B-D: Qualitative immunostaining images of co-culture models at timepoint T7.

B: CTR hiPSC-SNs stained with TH (red) and CTR hiPSC-CMs stained with α-actinin (green). Scale bar 50 µm.

C: CTR hiPSC-SNs stained with Synapsin-1 (red) and CTR hiPSC-CMs stained with α-actinin (green). Scale bar 50 µm.

D: CTR hiPSC-CMs stained with β1AR (red) and α-actinin (green). Scale bar 50 µm.

E: 3D reconstruction of the co-cultures. Scale bar 10 µm. Nuclei were counterstained with DAPI (blu).

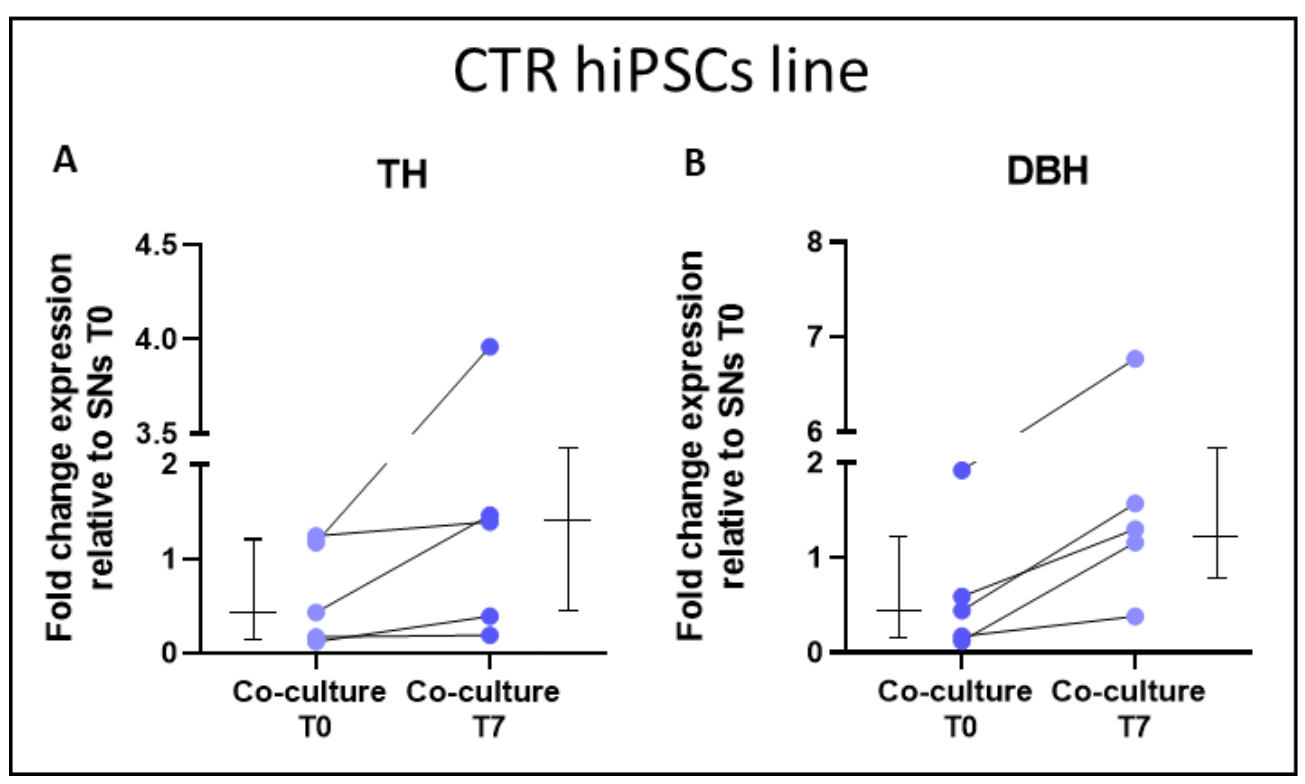

Figure S8: *TH and DBH* gene expression analysis in CTR hiPSCs Line after 7 days of co-cultures.

A-B: qRT-PCR of CTR co-cultures at timepoint T0 and timepoint T7.

A: *TH* expression of co-cultures at timepoint T0 vs. timepoint T7. Median with IQR of 5 differentiations with 1 replicate. Paired-t test; p value: co-cultures at timepoint T0 vs. timepoint T7 = Not significant.

B: *DBH* expression of co-cultures at timepoint T0 vs. timepoint T7. Median with IQR of 5 differentiations with 1 replicate. Wilcoxon test; p value: co-cultures at timepoint T0 vs. timepoint T7 = Not significant.

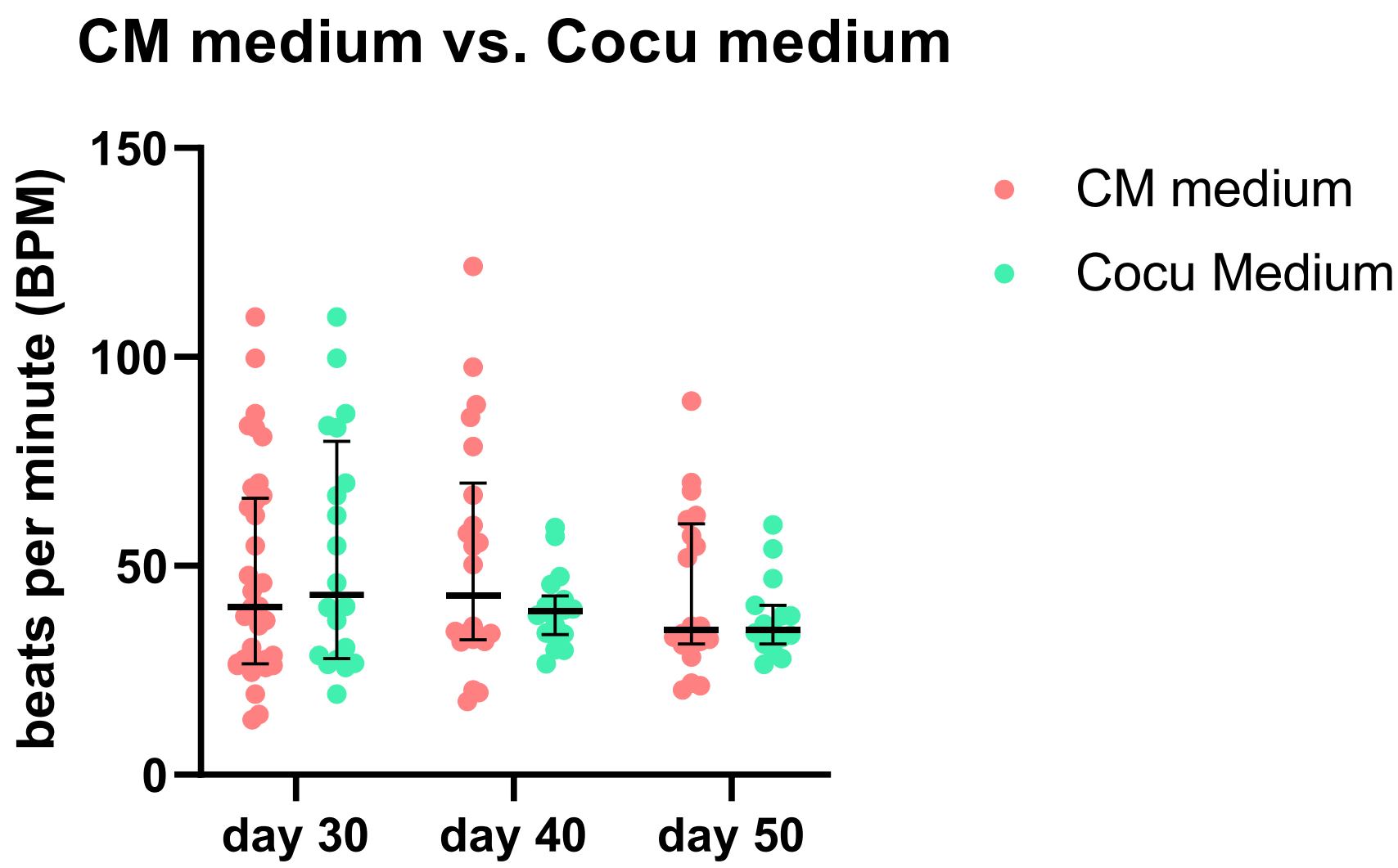

Figure S9: Comparison of the beating rate of monocultured hiPSC-CMs cultivated in standard basal medium or with conditioned co-culture medium.

Comparison of the beating rate of hiPSC-CMs cultured with two different media. The same batch of cells was cultivated with basal medium (RPMI + B27) or co-culture medium (1:1 basal medium of hiPSC-CMs and basal medium hiPSC-SNs). We observed no evidence of a significant difference in the number of beats when moving from the basal to the co-culture medium group (beta -4.96, 95% confidence interval -10.92; 1.00, p=0.103) and no evidence of a difference over time (beta -2.26, 95% CI -6,94; 2.42, p=0.345). Data were analyzed using a multilevel linear random intercept and random slope model (linear mixed effects model), to simultaneously account for both the hierarchical structure of the data (independent experiments) and their longitudinal nature (number of beats observed at different time points in the same cells for each experiment).

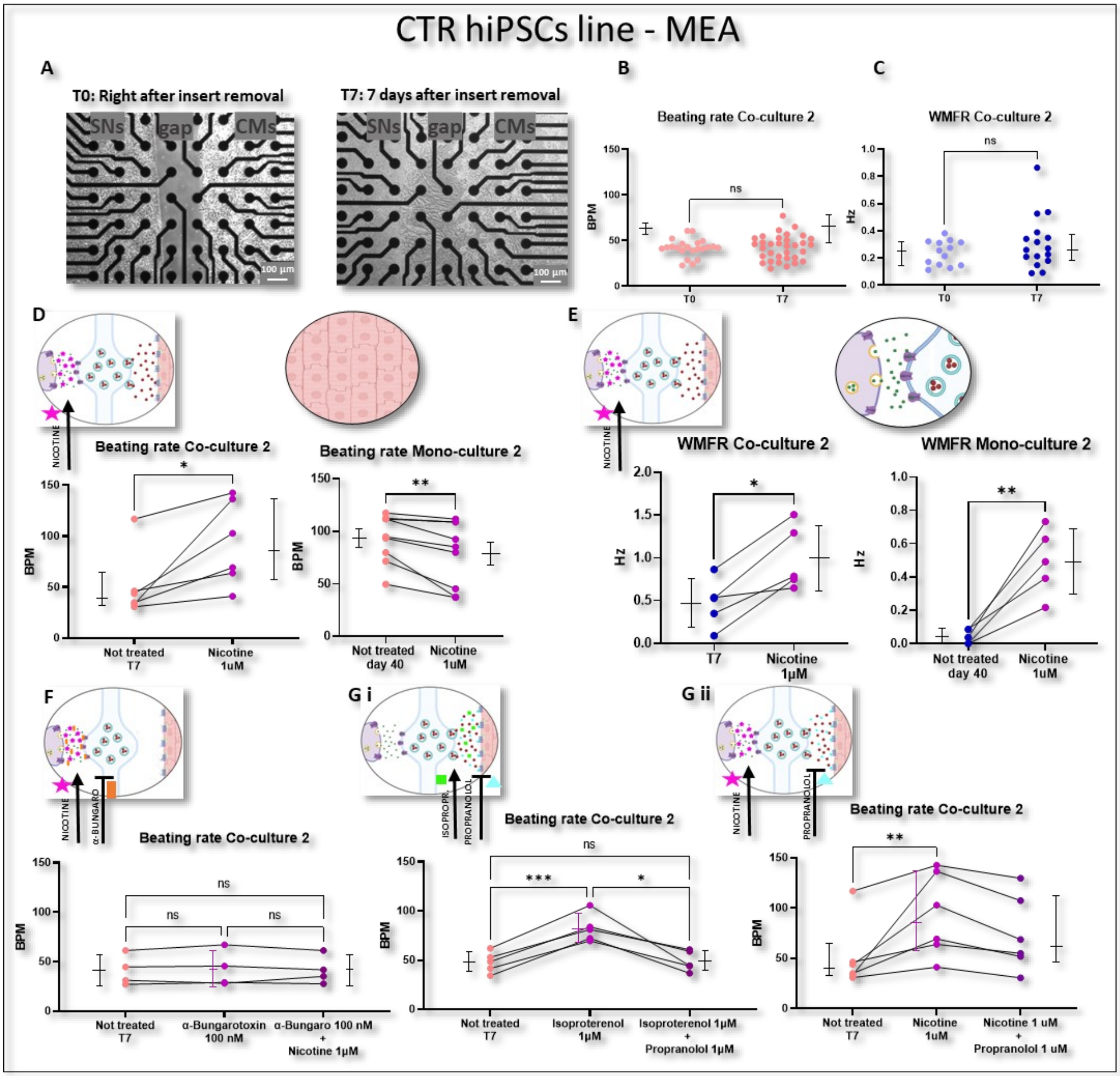

Figure S10: Functional analysis of co-cultures derived from a second CTR hiPSC line confirms that CTR hiPSC-SNs can modulate the beating activity of CTR hiPSC-CMs.

A: Brightfield images of co-cultures plated on MEA plates at the timepoint T0 and timepoint T7. Scale bar 100 µm.
B: Spontaneous beating rate of CTR hiPSC-CMs in co-cultures evaluated at timepoint T0 (no connections between the two populations) and timepoint T7. Median with IQR of 7 differentiations with 3-6 replicates- each dot represents a technical replicate. Test used: Mann Whitney test: not significant.
C: Spontaneous WMFR of CTR hiPSC-SNs in co-cultures evaluated at timepoint T0 (no connections between the two populations) and timepoint T7. Median with IQR of 11 differentiations with 1-3 replicates - each dot represents a technical replicate; Test used: Mann Whitney test: not significant.
D: Beating rate of CTR hiPSC-CMs in co-cultures at timepoint T7 treated with 1 µM Nic vs. not treated co-culture. Median with IQR of 6 differentiations with 1 replicate. Test used: Wilcoxon test; p value: * = 0.031.
Beating rate of CTR hiPSC-CMs in mono-cultures at day 40 treated with 1 µM Nic vs. not treated mono-culture. Mean with SD of 7 differentiations with 2 replicates - each dot represents one technical replicate. Test used: Paired-t test; p value: **

= 0.007.

E: WMFR of CTR hiPSC-SNs in co-culture/mono-culture treated with 1 µM Nic vs. not treated co-culture/mono-culture. Co-culture vs Not treated (Mean with SD of 5 co-cultures with 1-5 replicates- each dot represents a technical replicate). Test used: Paired t-test; p value: * = 0.01. Mono-culture vs Not treated (Mean with SD of 5 co-cultures with 2-6 replicates- each dot represents a technical replicate). Test used: Paired t-test; p value: * = 0.006.

F: Beating rate of CTR hiPSC-CMs in not treated co-cultures and treated with 100 nM α-Bungarotoxin or α-Bungarotoxin + Nic. Co-culture vs Not treated (Mean with SD of 4 co-cultures with 1 replicate). Test used: Tukey multiple comparison test: not significant.

G i: Beating rate of CTR hiPSC-CMs in co-cultures treated with Isoproterenol (1 µM) vs. Isoproterenol (1 µM) + Propranolol (1 µM) vs. not treated (Mean with SD of 5 co-cultures with 1 replicate). Test used: Tukey multiple comparisons test; p value= Not treated co-cultures vs. Isoproterenol, p value: *** = 0.0007; Isoproterenol vs. Isoproterenol + Propranolol, p value: * = 0.022.

G ii: Beating rate of CTR hiPSC-CMs in co-cultures treated with 1 µM Nic vs. 1 µM Nic + 1 µM Propranolol vs. not treated (Median with IQR of 6 co-cultures with 1 replicate- each dot represents a technical replicate), Test used: Dunn's multiple comparisons test; Not treated co-cultures vs. Nicotine, p value: ** = 0.004.

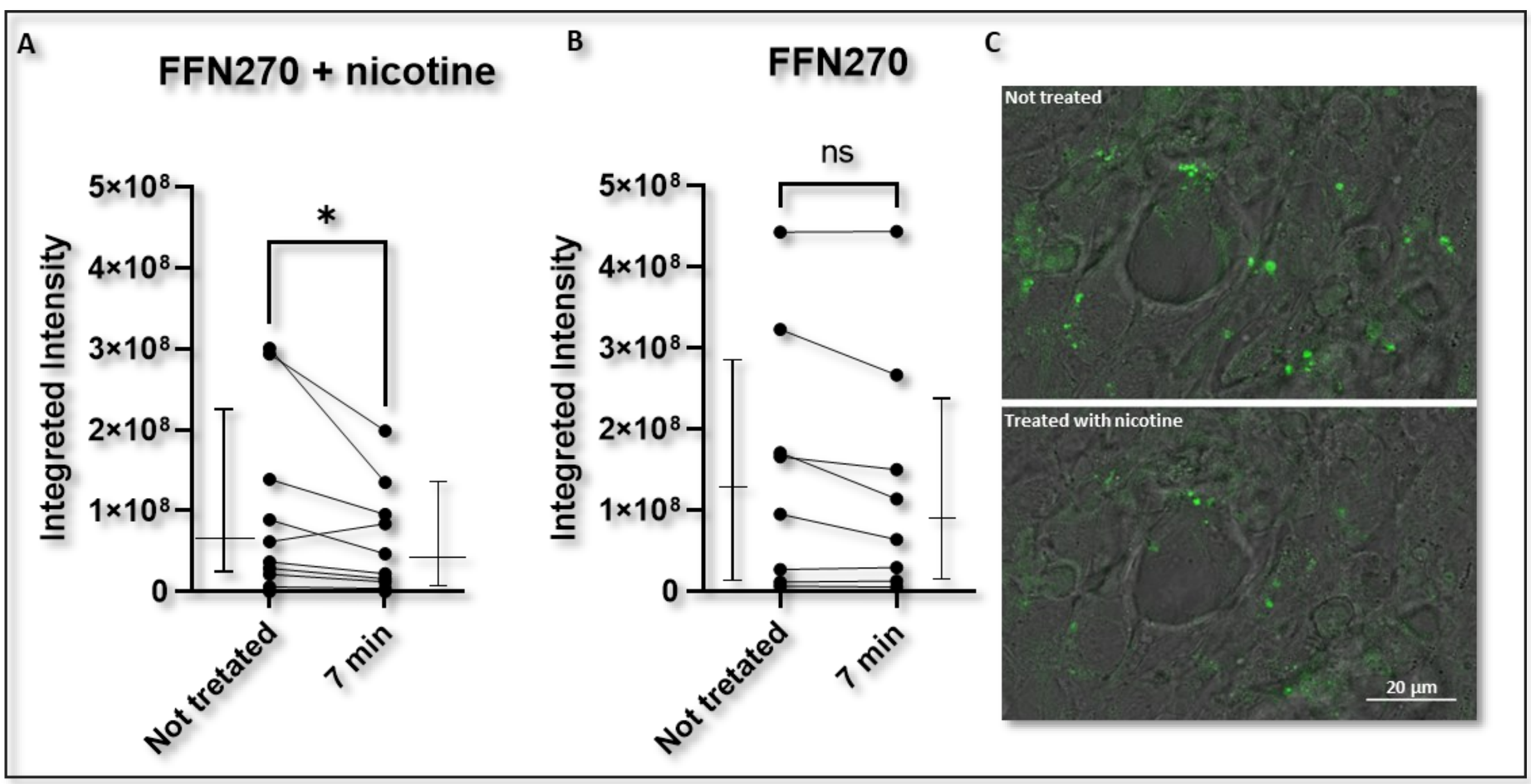

Figure S11: Integrated intensity analysis for the FFN270 assay

A: Co-cultures stained with 5 µM FNN270 and subsequently treated with 10 µM Nic. Images acquisition g was done prior to and 7 minutes following incubation (Median with IQR of 10 differentiations with 1 replicate). Test used: Paired t-test; p value: * = 0.0273.

B: Representation of control co-cultures stained with 5 µM FNN270, without Nic administration. Images acquisition was done prior to 7 minutes following incubation (Median with IQR of 8 differentiations with 1 replicate). Test used: Wilcoxon test, not significant

C: High-magnification images of the co-cultures stained with FFN270 before nicotine administration and after. Scale bar 20 µm.

Table S1: statistic for data not normally distributed.

Excel: “Table S1”

Table S2: statistic for data normally distributed.

Excel: “Table S2”

Table S3: Protocol for the differentiation of hiPSC-SN / CTR-SNs.

Day	Medium	Factors	Procedure
**Day 1**	KSR	LDN/SB	Medium change
**Day 2**	KSR	LDN/SB	Medium change
**Day 3**	KSR	LDN/SB/CH/DAPT /PD	Medium change
**Day 4**	KSR	LDN/SB/CH/DAPT/ PD/SHH/PMP	Medium change
**Day 5**	75% KSR/ 25% NNB	SB/CH/DAPT/PD/ SHH/PMP	Medium change
**Day 6**	75% KSR/ 25% NNB	CH/DAPT/PD/ SHH/PMP	Medium change
**Day 7**	50% KSR/ 50% NNB	CH/DAPT/PD/ SHH/PMP	Medium change
**Day 8**	50% KSR/ 50% NNB	CH/DAPT/PD/ SHH/PMP	Medium change
**Day 9**	25% KSR/ 75% NNB	SHH/PMP	Medium change
**Day 10**	25% KSR/ 75% NNB	SHH/PMP	Medium change
**Day 11**	NBB27	SHH/Pu/BMP4	Medium change
**Day 12**	NBB28	SHH/Pu/BMP4	**Replating**
**Day 13**	NBB29	SHH/Pu/BMP4	Medium change
**Day 14**	NBB30	BMP4	Medium change
**Day 15**	NBB31	BMP4	Medium change
**from Day 16**	NBB32	cAMP/AA/NGF/BDNF/ GDNF	Medium change

Table S4: List of factors used for the hiPSC-SN / CTR-SNs differentiation.

Factors	Complete Name	Brand	Final concentration
LDN	LDN-193189	Stemgent	500nM
SB	SB431542	Tocris	10uM
CH	CHIR99021	Stemgent	3uM
DAPT	DAPT	Tocris	10uM
PD	PD173074	Sigma Aldrich	0,2uM
SHH	Human Sonic Hedgehog	R&D System	100ng/mL
PMP	Purmorphamine	Stemgent	1uM
BMP4	Human Bone Morphogenetic Protein 4	R&D System	10ng/mL
cAMP	dibutyryl-cyclic-A	Enzo LifesciencesMP	0,2mM
AA	ascorbic acid	Sigma Aldrich	0,2mM
NGF	Human β-NGF	Peprotech	10ng/mL
BDNF	Human BDNF	Peprotech	20ng/mL
GDNF	Human GDNF	Peprotech	10ng/mL

Table S5: List of antibodies used in this work.

Excel: “Table S5”

Table S6:

TaqMan probes used in this work.

Gene	Fluorophore	Assay ID
**TH**	FAM	Hs00165941_m1
**DBH**	FAM	Hs01089840_m1
**RPL13**	FAM	Hs00366152_m1
**GAPDH**	FAM	Hs02786624_g1
**TNNI3**	FAM	Hs00165957_m1
**MYH7**	FAM	Hs01110632_m1
**β1AR**	FAM	Hs02330048_s1
**SEMA3C**	FAM	Hs00989373_m1
**NGF**	FAM	Hs00171458_m1
**GDNF**	FAM	Hs01931883_s1
**BDNF**	FAM	Hs02718934_s1
**HCN4**	FAM	Hs00975492_m1
**SCN5A**	FAM	Hs00165693_m1
**KCNA5**	FAM	Hs00969279_s1

Video:

Video S1: Timelapse video of a co-culture at 7 days. Magnification 10x.
