# Supplementary material for: A reproducible and physiologically relevant human iPSC-derived platform for in vitro modeling of the neurocardiac junction
Source: Mol Ther. 2026 Apr 1;34(8):4921–41. doi: 10.1016/j.ymthe.2026.03.034 (PMC13464303; doi:10.1016/j.ymthe.2026.03.034)
Supplement: Supplementary file 2 [file mmc6.pdf]

Molecular Therapy
Original Article

A reproducible and physiologically relevant human iPSC-derived platform for *in vitro* modeling of the neurocardiac junction

Giada Cattelan,[1,2] Claudia Altomare,[3,4,5] Giovanna Gentile,[1,2] Alexandros A. Lavdas,[1] Laura Sophie Frommelt,[1,6,7] Chiara Volani,[1,8] Luisa Foco,[1] Pietro Girardi,[1] Elisa Peducci,[1] Amparo Guerrero Gerboles,[9] Michele Miragoli,[9,10] Luisa Petti,[2] Peter P. Pramstaller,[1] Lucio Barile,[3,4,5] Serena Zacchigna,[6,7] Irene Pichler,[1] Alessandra Rossini,[1] Alessandra Zanon,[1,11] and Marzia De Bortoli[1,11]

[1]Eurac Research, Institute for Biomedicine, 39100 Bolzano, Italy; [2]Faculty of Engineering, Free University of Bolzano, 39100 Bolzano, Italy; [3]Cardiovascular Theranostics Group, Istituto Cardiocentro Ticino, Ente Ospedaliero Cantonale, 6900 Lugano, Switzerland; [4]Institute for Translational Research, Faculty of Biomedical Sciences, Università Svizzera Italiana, Ente Ospedaliero Cantonale, 6500 Bellinzona, Switzerland; [5]Euler Institute, Faculty of Biomedical Sciences, Università Svizzera Italiana, 6900 Lugano, Switzerland; [6]Cardiovascular Biology Laboratory, ICGEB Trieste, 34149 Trieste, Italy; [7]University of Trieste, Department of Medicine, Surgery, and Health Sciences, 34149 Trieste, Italy; [8]The Cell Physiology MiLab, Department of Biosciences, Universitá Degli Studi di Milano, 20133 Milano, Italy; [9]Department of Medicine and Surgery, University of Parma, 43124 Parma, Italy; [10]IRCCS, Humanitas Research Hospital Department of Medicine, Via Rita Levi Montalcini 4, Pieve Emanuele, 20072 Milan, Italy

The cardiac autonomic nervous system is central to various cardiac diseases, yet its regulation in the human heart remains poorly understood due to the lack of reliable models. Here, we report the development of a neurocardiac co-culture system using human induced pluripotent stem cell-derived cardiomyocytes (hiPSC-CMs) and sympathetic neurons (hiPSC-SNs). Both cell types were characterized molecularly and electrophysiologically and subsequently used for the establishment of the co-culture model in a two-well chambers insert, forming a dense axonal network connected with hiPSC-CMs. The co-culture demonstrated robust functional interactions: hiPSC-CMs maintained a stable beating rate, while hiPSC-SNs exhibited significantly increased firing activity after 7 days. Nicotine stimulation enhanced hiPSC-SN activity, resulting in an increased beating rate in co-cultured hiPSC-CMs, an effect that is absent in monoculture. This rise in the beats was abolished by nicotinic acetylcholine receptor blockade through α-bungarotoxin on hiPSC-SNs in co-culture. The β-blocker propranolol mitigated isoproterenol or nicotine effects on hiPSC-CMs. Using a fluorescent tracer, functional exocytosis and norepinephrine release was confirmed in hiPSC-SNs in co-culture. This novel neurocardiac model replicates neuronal control of cardiomyocytes and provides a new and robust platform for studying neurocardiac interactions. It represents a promising tool for advancing disease modeling and pharmacologic research in cardiac pathophysiology.

INTRODUCTION

The heart has a "little brain"[1] that consists of various subtypes of neural interplayers, including two external innervations, the sympathetic and the parasympathetic systems, and an intrinsic population of neurons resident in the cardiac ganglia. All these components regulate cardiac activity and form a complex multi-level neural network, which constitutes the cardiac autonomic nervous system (CANS).[1]

The mutual "accentuated antagonism" between the sympathetic and parasympathetic systems is responsible for the healthy physiological status of the organism.[2–4] Indeed, the sympathetic nervous system mediates the "fight or flight" response, whereas the parasympathetic system mediates the "rest and digest" response.

In the cardiac context, activation of the sympathetic nervous system leads to cardiac chronotropic and inotropic effects,[5,6] while tuning the conduction velocity of myocardial tissue.[7–9]

Sympathetic innervation is divided into preganglionic and postganglionic efferents. Once activated, preganglionic neurons release acetylcholine,[10] which binds to acetylcholine-nicotinic receptors (nAchR) on the postganglionic neurons. The postganglionic efferents release norepinephrine,[10,11] the principal neurotransmitter of sympathetic neurons (SNs), which activates β-adrenergic receptors (βAR) at the level of the neurocardiac junction (NCJ),[12] increases

Received 24 January 2025; accepted 26 March 2026;
https://doi.org/10.1016/j.ymthe.2026.03.034.

[11]These authors contributed equally

Correspondence: Giada Cattelan, Eurac Research, Institute for Biomedicine, 39100 Bolzano, Italy.
E-mail: giada.cattelan@eurac.edu
Correspondence: Marzia De Bortoli, Eurac Research, Institute for Biomedicine, 39100 Bolzano, Italy.
E-mail: marzia.debortoli@eurac.edu

© 2026 The Authors.
Published by Elsevier Inc. on behalf of The American Society of Gene and Cell Therapy.
This is an open access article under the CC BY license (http://creativecommons.org/licenses/by/4.0/).

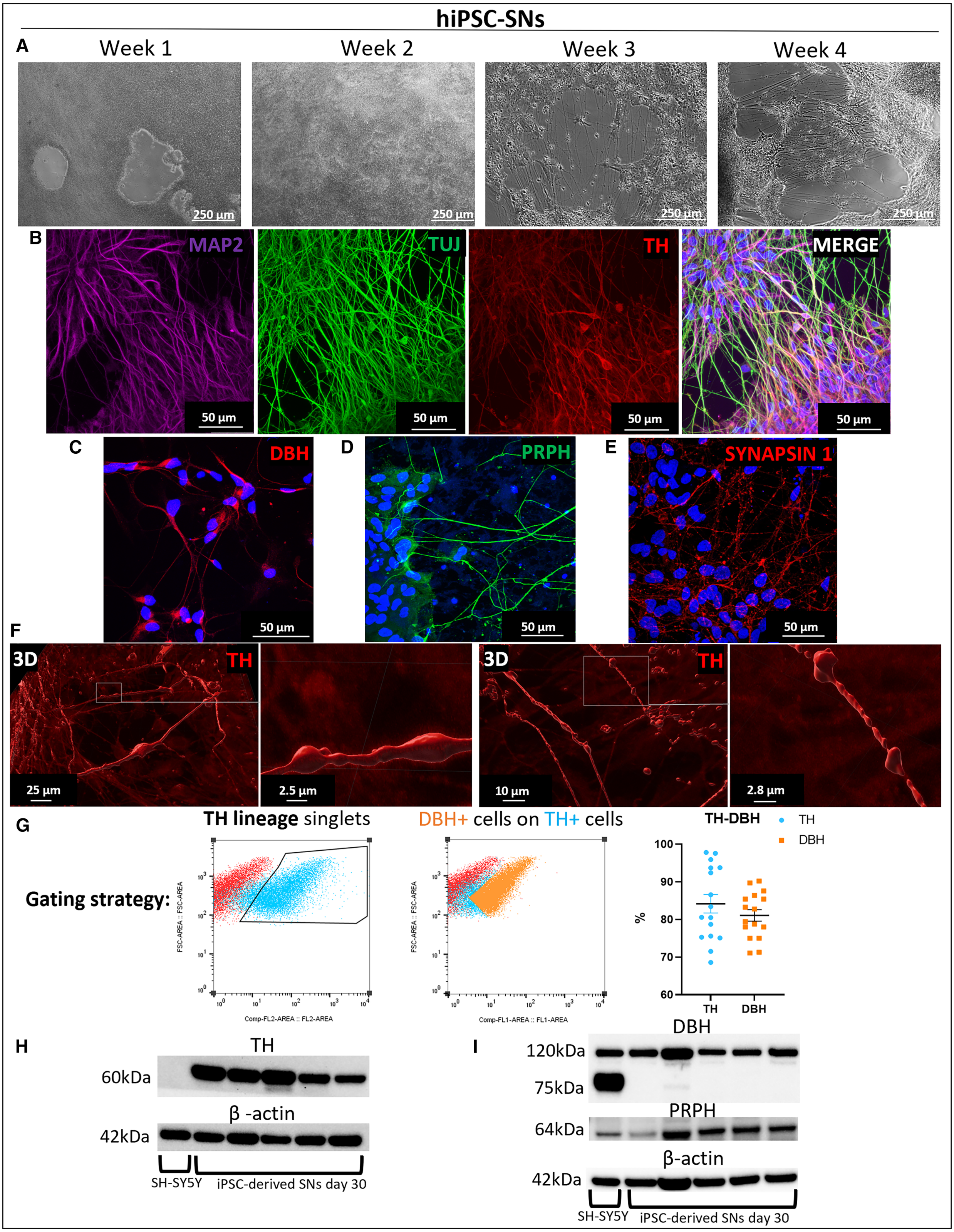

(legend on next page)

intracellular cyclic AMP (cAMP),[13,14] and modulates calcium-mediated mechanisms.[15,16] The postganglionic SNs project axons to several cardiac compartments, such as the sinoatrial and atrioventricular nodes.[7,8,17,18] The precise autonomic control of all these cardiac compartments is possible thanks to the "pearl necklace" conformation of the sympathetic innervation, where each of these "pearls" is a varicosity containing the synaptic vesicles for the neurotransmitter release.[19]

The precise way that neurons use to communicate with cardiac cells at the NCJ has been described in several studies.[7–9,20,21] However, many mechanisms of interaction and intercommunication between these two cellular populations remain unclear due to the lack of an effective human model that could recapitulate the features of NCJ.[22] Although animal models have been used in several disease research studies, their translational relevance in human medicine is limited by species-specific differences. Moreover, the access to human heart or brain tissues remains constrained by the necessity for invasive procedures and the relative ethical concerns. Therefore, bridging this gap between animal and human models remains a critical pursuit in biomedical research.[23] Human induced pluripotent stem cells (hiPSCs) technology represents an unlimited source of human materials.[24] Among the many applications of the hiPSCs, one of the most significant is their use in developing human models.[25] Despite this potential, few neurocardiac co-culture systems based entirely on hiPSC-derived neurons and cardiomyocytes (CMs) have been reported.[7,8,26–28] Early approaches, including a hybrid model proposed by Oh et al.,[29] combining hiPSC-derived SNs with neonatal mouse CMs, established a widely used differentiation protocol later refined in neurocardiac co-culture systems, as seen in the works of Winbo et al. describing the characterization of a functional neurocardiac co-culture entirely based on hiPSC-derived cells.[26] Here, we present the creation of a compartmentalized and functional human neurocardiac co-culture model, using a simple two-chamber silicone insert. This cost-effective and reproducible system enables the study of human NCJ and demonstrates that hiPSC-derived SNs functionally interact with hiPSC-derived CMs and modulate their activity.

RESULTS

Morphological and molecular characterization of hiPSC-SNs

Prior to the evaluation of the co-culture coupling efficiency, we assessed the molecular and functional properties of neurons and CMs in monoculture.

During the first 2 weeks of sympathetic neural differentiation, cells continued to proliferate until reaching 100% confluence (Figure 1A). To prevent overgrowth, the hiPSC-derived neural progenitors were replated on day 12 by cutting the monolayer into small blocks. During the third and fourth weeks, the cells developed the typical neuronal morphology, characterized by elliptical cell bodies, dendritic-like processes, and long axons, which progressively formed an intricate network (Figures 1A and 1B). To characterize hiPSC-SNs, we used different markers (Figures 1B–1E). As expected, at day 30 of differentiation, hiPSC-SNs displayed an extensive axonal network positive for microtubule-associated protein 2 (MAP2) and class III member of the β-tubulin family (TUJ1), two pan-neuronal markers of mature neurons, which stained the soma and dendrites of most neurons. hiPSC-SNs showed also positivity to tyrosine hydroxylase (TH) (Figure 1B) and dopamine β-hydroxylase (DBH) (Figure 1C). In particular, TH and DBH are key enzymes typically expressed in SNs, responsible for the conversion of L-tyrosine to L-dopa and dopamine to L-norepinephrine, respectively.[29–33] Importantly, this cellular population also tested positive for peripherin (PRPH), a typical marker of peripheral neurons, supporting their peripheral lineage identity (Figure 1D).[33] Finally, the presence of presynaptic vesicles was confirmed by the punctate staining of the phosphoprotein synapsin 1, which is present on the surface of the synaptic vesicles (Figure 1E).[21] Notably, three-dimensional (3D) reconstruction of TH staining revealed pearl-like varicosities along neurites, typical sites of neurotransmitter storage and release, evident by day 30 (Figure 1F).[19]

In addition, we quantified the percentage of TH^+ and DBH^+ cells present in the neural population by flow cytometry. At day 30 of differentiation, 68%–97% of the cells were TH^+, 71%–90% of which were also positive for DBH (DBH^+) (Figure 1G). Finally, the high abundance of TH^+/DBH^+ neurons was further confirmed by western blot analysis. hiPSC-SNs abundantly and consistently expressed both TH and DBH proteins at day 30 of differentiation, confirming the immunofluorescence and flow cytometry results (Figures 1H and 1I). Non-differentiated SH-SY5Y cells expressed two different forms of DBH, the soluble form (lower band) and the membrane-bound form (higher band). hiPSC-derived SNs predominantly exhibit the membrane-bound form, which represents the active form of DBH, able to convert dopamine to norepinephrine.[34] Furthermore, PRPH protein expression in hiPSC-SNs was also confirmed by

Figure 1. Morphological and immunofluorescence characterization of hiPSC-SNs: hiPSC-SNs show typical marker expression of SNs with different methods

(A) Representative bright-field images of hiPSC-SNs at different time points of the sympathetic neural differentiation protocol. Scale bar, 250 μm. (B) Qualitative immunostaining images of hiPSC-SNs marked with different markers. MAP2 (purple)/tubulin J (green)/TH (red) co-staining of hiPSC-SNs at day 30. Nuclei were counterstained with DAPI (blue). Scale bar, 50 μm. (C–E) Qualitative immunostaining images of hiPSC-SNs marked independently with DBH, PRPH, and synapsin 1 at day 30 of differentiation. Nuclei were counterstained with DAPI (blue). Scale bar, 50 μm. (F) The 3D reconstruction of confocal images of hiPSC-SNs at day 30 stained with TH displaying varicosities. Scale bars, 25, 2.5, 10, and 2.8 μm. (G) Flow cytometry analysis of hiPSC-SNs at day 30 of differentiation. The gating strategy for hiPSC-SNs showed a first gating for TH^+ cells (FSC-A vs. SSC-A). A second gating for the exclusion of the doublets was applied to the first gating (FSC-A vs. FSC-W). Lastly, the marker for the identification of DBH^+ cells was selected. Population in red: isotype control; population in blue: TH^+; population in orange: DBH^+. The graph shows the percentage values of TH^+/DBH^+ cells at day 30 for each experiment performed. Mean with SEM of 16 differentiations: TH, 68.6%–97.8%; DBH, 71.1%–90.2%. (H and I) Qualitative western blot analysis of hiPSC-SNs at day 30 of differentiation for TH (H) and DBH (higher band: membrane bound; lower band: soluble form) and PRPH (I). β-Actin was used as loading control and undifferentiated SH-SY5Y cells as internal positive (DBH and PRPH) or negative (TH) control. Each band of hiPSC-SNs represents a different differentiation.

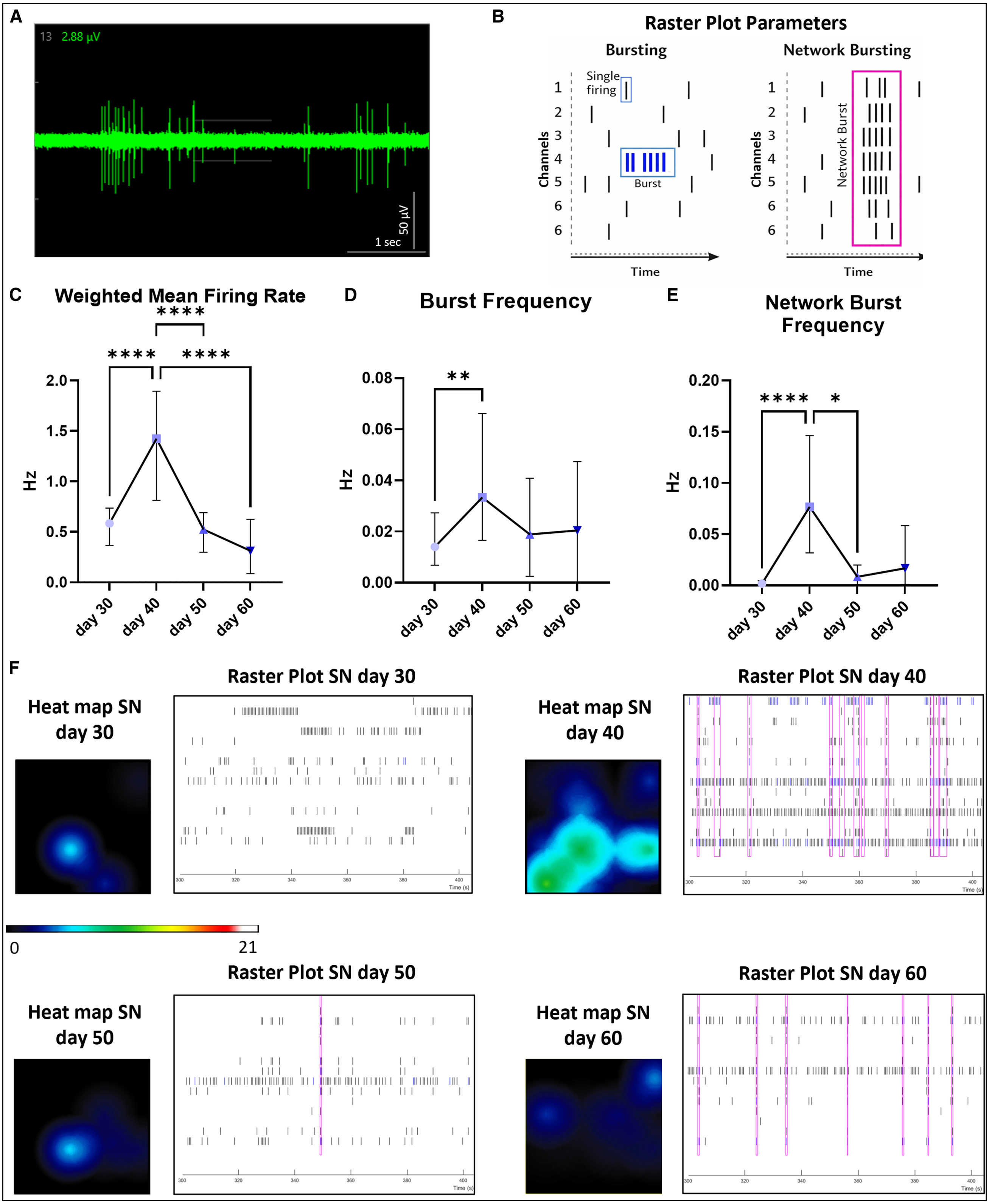

(legend on next page)

western blotting (Figure 1I). For TH protein expression, undifferentiated SH-SY5Y cells were used as negative control (CTR), as they do not express TH.[35] Conversely, they were used as positive CTRs for DBH and PRPH because they display a strong adrenergic phenotype.[36–38]

Electrophysiologic characterization of hiPSC-SNs indicates a basal sympathetic firing activity

To functionally assess SN activity, electrophysiologic recordings using the multi-electrode array (MEA) system were performed every 10 days from day 30 to day 60 of differentiation (Figure 2). Raw signal (Figure 2A) was converted into raster plots (Figure 2B) showing a single spike on a single electrode (black single vertical line), bursts on a single electrode (blue single vertical line; spikes cluster over a period of time), and network bursts across multiple electrodes (pink rectangle; coordinated cluster of spiking) (Figure 2B). MEA analysis revealed that hiPSC-SNs exhibited the highest weighted mean firing rate (WMFR) at day 40 (Figure 2C) of neural differentiation compared with the other time points (Table S1) (Figure 2C). At day 40, hiPSC-SNs also presented the highest burst frequency (Figure 2D) and network burst frequency (Figure 2E) among all the time points (Figure 2F; Table S1).

Live and dead assay was performed to investigate the viability of hiPSC-SNs and to see whether there was a correlation with the fluctuation of the electrical activity of the neurons during the time (Figures S1A–S1D). At day 40 of differentiation, the highest percentage cell death (35.59%) was observed compared with the other time points (23.97% at day 30, 31.66% at day 50, and 18.22% at day 60) (Figure S1E; Table S1). These analyses also showed that at days 50 and 60, the percentage of live cells was relatively high, corresponding to 64.11% and 81.78%, respectively (Figure S1F; Table S1).

The maximum functionality observed at day 40 is likely attributable to a pruning effect known to refine the neuronal network, rather than neuronal loss on subsequent days (50 and 60), as indicated by viability data. For this reason, we selected this time point as the most appropriate for further experiments.

Generation and characterization of hiPSC-CMs

After approximately 30 days of differentiation, hiPSC-CMs were stained for specific cardiac markers, namely sarcomeric α-actinin and cardiac troponin I (Figure 3A). hiPSC-CMs exhibited an organized sarcomeric structure—typical cardiac muscle banding among different time points (Figure S2). The expression of the cardiac marker genes myosin heavy chain 7 (*MYH7*), troponin I, and β-1 adrenergic receptor (*β1AR*) was assessed by quantitative real-time PCR at days 30 and 40 of differentiation (Figures 3B–3D; Tables S1 and S2). *MYH7* gene encodes the β-myosin heavy chain (another sarcomeric protein),[39] whereas *β1AR* encodes CM membrane receptors that bind products of the catecholamine pathway, such as dopamine and norepinephrine.[11,21] The expression of each gene was analyzed without identifying significant changes over time (Figures 3B–3D). Electrical activity was recorded using the MEA system for evaluating the functional properties of hiPSC-CMs. At day 30, hiPSC-CMs displayed a mean beating rate of 53.74 ± 4.28 bpm, which increased and stabilized at 40 days of culture, with 65.52 ± 4.42 bpm, and at day 50 with, 64.06 ± 4.42 bpm (Figure 3E). Subsequently, hiPSC-CMs at day 40 were exposed to 1 μM isoproterenol (Iso), a well-known β1AR agonist, resulting in a significant increase in the beating rate ($p = 0.0156$; Table S1).

Creation of a compartmentalized co-culture of hiPSC-SNs and hiPSC-CMs

To set up the neurocardiac co-culture model, we used a silicone insert divided into two chambers, allowing us to plate the two different cell populations physically separated from each other. Removal of the insert enabled the establishment of a co-culture composed of the two different interacting cell populations within approximately 3 days. A schematic representation of the protocol is shown in Figures 4A and 4B. Among the various cell densities tested (Figure S3), 9×10^4 cells per chamber of hiPSC-SNs and hiPSC-CMs were chosen to maximize the probability of forming a compact monolayer of both cell types (Figure 4B). The co-cultures were maintained in a 1:1 medium mixture of neural and CM media, previously described as optimal for supporting the culture of both cell populations, promoting neurite growth, and sustaining CM beating.[26] One important aspect evaluated for the setup of the co-culture model was the coating strategy. After insert removal, establishing connections between hiPSC-SNs and hiPSC-CMs was extremely difficult due to the uncoated gap between the two cell populations. For this reason, a drop of Matrigel (0.5 mg/mL) was applied to the cells. This step was found to be a crucial prerequisite for the formation of a connecting co-culture within 3–4 days from the removal of the insert.

For the co-culture experiments, the baseline time point T0 corresponds to insert removal, marking the start of the effective co-culture conditions (at this time, the two cell populations are not connected). hiPSC-SNs and hiPSC-CMs were then maintained in co-culture for up to 7 days (T7), corresponding approximately to days 37–40 of the monoculture (Video S1).

Figure 2. Long-term functional profile of hiPSC-SNs analyzed with MEA instrument

(A) Representative raw signal of the firing activity of hiPSC-SNs recorded by a single electrode. Scale bar, $x = 1$ s; $y = 50$ μV. (B) Scheme of raster plot parameters. Channels, different electrodes of one well; black vertical line, one spike on one electrode; blue vertical lines, bursts on one electrode; pink rectangle, network burst activity among the entire well. (C) WMFR. Interquartile range (IQR) with median of six differentiations. Test used: Dunn's multiple comparisons test; ****$p < 0.0001$. (D) Burst frequency. IQR with median of six differentiations. Test used: Dunn's multiple comparisons test; **$p = 0.0089$. (E) Network burst frequency. IQR with median of six differentiations. Test used: Dunn's multiple comparisons test; p value: day 30 vs. day 40 *$p = 0.0297$; ****$p < 0.0001$. (F) Representative heatmaps and raster plots of hiPSC-SNs at 30, 40, 50, and 60 days of differentiation.

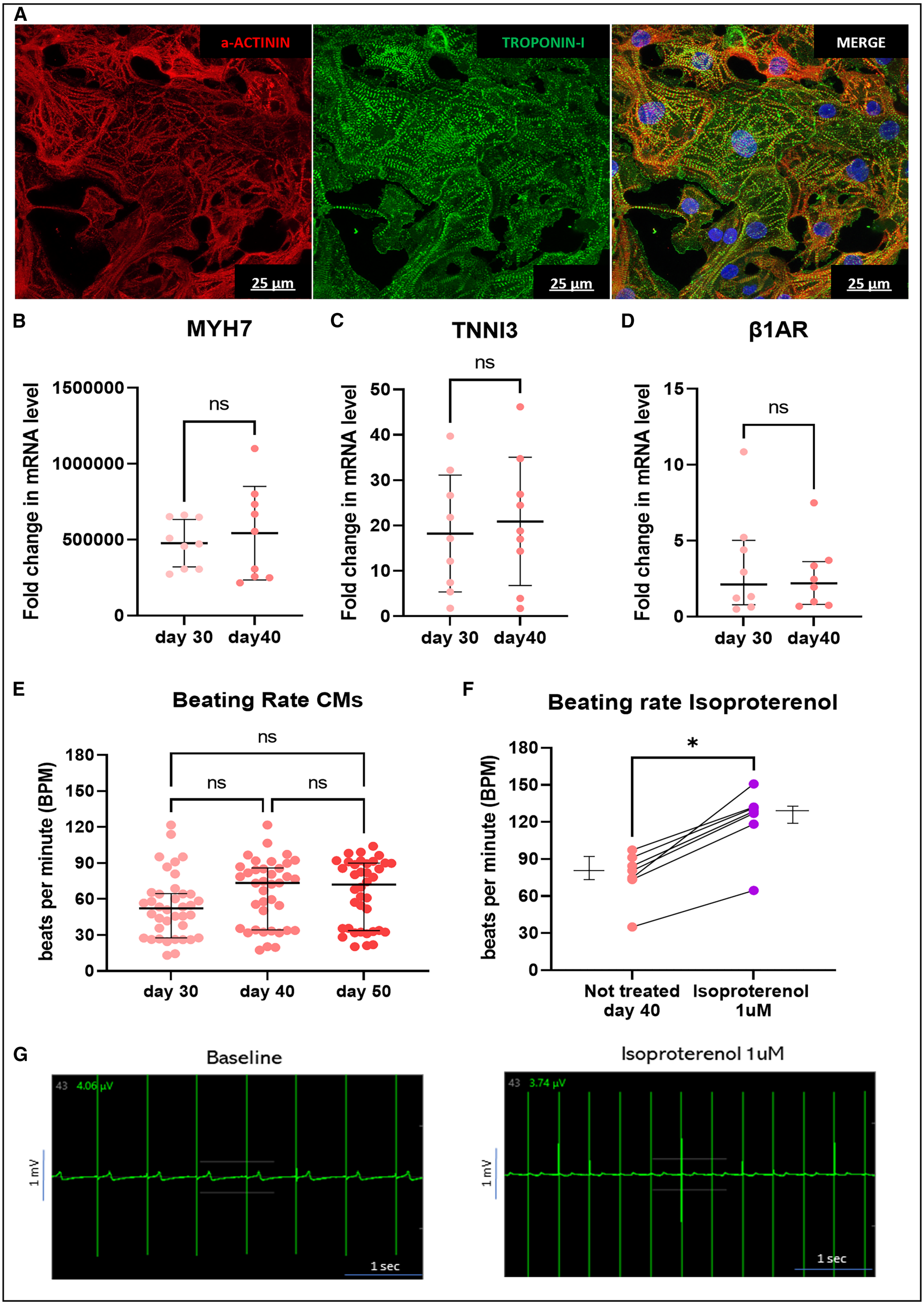

(legend on next page)

Immunocytochemical and molecular characterization of the co-culture model

Immunofluorescence staining was performed as an initial characterization of the co-culture model at day 7 (Figures 5 and 6). The sarcomeric α-actinin was used as marker for hiPSC-CMs, exhibiting a well-organized sarcomeric structure (Figures 5 and 6). For hiPSC-SNs, different markers were analyzed (Figures 5 and 6). Figure 5 displays a representative large area of the co-culture assembled by a collage of single frames, showing the exact region where the two populations meet. In Figure 5, it is also possible to observe that the neural population projects axons toward the whole cardiac population, with TH^+ neurons assembling into thick bundles. The intricate network of innervation appears denser where the CMs are in closer proximity to the neurons, whereas in the center of the cardiac cell population, the axon bundles become thinner. The thick axon bundles became thin single axons penetrating in-depth and innervating one or more hiPSC-CMs at the same time (Figure 5). At higher magnification, it is possible to observe in greater detail the neurocardiac co-cultures showing positivity for DBH and PRPH markers, staining the axonal prolongations connected with the cardiac population (Figures 6A and 6B). Subsequently, co-cultures were stained with synapsin 1 to observe the pre-synaptic cell contacts. Figure 6C displays an accumulation of synapsin 1 around the hiPSC-CMs, attributable to the axonal contact with cardiac cells. In the 3D reconstruction, synapsin 1, which is uniquely present in the membrane of pre-synaptic vesicles, appears clearly in puncta-like structures, suggesting the formation of synaptic clusters in the site of neuronal-cardiac interaction (Figure 6F). Axonal distribution and structure were observed thanks to the 3D reconstruction of TH (Figure 6G) and MAP2 markers (Figure 6H). The cytoskeletal protein MAP2 marks all neurons, but not all neuronal cell types are TH^+, as it is specific for some neuronal subpopulations, such as sympathetic or dopaminergic neurons. The 3D reconstruction of TH staining highlighted the presence of an abundant number of TH^+ varicosities in a pearl-like form, evidencing the mature-like phenotype of hiPSC-SNs (Figures 6G and 6H). Furthermore, we investigated specific receptors of both cell types. For hiPSC-SNs the presence of nAChRs was visualized by staining with α-bungarotoxin, a selective and irreversible toxin, which binds nAChRs and blocks them at the α-subunit site (Figure 6D). The α-bungarotoxin staining was detected along the varicosities of the axons and colocalized with TH staining (Figure 6D). On hiPSC-CMs the presence of β1AR receptors was confirmed, showing a punctuated pattern (Figure 6E). Gene expression analysis revealed that *TH* and *DBH* expression was significantly increased after 7 days of co-culture ($TH = p$ value: 0.0002; $DBH = p$ value: 0.048; Figures S4A and S4B; Tables S1 and S2). A slight but not significant increase in the expression of *β1AR* genes was observed after 7 days of co-culture in comparison with the time point T0 ($\beta 1AR = p$ value: not significant; Figure S4C; Table S1). Further gene expression analysis for ion channels (Figure S5) and axon guidance (Figure S6) genes was performed to assess potential differences between monocultured hiPSC-CMs at day 37 and the same hiPSC-CMs after 7 days of co-culture with hiPSC-SNs, corresponding to the same stage of differentiation. Three representative cardiac ion channel genes were selected: *HCN4* (hyperpolarization activated cyclic nucleotide gated potassium channel 4), *KCNA5* (potassium voltage-gated channel), and *SCN5A* (sodium voltage-gated channel). Quantitative analysis revealed no significant differences in the expression between the monoculture and the co-culture CMs (Figure S5). However, *HCN4* and *KCNA5* genes showed a trend toward increased expression in co-culture, although this did not reach statistical significance (Figures S5A and S5B), whereas *SCN5A* expression remains unchanged (Figure S5C; Tables S1 and S2).

Axon guidance cues can exert both attractive and repulsive influences, thereby directing axonal projections toward their target cells. Among these, semaphorin 3C (*SEMA3C*) has been identified as a key attractant in the migration of cardiac neural crest cells, while neurotrophins such as brain-derived neurotrophic factor (BDNF), nerve growth factor (NGF), and glial cell line-derived neurotrophic factor (GDNF) are well known to be potent chemoattractants for promoting axonal growth and neuronal survival. In our system, although not reaching statistical significance, a trend toward increased expression of SEMA3C, BDNF, and NGF in hiPSC-CMs co-cultured with SNs was observed. Of note, *GDNF* expression was significantly upregulated in hiPSC-CMs after 7 days of co-culture compared with monocultures at day 37 (Figure S6; Tables S1 and S2). To further confirm the robustness of this NCJ model, we created new co-cultures with SNs and CMs derived from an additional hiPSC line (CTR hiPSC and EURACi009-A).[40,41] These new CTR co-cultures present the same behavior of the co-cultures created with the commercial cell line. Morphologic and molecular analyses confirm the deeply sympathetic innervation of the cardiac cell population and the consistent increased trends in the expression of the TH and DBH genes (Figures S7 and S8; Tables S1 and S2). These findings are in line with those observed in the commercial cell line (Figures 5, 6, and S4).

The neurocardiac co-culture model shows functional coupling under drug treatment

The ANS and the NCJ can be schematized as shown in Figure 7A. Thanks to the geometry of the co-culture created by the two-chamber

Figure 3. hiPSC-CMs show typical marker expression of CMs and electrophysiological response to isoproterenol

(A) A representative immunostaining of hiPSC-CMs (day 30) with α-actinin (red), troponin I (green), and DAPI for the nuclei staining. Scale bars, 25 μm. (B and C) Quantitative real-time PCR analysis of cardiac genes expression in hiPSC-CMs at 30 and 40 days. Mean with SD of eight to nine monocultures with one replicate of hiPSC-CMs. Test used: paired *t* test: not significant. (D) Quantitative real-time PCR analysis of cardiac genes expression in hiPSC-CMs at 30 and 40 days. IQR with median of eight to nine monocultures with one replicate of hiPSC-CMs. Test used: Wilcoxon test, not significant. (E) Baseline beating rate analysis of hiPSC-CMs at days 30, 40, and 50 of differentiation. IQR with median of seven monocultures with four to six replicates. Test used: Dunn's multiple comparisons test, not significant. (F and G) Beating rate analysis at day 40 before and after 1 μM isoproterenol (Iso) treatment. Median with IQR of seven differentiations with one replicate. Test used: Wilcoxon test; $^*p = 0.0156$. Scale bars, $x = 1$ s; $y = 1$ mV.

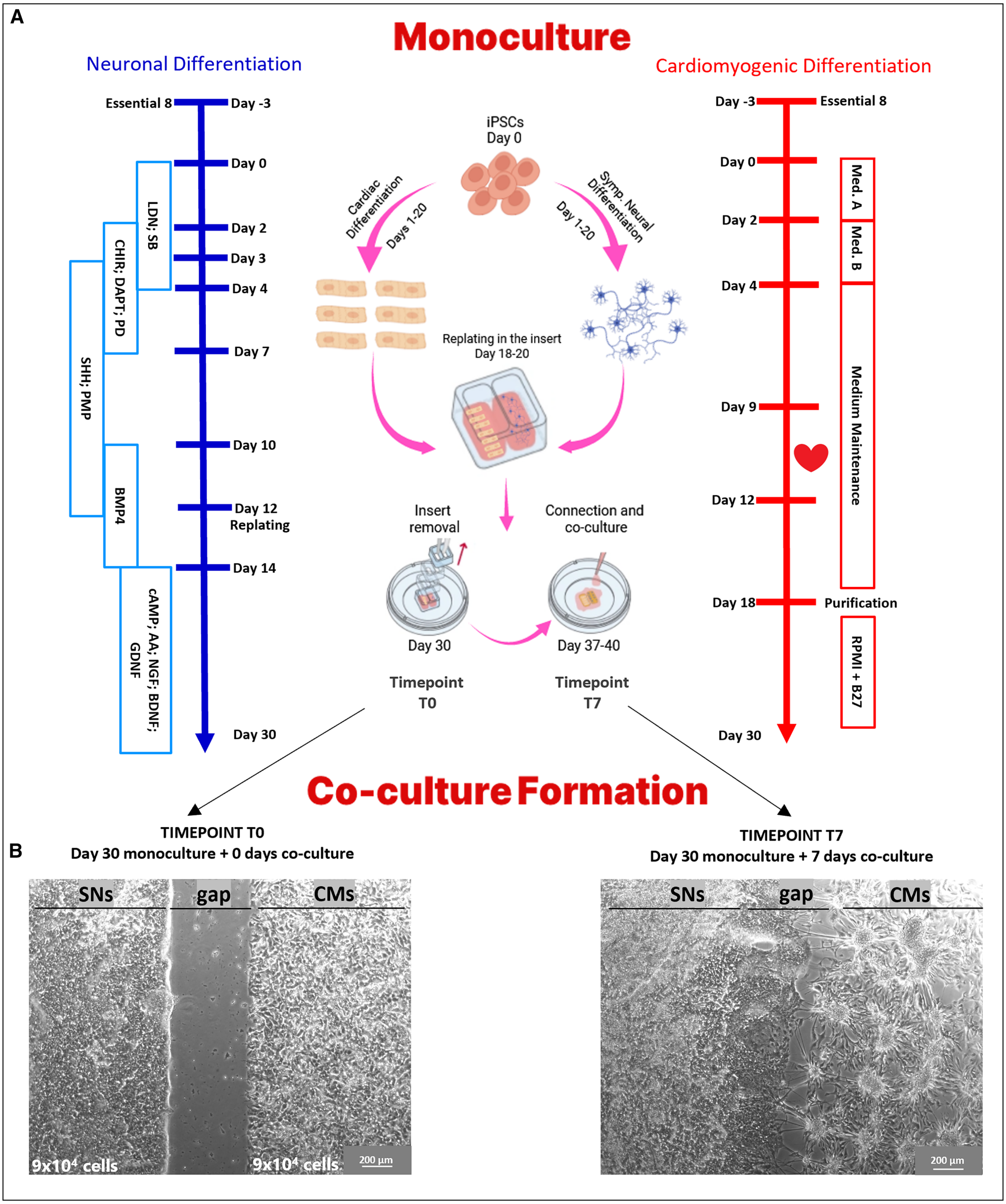

Figure 4. Schematic representation of the strategy for the co-culture generation

(A) Schematic representation of the cardiomyogenic and neural differentiation and co-culture setup protocol. (B) Bright-field images of the co-cultures at time point T0, showing the division of the two cell populations in two distinct areas, and the connection of the co-culture at time point T7 following the removal of the insert. Scale bars, 200 μm. Scheme created using BioRender.com.

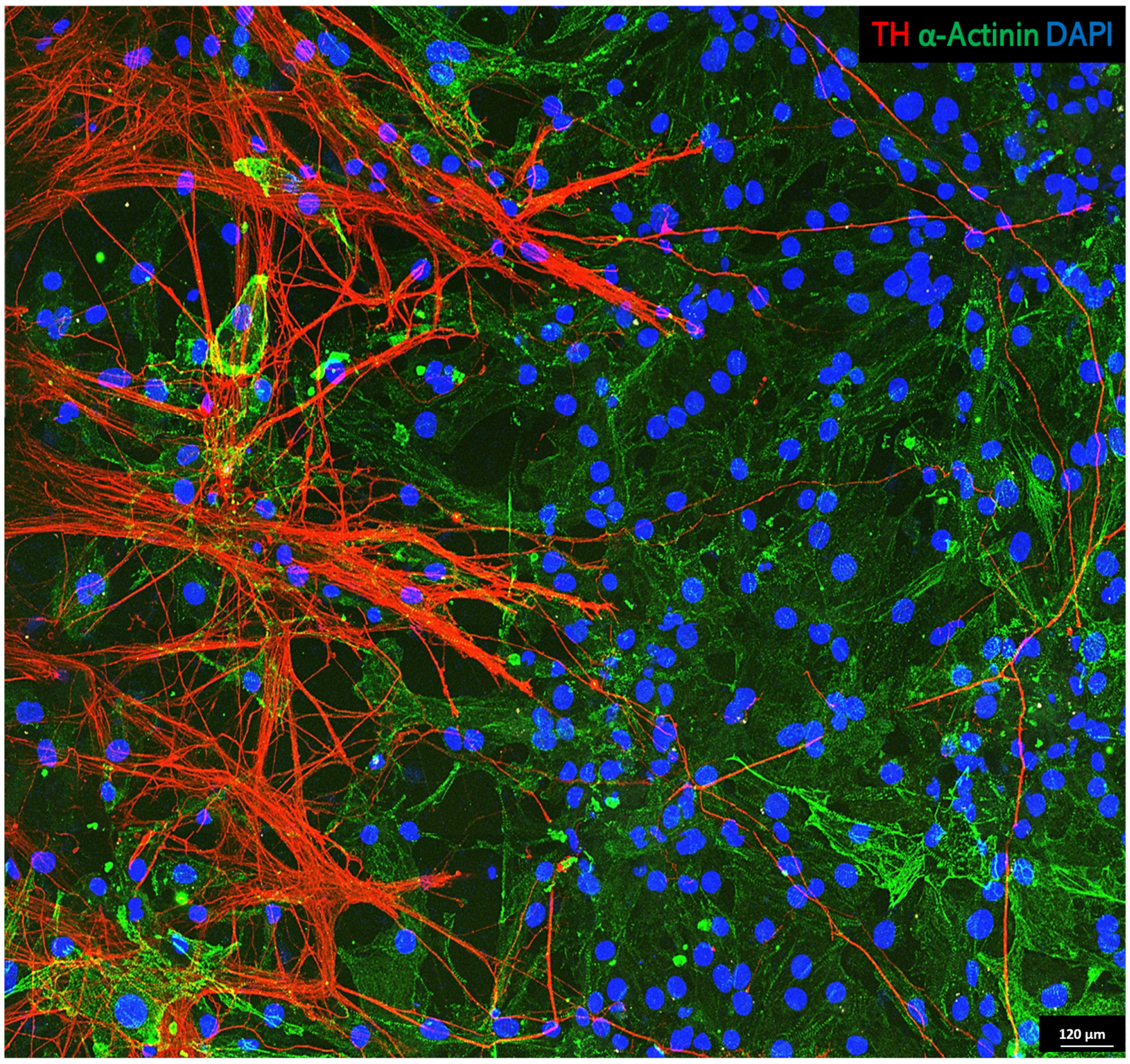

Figure 5. Reconstruction of a large area of a neurocardiac co-culture

Immunostaining experiment showing neuronal axons in red (TH) and CMs in green (sarcomeric α-actinin). Nuclei were stained with DAPI (blue). Scale bar, 120 μm.

insert and the distribution of the electrodes on the MEA wells, it was possible to select which electrodes (or group of electrodes) to use for the measurements, allowing an independent analysis for the two cell populations (Figure 7B). Compared with time point T0, baseline measurements indicated no significant variation in the beating rate of hiPSC-CMs after 7 days of co-culture with hiPSC-SNs (Figure 7C; Table S1). The monoculture of hiPSC-CMs was tested with co-culture medium to exclude the variation of the beating rate caused by the components of the neural medium. A detailed statistical analysis was carried out, showing no significant changes in the beating rate (Figure S9); hiPSC-SNs co-cultured for 7 days exhibited a significant increase in the WMFR (T0 vs. T7 = $p < 0.01$; Figure 7D; Table S1). However, the increased WMFR of hiPSC-SNs in co-culture is still not sufficient to modulate the frequency of the beating rate of hiPSC-CMs.

To assess the potential modulation of cardiac activity through the activation of hiPSC-SNs, we administered nicotine (Nic) to the co-cultures at T7. As confirmation of the establishment of a functional connection between hiPSC-SNs and hiPSC-CMs, we observed a significant increase in the hiPSC-CMs beating rate after Nic stimulation compared with the not-treated co-culture (not treated vs. Nic = $p < 0.01$; Figure 7E; Table S1), suggesting a physiological modulation of the CMs by the activated neural cell population. As expected, Nic stimulation on the monoculture of hiPSC-CMs at day 40 did not trigger any increase in the beating rate (Figure 7E; Table S2).[15,21,26,42,43] Of note, the WMFR of hiPSC-SNs increased after Nic administration both in co-culture and monoculture (co-culture: not treated vs. Nic = p value: 0.02; monoculture: not treated vs. Nic = p value: 0.021; Figure 7F; Table S1). To further clarify the functional mechanism between the cardiac and neuronal cells, after blocking the neural activity by using 100 nM α-bungarotoxin, we stimulated the co-cultures with 1 μM Nic. No change in the beating rate was observed in samples treated either with α-bungarotoxin or α-bungarotoxin and Nic (α-bungarotoxin vs. α-bungarotoxin + Nic = p value: not significant; Figure 7G; Table S2), confirming that the toxin effectively blocked the neural signal transmission to the cardiac cells by preventing the neuronal modulation of the CM beating rate. In addition, to assess the cardiac counterpart of the co-culture, we mimicked the release of norepinephrine from hiPSC-SNs at the level of the NCJ by adding 1 μM Iso, a β1AR agonist, either with or without propranolol (Prop, 1 μM), a β-blocker drug. As expected, hiPSC-CMs responded positively to Iso treatment with a significant increase in the beating rate, which was reversed by Prop administration. This markedly reduced the basal beating rate value (not treated vs. Iso = p value: 0.0092; Iso vs. Iso + Prop = p value: 0.0017; Figure 7H i; Table S2). To functionally test the entire co-culture model and observe the transmission of the signal from the hiPSC-SNs to the hiPSC-CMs, thereby capturing the full interaction between the two cell populations, we treated the co-cultures with Nic to trigger the hyperactivation of the hiPSC-SNs. After 2 min, we inhibited the β1AR with Prop to block the action of the neurotransmitters on hiPSC-CMs. As previously stated, Nic was able to significantly increase the beating rate of hiPSC-CMs in co-culture (not treated vs. Nic = p value: 0.048; Table S1). Upon Prop administration, the beating rate of hiPSC-CMs decreased reaching almost the basal levels (not treated vs. Nic + Prop = p value: not significant; Nic vs. Nic + Prop = p value: not significant; Figure 7H ii; Table S1). In Figure S10, it is possible to observe that additional CTR co-cultures replicated the results obtained with the commercial hiPSC line (described above), confirming the responsiveness to drug stimulation and validating the functionality and reliability of the model.

hiPSC-SNs in co-culture abundantly release norepinephrine from varicosities

To correlate the results of functional experiments with the release of norepinephrine, we stained the neurocardiac co-culture with 5 μM

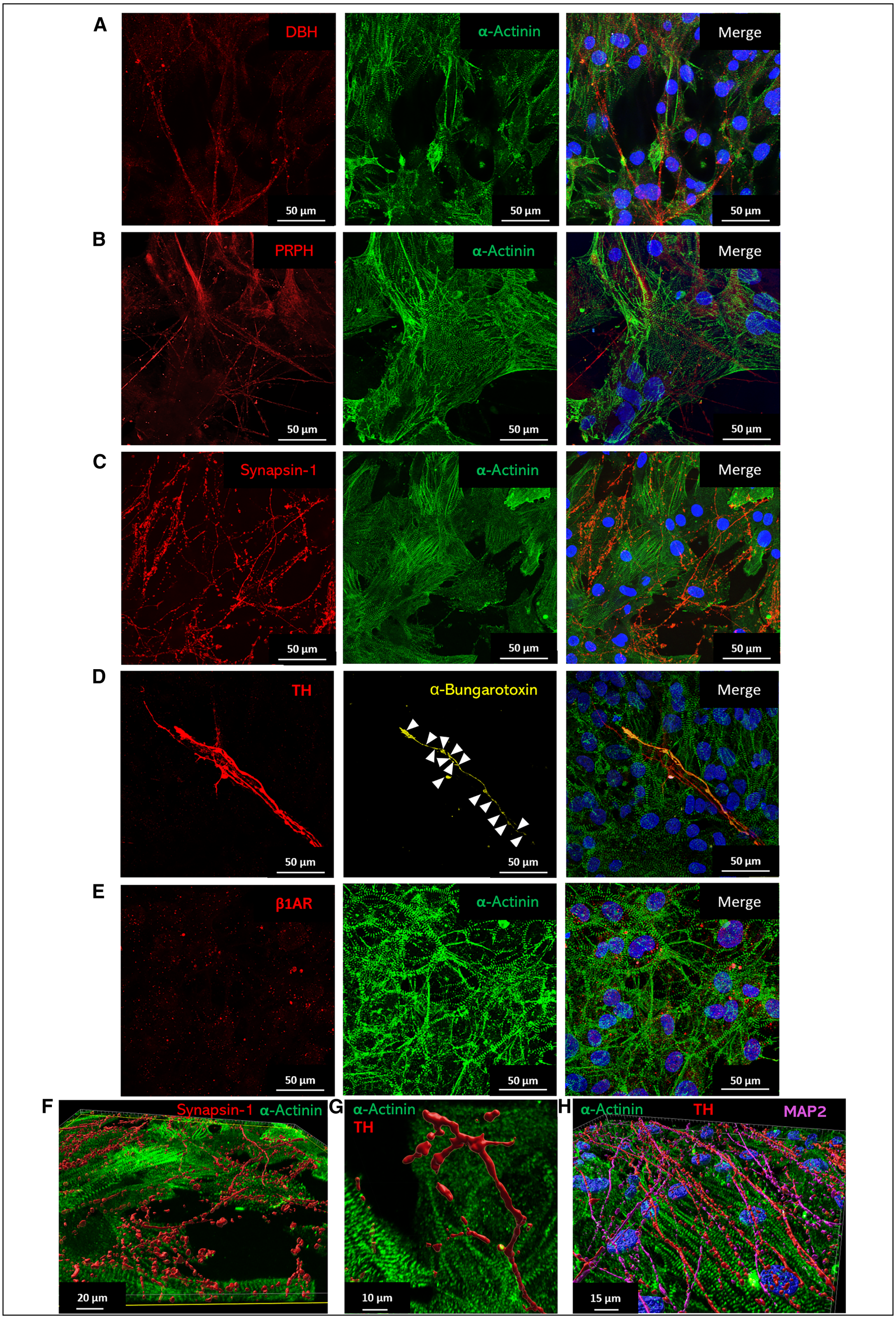

(legend on next page)

fluorescent false neurotransmitter 270 (FFN270), a known substrate for norepinephrine transporter, which labels noradrenergic neurons and allows imaging of synaptic vesicle content release *in vitro*.[44] The release of norepinephrine during exocytosis can be monitored by a decrease in its fluorescent signal (Figure 8). At 7 days of co-culture, hiPSC-SNs were stimulated with 10 μM Nic. A significant decrease in the number of FFN270 puncta was observed 7 min after the Nic administration (not treated vs. Nic = *p* value: 0.0039; Figures 8A and 8B; Table S1), suggesting that the depolarization of hiPSC-SNs induced by Nic, as seen in previous experiments (Figure 7F), causes the release of neurotransmitters that can act on CMs downstream. Of note, no significant decrease in the number of FFN270 dots was observed in the untreated co-cultures, at the same time point (not treated vs. 7 min = *p* value: not significant; Figure 8C; Table S1), indicating that the decrease in FFN270 is specifically caused by the Nic treatment, and it is not a spontaneous degradation of the signal over time. The significant reduction in FFN270 signal was also evaluated and confirmed by performing an analysis of the integrated intensity data, which allowed the quantification of both the signal intensity and the spatial extent of the FFN270 labeling (Figures S11A and S11B; Tables S1 and S2). Figure S11C shows a high magnification image, indicating the reduction in the FFN270 dots after Nic treatment.

DISCUSSION

This study describes the establishment and the functional characterization of a novel neurocardiac co-culture model entirely based on hiPSC-derived cells. Specifically, the proper interaction developed between hiPSC-SNs and hiPSC-CMs can accurately mimic the physiology of the NCJ. After the morphologic, molecular, and electrophysiologic characterization of hiPSC-SNs and hiPSC-CMs and the confirmation of their adrenergic and cardiomyogenic fate, respectively, the two cell populations were co-cultured thanks to a simple two-chamber insert. A few days following its removal, a complex axonal network was formed and was able both to (1) infiltrate the cardiac cell population and (2) respond to different chemical stimuli, modulating in turn CM electrical function.

Growing interest in the CANS can be attributed to its increasingly recognized involvement in various diseases, such as hypertension, heart failure, coronary artery disease, arrhythmias, or neurodegenerative disorders including Parkinson and Huntington disease. However, access to primary human neurons and CMs is highly invasive, while animal models show limited translational relevance due to interspecies differences.[18,23,45,46] Together, these limitations highlight the need for reliable human models that recapitulate the neurocardiac connection. hiPSCs offer a suitable alternative and have been widely used to model several diseases.[47] For instance, different cardiac diseases, like arrhythmogenic cardiomyopathy,[48–51] long QT syndrome,[52–57] or atrial fibrillation,[58,59] alongside neurodegenerative disorders, such as Parkinson disease[60] or familial dysautonomia,[61,62] have been extensively studied using hiPSC-derived cells. While robust and standardized protocols exist for the differentiation of hiPSC-CMs, protocols for SN differentiation remain limited.[7,32,33,63–65] One of the most frequently used protocols is the one originally proposed by Oh et al.[29] and later optimized by Winbo et al.,[7,26] based on activators of the WNT and BMP pathways, which we also used to produce the SN-derived cells in this work. This protocol has allowed us to obtain a high number of hiPSC-SNs derived from two different cell lines, exhibiting the expected adrenergic phenotype, showing positivity for SN markers, and demonstrating the ability to release neurotransmitters. Little adaptations, such as the medium and the well coating, were necessary to optimize the protocol for the hiPSC lines used. In-depth characterization of hiPSC-SNs derived from the two different lines used has demonstrated their effective sympathetic fate, in line with the existing literature.[8,29] The immunofluorescence confirmed the positivity of hiPSC-SNs for the pan-neuronal markers MAP-2 and TUJ, as well as for the sympathetic markers PRPH, TH, and DBH, as previously reported.[26,28,29,33,63] Moreover, presynaptic vesicles stained with synapsin 1 were detected along the axonal processes of the hiPSC-SNs. At day 30 of differentiation, hiPSC-SNs exhibited the characteristic pearl necklace morphology, typical of *en passant* synapses of SNs. The same results were previously described by Zaglia et al. in primary murine SNs and by Bernardin et al. using primary rat SNs.[19,64] The differentiation protocol used in this study yielded a high percentage of TH^+ cells at day 30 of differentiation (between 68.6% and 97.8%), consistent with previous studies, despite the cells being tested at slightly different time points or the employment of alternative differentiation strategies.[28,62] In addition, a high proportion (71%–90%) of TH^+ cells was DBH^+, confirming their adrenergic phenotype. Further validation by western blot experiments confirmed the expression of TH, DBH, and PRPH in hiPSC-SNs at day 30 of differentiation.

Electrical activity of hiPSC-SNs from days 30 to 60 of differentiation was recorded using the MEA platform. Overall, the spontaneous firing activity, burst, and network burst frequency of hiPSC-SNs were spontaneously faint. However, an exception was observed at day 40 of differentiation, when a peak in their electrical activity was observed. This behavior was previously described by Zeltner's group,[61] showing that hiPSC-SNs exhibited a peak in spontaneous activities around day 35.[29,62,63] Slight discrepancies in the final

Figure 6. Immunocytochemical images of co-cultures

Qualitative immunostaining images of co-culture models at time point T7. (A) hiPSC-SNs stained with DBH (red) and hiPSC-CMs stained with α-actinin (green). Scale bars, 20 μm. (B) hiPSC-SNs stained with PRPH (red) and hiPSC-CMs stained with α-actinin (green). Scale bars, 20 μm. (C) hiPSC-SNs stained with synapsin 1 (red) and hiPSC-CMs stained with α-actinin (green). Scale bars, 20 μm. (D) hiPSC-SNs stained with TH (red) and post-synaptic sites stained with α-bungarotoxin (yellow). White arrowheads indicate α-bungarotoxin$^+$ varicosities. Scale bars, 20 μm. (E) hiPSC-CMs stained with β1AR (red) and α-actinin (green). Scale bars, 20 μm. (F) The 3D reconstruction of the co-cultures marked with synapsin 1 (red) and α-actinin (green). Scale bars, 20 μm. (G) The 3D reconstruction of the co-cultures marked with TH (red) and α-actinin (green). Scale bars, 10 μm. (H) The 3D reconstruction of the co-cultures marked with TH (red), MAP2 (purple), and α-actinin (green). Scale bars, 15 μm. Nuclei were counterstained with DAPI (blue).

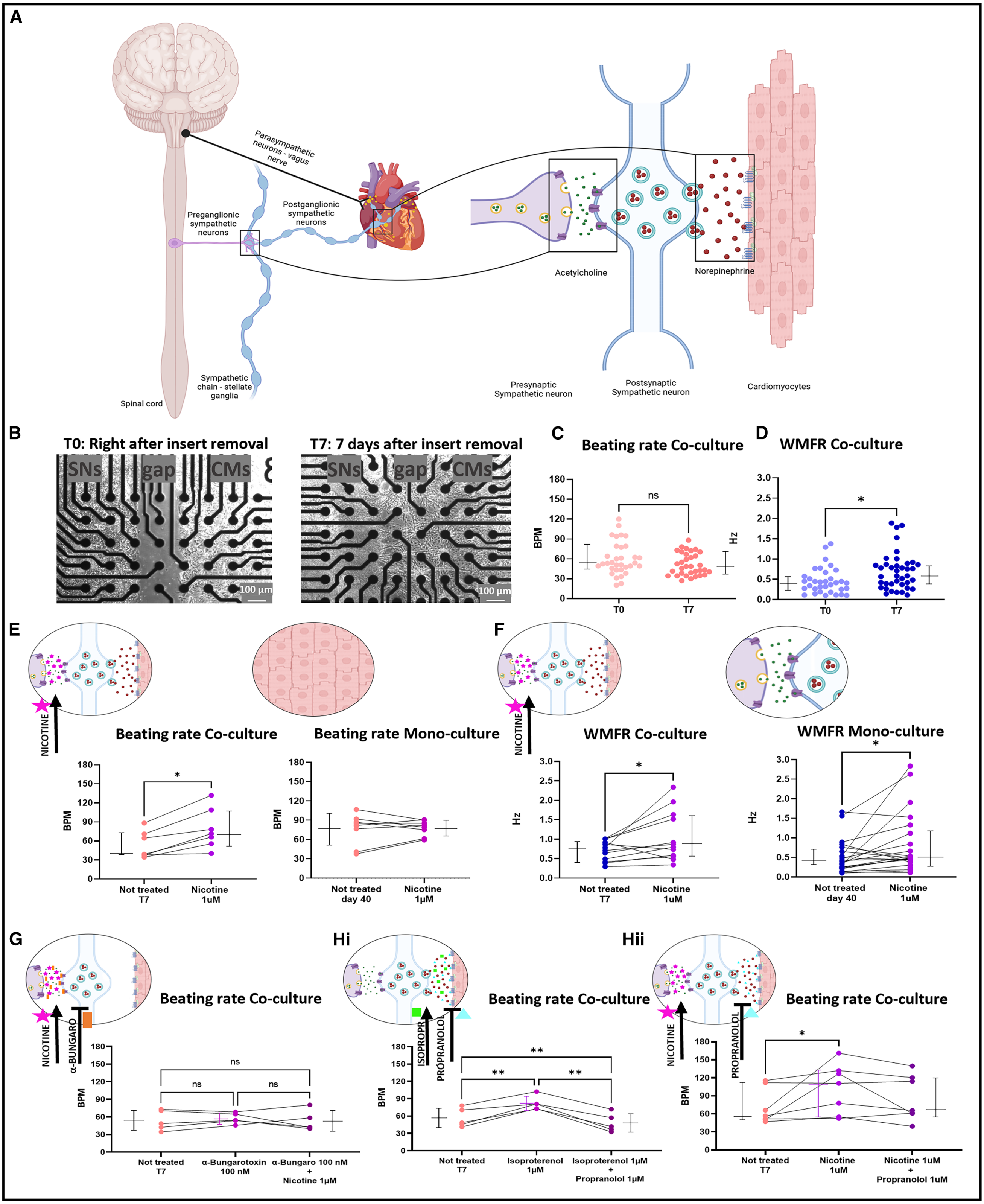

(legend on next page)

MEA measurements may be attributed to variations in the protocol used, in the coating strategy, medium composition (different glucose levels), and, critically, the cell densities used in the MEA plates. These factors have been documented as key determinants of differences in MEA measurements, even in experiments with the same cell line.[65] Furthermore, we hypothesized that the decline in firing activity observed after day 40 can be reconducted to neuronal pruning, a well-established developmental process that selectively eliminates superfluous synaptic connections to refine and stabilize functionally essential neural circuits.[66,67] This hypothesis is supported by the live and dead assay, showing a low level of dead cells at days 50 and 60 compared with day 40, demonstrating that the decrease in firing activity at days 50 and 60 is not caused by cell death but rather by a culture maturation process.

So far, several studies have described the co-culture of SNs and CMs mainly using primary cell populations.[9,20–22,29,64,68–71] In contrast, few studies have attempted to develop a co-culture entirely based on hiPSC-derived cells.[8,26,28,29,43,62,64,72] The most commonly used methods for establishing these types of hiPSC-based co-cultures include either culturing one cell population on top of the other or establishing connectivity through microfluidic chambers that possess tiny micro-tunnels to facilitate the migration of axons.[26–29,72]

In this study, we introduce an innovative, user-friendly, and cost-effective approach to generate a neurocardiac co-culture model, which to our knowledge has not been described before. Using a two-chamber silicone insert, we established a compartmentalized co-culture with separated areas, keeping the two cell populations separated during the initial cell plating and attachment stage. The removal of the insert facilitates the physical connection of the two cell populations, creating an intricate network of elongated axons toward the CMs. The robust interconnection between the two cell types was achieved by optimizing the coating process. A key step of the protocol was to apply Matrigel in the gap between the two cell populations created by the insert. This step supported axonal extension across the gap. Co-culture survival was enhanced by a medium composition capable of maintaining the viability of both cell types, as previously reported.[26] The connection between hiPSC-SNs and hiPSC-CMs was obtained in standard 12- or 24-well plates, without the need for micro-tunnels for the extension of the axons or for a gradient of NGFs in the medium, as observed in other studies using the technology of organ-on-a-chip for the formation of co-cultures.[64] The described protocol can be reproduced on the desired supports (e.g., normal culture plate, imaging supports, MEA plates), simply by applying the silicon insert, without needing any specialized advanced technology. This makes the model highly reproducible and suitable for a wide variety of molecular and functional experiments. However, beyond the establishment of the physical connection between hiPSC-SNs and hiPSC-CMs, the assessment of the functionality of the co-culture is essential to validate the model.

In the co-cultures created with both cell lines, a massive migration of axons was observed toward hiPSC-CMs, and the latter were attracted and re-oriented toward the hiPSC-SNs covering the initial gap between the two cell populations. This behavior was previously reported in other contexts and different diseases,[73] and it is thought to be attributable to signaling molecules present in the medium or paracrine factors released by the cells.[74,75]

The analysis of axon guidance genes revealed a trend of their upregulation in the co-cultures, which may facilitate the axonal attraction toward CMs. Notably, hiPSC-CMs in co-culture exhibited a significant increase in *GDNF* expression compared with hiPSC-CMs in monocultures. These results suggest, in our co-culture model, the higher influence of GDNF than NGF, which was traditionally studied and recognized as the key neurotrophic factor responsible for neuron attraction.[67,71] This observation aligns with previous studies showing that GDNF is even more effective than NGF in promoting axonal outgrowth, while also supporting presynaptic formation.[71]

hiPSC-SNs in co-culture, as in monoculture, possess numerous varicosities distributed along the axons as shown with TH staining. In SNs, the vesicles containing the neurotransmitters are stored in the varicosities, which explains the distribution of pre-synaptic vesicles along

Figure 7. Functional analysis of co-cultures shows that hiPSC-SNs can modulate the beating activity of hiPSC-CMs

(A) Schematic representation of the neurocardiac junction. (B) Bright-field images of co-cultures plated on MEA plates at time points T0 and T7. The electrodes are clearly visible. Scale bars, 100 μm. (C) Spontaneous beating rate of hiPSC-CMs in co-cultures evaluated at T0 (no connections between the two populations) and T7. Median with IQR of eight differentiations with three to six replicates; each dot represents a technical replicate. Test used: Mann-Whitney test, not significant. (D) Spontaneous WMFR of hiPSC-SNs in co-cultures evaluated at T0 (no connections between the two populations) and T7. Median with IQR of 13 differentiations with 1–3 replicates; each dot represents a technical replicate. Test used: Mann-Whitney test; $^*p = 0.01$. (E) Beating rate of hiPSC-CMs in co-cultures at T7 treated with 1 μM Nic vs. not-treated co-culture. Median with IQR of seven differentiations with one replicate. Test used: Wilcoxon test; $^*p = 0.0156$. Beating rate of hiPSC-CMs in monocultures at day 40 treated with 1 μM Nic vs. not-treated co-culture. Mean with SD of four differentiations with two replicates; each dot represents a technical replicate. Test used: Wilcoxon test, not significant. (F) WMFR of hiPSC-SNs in co-culture/monoculture treated with 1 μM Nic vs. not-treated co-culture/monoculture. Co-culture vs. not treated (median with IQR of five co-cultures with one to five replicates; each dot represents a technical replicate). Test used: Wilcoxon test; $^*p = 0.02$. Monoculture vs. not treated (median with IQR of four co-cultures with two to six replicates; each dot represents a technical replicate). Test used: Wilcoxon test; $^*p = 0.021$. (G) Beating rate of hiPSC-CMs in not-treated co-cultures and treated with 100 nM α-bungarotoxin or α-bungarotoxin + Nic. Co-culture vs. not treated (mean with SD of five co-cultures with one replicate). Test used: Tukey multiple comparisons test, not significant. (H) (i) Beating rate of hiPSC-CMs in co-cultures treated with Iso (1 μM) vs. Iso (1 μM) + propranolol (Prop; 1 μM) vs. not treated (mean with SD of five co-cultures with one replicate). Test used: Tukey multiple comparisons test; p value = not-treated co-cultures vs. Iso, $^{**}p = 0.0092$; Iso vs. Iso + Prop, $^{**}p = 0.0017$; not-treated co-cultures. Iso + Prop, $^{**}p = 0.0050$. (H) (ii) Beating rate of hiPSC-CMs in co-cultures treated with 1 μM Nic vs. 1 μM Nic +1 μM Prop vs. not treated (median with IQR of three co-cultures with two to three replicates; each dot represents a technical replicate). Test used: Dunn's multiple comparisons test; $^*p = 0.048$. Schemes created with BioRender.com.

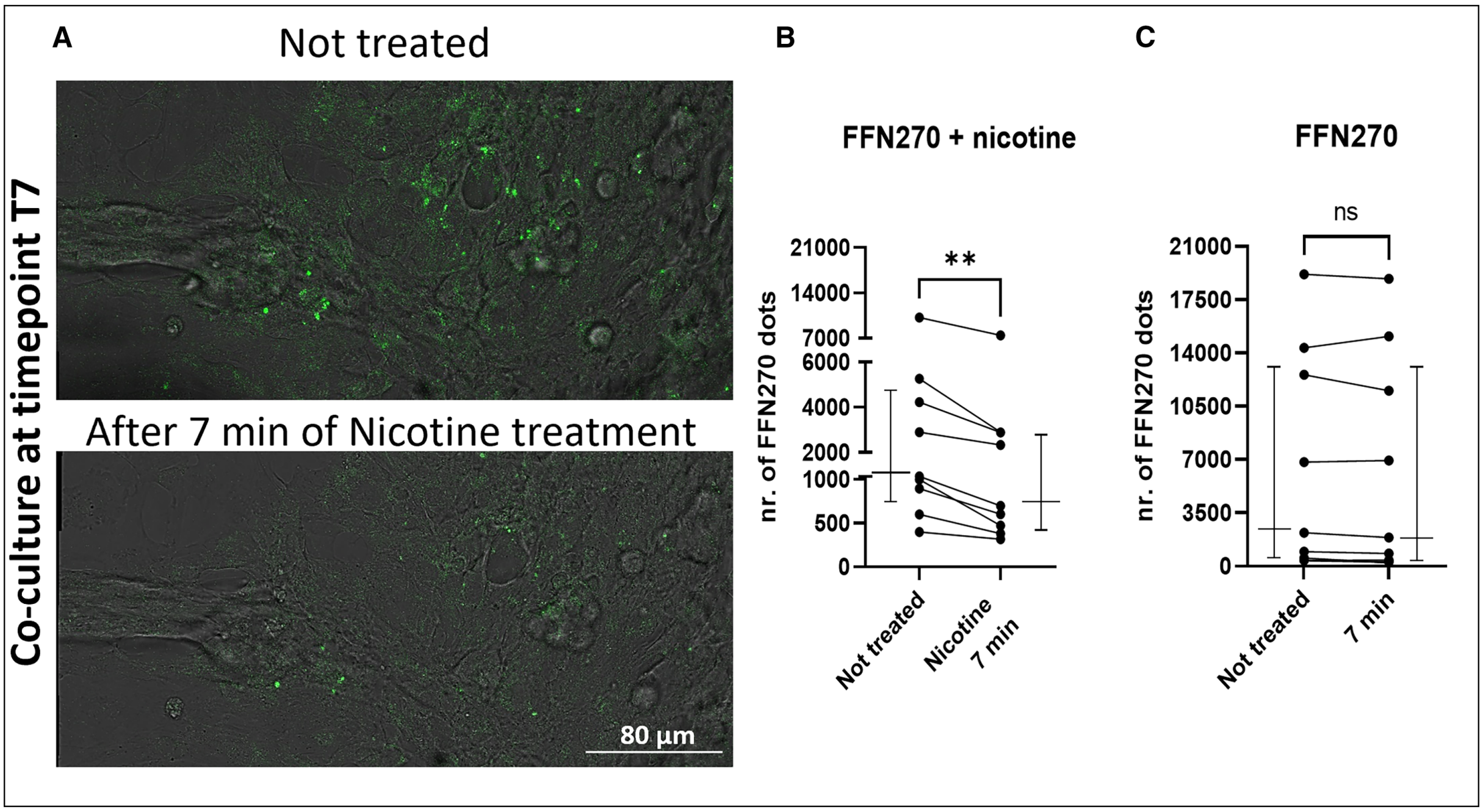

Figure 8. Detection of norepinephrine in co-cultures at T7 released from the varicosities using the false neurotransmitter FFN270

(A) Representative images of co-cultures at T7 not treated and treated with Nic. Scale bar, 80 µm. (B) Co-cultures stained with 5 µM FNN270 and subsequently treated with 10 µM Nic and recorded after 7 min (median with IQR of nine differentiations with one replicate). Test used: Wilcoxon test; $^{**}p = 0.0039$. (C) Representation of control co-cultures treated with 5 µM FNN270 without or without Nic administration and recorded before and after 7 min of incubation (median with IQR of nine differentiations with one replicate). Test used: Wilcoxon test. Each dot represents one co-culture.

of the entire length of the axons.[20,22,64] In addition, nAChRs (marked with α-bungarotoxin) were detected along the varicosities of the axons and colocalized with TH staining, as previously reported by Winbo et al.[26] Pre-synaptic vesicles were found to be uniformly distributed around the cardiac cell, showing a possible interaction between the two cell types.[71] This suggests that there is an accumulation of vesicles potentially ready for exocytosis of hiPSC-SNs around the target cardiac cells. Furthermore, the binding of the neurotransmitter with its specific receptor is ensured by the stable expression of β1AR on the membrane surface of hiPSC-CMs, which we described at day 7 by gene expression and immunofluorescence. This is in line with similar findings for other βARs (e.g., β2AR), as documented by Shcherbakova et al.[21] After 7 days of co-culture, hiPSC-SNs showed increased expression of the key sympathetic genes *TH* and *DBH*. This outcome has been linked to advanced maturity of the hiPSC-SNs.[29]

Previous co-culture studies, as well as the present study, demonstrate that the physical connection between hiPSC-SNs and hiPSC-CMs does not alter the beating rate of CMs.[26,29,43] The increased WMFR of hiPSC-SNs in co-cultures is still not sufficient to modulate the beating rate of hiPSC-CMs. This is a physiological behavior of the cells in baseline conditions, as SNs react only after a precise stimulus or stress.

Under specific stimulation, the sympathetic response triggers the release of noradrenergic neurotransmitters and causes changes in cardiac activity.[67,76] In this work, we successfully reproduced this physiological network by Nic-induced SN activation, which in turn modulated cardiac activity. By separately monitoring both SNs and CMs, MEA results have demonstrated the formation of a functional cellular interaction by showing that under Nic exposure, while neuronal bursts increased both in co-culture and in monoculture, the CMs beating rate accelerated only in the co-culture. In line with previous works, our experiments showed that hiPSC-SNs were successfully stimulated with Nic, thus mimicking the release of acetylcholine-induced noradrenaline. The functional integrity of the intercellular signals from the pre-synaptic to the post-synaptic space up to the NCJ is proved by the fact that the increase of the beating rate of the CMs is not only prevented by α-bungarotoxin, a well-known nicotinic-specific receptor blocker,[26] but also most crucially by the displacement of the neurotransmitter in the presence of Prop, a non-selective β-adrenergic agonist,[20,27] during the Nic stimulation. These results demonstrated that Nic-stimulated hiPSC-SNs have a modulatory effect on the cardiac beating rate, thereby supporting the ability of hiPSC-SNs to form functional and active connections with hiPSC-CMs and, therefore, to modulate their electrical activity. The ability of SNs to modulate cardiac activity was further validated by establishing co-cultures using a second iPSC line. These latter co-cultures exhibited the same functional electrophysiological behavior, morphologic, and molecular profile described here above, indicating that the structural connection and regulatory influence of SNs on the cardiac beating rate can be readily

achieved and reproduced with this removable insert-based method. Such consistency underscores the robustness of the model and its accuracy in replicating NCJ functionalities.

Furthermore, the observation of the exocytosis process, with FFN270 at the varicosities level, demonstrates the ability of vesicles to release neurotransmitters upon a specific stimulus (Nic stimulation). The same false fluorescent neurotransmitters method was previously successfully used in living rodents,[44] and the experiments of our current work further demonstrate that it is easily applicable not only *in vivo* but also *in vitro*.

In conclusion, we successfully generated a novel, reproducible, and cost-efficient neurocardiac human model, based entirely on hiPSC-derived cells. We are the first in generating a basic human co-culture model without mixing the two cell populationns or plating them one on the top of the other, thanks to the use of a small commercially available insert. Because of the conformation of the model, with the two distinct populations well separated, it is possible to extract information from both single populations at the same time but also obtaining clear data and a deep analysis of the two connected population, as demonstrated for examples by MEA measurements. Additionally, it should be noted that our model is created without the need of any elaborate technological engineering. The fact that few ad hoc organ-on-a-chip or microfluidic devices have been developed for neuro-cardiac studies should not be underestimated. Researchers must customize their devices, making the process long and technically complicated, inevitably leading to results that are not always reproducible.[77] These considerations highlight the importance of reliable and accessible methodology to generate this type of model, such as the one presented in this study. Furthermore, this model shows high responsiveness to pharmacologic stimulation, making it an optimal tool for drug screening studies. It allows researchers to evaluate the effects of compounds not only on the targeted cell population but also on the related interconnected population. This can be very helpful in the evaluation of the cardiac safety and cardiotoxicity of non-cardiac drugs. This two-dimensional (2D) co-culture model can be viewed as a proof of concept of a basic non-animal alternative method, as it captures the essential principles of human neuro-cardiac communication in a simplified compartmentalized setup. Finally, it can also be used to study the pathophysiological mechanisms of cardiac diseases involving autonomic innervation, such as arrhythmogenic cardiomyopathy or catecholaminergic polymorphic ventricular tachyarrhythmia, as well as to investigate the negative implications of neurodegenerative diseases on heart function, including Parkinson disease.

Limitations of the study

Although there are multiple advantages to using hiPSC-derived cells, one disadvantage is the immaturity of the cells differentiated from hiPSCs. hiPSC-CMs resemble fetal CMs, appearing similar to 16-week fetal CM than adult CMs.[78–81] As revealed by the expression analysis of ion channel genes, which affect the electrophysiological response of the cells, hiPSC-CMs in co-culture do not show changes in the expression levels compared with hiPSC-CMs in monoculture. This can be attributed to the still-immature phenotype of hiPSC-CMs, not improved even by the presence of SNs after 7 days of co-culture. This issue can be observed in hiPSC-SNs as well. Indeed, no protocol for the differentiation of hiPSC-SNs re-encounters a level of maturity comparable to that of primary neurons.[29,31,33,61,82] Although this point is not the focus of this work, many strategies can be employed to try to ameliorate the maturity of the cells, including the co-culture methods, as reported by Oh's group.[29] It is believed that the immature phenotype of hiPSC-SNs can cause a reduced release of neurotransmitters.[29] In this work, we investigated the ability of vesicles to release norepinephrine, but a more detailed analysis of neurotransmitter activity is required for a more comprehensive knowledge. The results described in this work were obtained after 7 days of co-culture, because at this point certain differences were already evident. However, we can hypothesize that a longer co-culture timeline may be useful for improving the maturity of hiPSC-CMs and hiPSC-SNs. Unlike purified hiPSC-CMs, the hiPSC-SNs used in this work have not undergone a purification step that separates them from non-neuronal cells. As a result, the population used is not exclusively composed of hiPSC-SNs, which may attenuate the effects observed in the results. Finally, one last consideration can be made about the model itself. In the body, cardiac autonomic innervation comprises the sympathetic and the parasympathetic branches. Therefore, the model could be implemented with a third population, namely hiPSC-parasympathetic neurons, to better mimic the *in vivo* human heart environment.

Conclusion

To the best of our knowledge, this is the first compartmentalized NCJ model based exclusively on human iPSC-derived cells with a two-chamber silicon insert, allowing an easy and accessible laboratory implementation. The effectiveness of the present model was demonstrated by the structural and functional crosstalk between hiPSC-CMs and hiPSC-SNs. Indeed, our data showed that only activated hiPSC-SNs are able to release neurotransmitters from the varicosities and to influence the beating rate of hiPSC-CMs, demonstrating that the cardiac activity is strictly dependent on actively firing hiPSC-SNs and not only on their presence.

MATERIALS AND METHODS

Human iPSC culture and passaging

The commercially available cell line human episomal iPSC line (Gibco, catalog no. A18945) was used in the present study. Cells were cultured following the manufacturer's instructions. Briefly, they were grown on Matrigel-coated (Corning) (0.083 mg/mL) plates with Essential 8 Medium (Thermo Fisher Scientific) and split in colony with EDTA (Gibco) in PBS (Biowest) (0.5 mmol). Cells were regularly tested for karyotype normality and mycoplasma presence (Lonza).

A second healthy CTR hiPSCs line (EURACi009-A; https://hpscreg.eu/cell-line/EURACi002-A), available in the laboratory, was cultured as described by De Bortoli et al.[40] Cell line generation

and characterization details are available in Meraviglia et al.[41] The present study complied with the principles outlined in the Declaration of Helsinki and it was approved by the ethical committee of the Province of Alto Adige/South Tyrol (No. 1/2014, March 12, 2014). Blood was collected after written informed consent was obtained.

Human iPSC differentiation into CMs

Both hiPSCs lines were plated at a density of 5,730 cells/cm^2 in a previously prepared Matrigel-coated plate (0.083 mg/mL) with fresh Essential 8 Medium (Thermo Fisher Scientific) supplemented with 10 μM rho-associated protein kinase inhibitor Y-27632 (Y) (Biomed Reagent). Medium was changed daily. After 3 days from the passaging, the protocol for the cardiomyogenic differentiation was initiated. Cardiomyogenic differentiation was performed as described in De Bortoli et al.[40] After the purification, cells were plated at a density of 1.43 $\times$ 10^5 cells/cm^2 on a pre-coated 12-well plate with Matrigel (0.083 mg/mL) in CM basal medium (RPMI 1640 with L-Glu, Biowest; 1% penicillin/streptomycin [P/S], Biowest; and 1$\times$ B-27 supplement, Gibco) supplemented with 10 μM Y.

Human iPSC differentiation into SNs

The differentiation strategy follows the protocol published by Oh et al. with minor modifications.[26,29] Briefly, both hiPSCs lines were plated as single cells at the density of 5.7 $\times$ 10^4/cm^2 in a 12-well plate coated with 0.5 mg/mL Matrigel in Essential 8 Medium supplemented with 10 μM Y. Medium was changed daily without Y until the confluence of 95% was reached. For the beginning of the differentiation on day 1, KSR medium (KnockOut DMEM, Gibco; 15% KnockOut serum, Gibco; 2 mM L-glutamine, Biowest; 1$\times$ P/S, Biowest; 1$\times$ non-essential amino acids, Biowest; 0.01 mM β-mercaptoethanol, Gibco) was used. From day 5, a gradual change from KSR medium to NBB27 medium was performed. NBB27 medium is composed of neurobasal medium (Gibco), 0.5$\times$ B-27 supplement (50$\times$, Gibco), and 1$\times$ P/S, Biowest. From days 1 to 16, a different combination of factors was added to the media (a schematic representation of the protocol is found in Tables S3 and S4. The sorting procedure at day 14 of the original protocol was substituted by a manual passaging of the cells on days 12–13 of differentiation. Cells were passaged *en bloc* to be maintained for a prolonged time in culture on 24-well plates precoated with 0.06 mg/mL poly-D-lysin (Sigma-Aldrich) and 10 μg/mL laminin (Sigma-Aldrich). On the day of the manual passaging, cells were supplemented with 10 μM Y. Further maturation of cells toward sympathetic lineage was reached with an additional 15 days in culture (from days 16 to 30 and onward) in complete NBB27 medium supplemented with the factors listed in Tables S3 and S4.

Setup of neurocardiac co-culture composed of hiPSC-SNs and hiPSC-CMs

A silicon insert (Culture-Insert 2 Well by Ibidi, catalog no. 80209-150; growth area per well, 0.22 cm^2; gap, 500 μm) composed of two attached and divided rectangular compartments/chambers was added into the wells of a 12-well plate or μ-Slide 4 well ibiTreat (Ibidi, catalog no. 80426). hiPSC-SNs, produced as described above, were detached at day 20 by treatment with Stem Pro Accutase (Gibco) for 6 min at 37°C; the cell suspension was then collected in a falcon tube and counted with the LUNA FL Fluorescence Cell Counter (Logos Biosystems). hiPSC-SNs were then plated into one of the chambers of the silicon insert and coated with 0.5 mg/mL Matrigel. Concomitantly, hiPSC-derived CMs were purified at days 18–20 and plated in the second chamber of the insert, which was coated with 0.083 mg/mL Matrigel (40 min incubation time at 37°C). Both protocols for the neural and cardiomyogenic differentiations were started on the same day. The cell number plated in each chamber was 9 $\times$ 10^4 cells for both cell populations, using their basal medium supplemented with 10 μM Y. The second day after the plating, individual media were changed without the addition of Y, and the two cell types were kept separate until the removal of the inserts. The insert was removed when hiPSC-SNs reached day 30 of the differentiation. The inserts were slowly removed with sterile tweezers, and, to coat the gap between the two cell populations, an additional drop of 0.083 mg/mL Matrigel was added on top of the two cell populations and incubated for 30 min at 37°C. After the incubation, the cells were maintained in culture for 7 days using a co-culture medium composed 1:1 by CM basal medium and complete NBB27 medium and changed every other day.

Neuroblastoma SH-SY5Y cell culture and passaging

Human neuroblastoma SH-SY5Y cells (American Type Culture Collection, stock no. CRL-2266) were cultured as previously described.[83] Briefly, SH-SY5Y cells were cultured in DMEM-F12 (Biowest) supplemented with 10% fetal bovine serum (FBS) (Sigma-Aldrich, catalog no. F2442) and 1% P/S. At 70%–80% confluence, SH-SY5Y cells were split with trypsin-EDTA (Gibco) for 5 min at 37°C.

Flow cytometry analysis

At day 30 of differentiation, hiPSC-SNs in monoculture were analyzed with flow cytometry. We detached 1 $\times$ 10^6 cells per condition with StemPro Accutase and collected in 1.5-mL tubes. After centrifugation at 200 $\times$ g for 5 min, supernatant was discarded, and the cells were washed once with sterile fluorescence-activated cell sorting (FACS) buffer (PBS; 0.5% FBS and 2 mM EDTA) and then resuspended in 4% paraformaldehyde (PFA) and incubated for 15 min at room temperature (RT). Cells were washed with sterile FACS buffer and centrifuged at 200 $\times$ g for 5 min. Subsequently, cells were permeabilized for 15 min with permeabilization solution (0.1% Triton X-100 [Sigma-Aldrich] in PBS), and after this step, cells were washed with FACS buffer and centrifuged at 200 $\times$ g for 5 min. Cells were stained with the conjugated antibodies against TH-phycoerythrin and against DBH-fluorescein isothiocyanate (see Table S5), resuspended in permeabilization solution for 30 min at RT. Following the incubation step, cells were washed with permeabilization solution and centrifuged at 200 $\times$ g for 5 min. The pellet was resuspended in FACS buffer and filtered with a 50- to 70-μM cell strainer in FACS tubes. Stained cells were run on an S3e Cell Sorter (Bio-Rad). The fluorescence intensity of 30,000 events was quantified and analyzed with

FlowJo software version 10.8.1. Fluorescence intensity was determined based on the gating of hiPSC-SNs population for TH positivity, and on this subpopulation, the DBH positivity was determined.

Immunofluorescence analysis

Cells were fixed with 4% PFA for 15 min and then permeabilized with 0.5% Triton X-100 in PBS for 10 min at RT. Cells were blocked in 5% goat serum (Invitrogen) in PBS for 1 h at RT and incubated with primary antibodies in blocking solution overnight (ON) at 4°C. The next day, samples were washed three times with PBS and incubated with the proper secondary antibody in blocking solution (2% goat serum in PBS) for 1 h at 37°C. Samples were washed three times with PBS and then incubated with DAPI (Invitrogen). The images, with a resolution of 3,504 × 3,504 pixels for CMs and 2,048 × 2,048 pixels for SNs and co-cultures, were acquired using a Leica SP8-X confocal microscope with a 63× oil immersion objective and Leica LAS-X software (Leica Microsystems). Reconstruction of areas of interest were made from 2D projections of confocal stacks. The 3D reconstructions were made using Imaris Software (Oxford Instruments). All antibodies are listed in Table S5.

Western blot analysis

Cell pellets were lysed with radioimmunoprecipitation assay buffer (Thermo Fisher Scientific), supplemented with protease inhibitors (complete tablets, Mini EASYpack, Roche) and phosphatase inhibitors (complete tablets, Mini EASYpack, Roche). The quantification of proteins was performed using the Pierce bicinchoninic acid protein assay kit (Thermo Scientific) following the manufacturer's instructions. Denatured protein lysates were loaded on precast polyacrylamide gel NuPAGE 4%–12% Bis-Tris gels (Thermo Fisher Scientific) for the electrophoretic separation and then transferred on polyvinylidene difluoride membranes using Novex Mini Cell (Invitrogen)/Criterion Blotter (Bio-Rad). The detection of the proteins was performed using the Clarity ECL Western Substrate Kit (Bio-Rad) and the ChemiDoc Touch Imaging System instrument (Bio-Rad). All antibodies are listed in Table S5.

Gene expression analysis

Cell pellets were resuspended in 1 mL TRIzol Reagent (Thermo Fisher Scientific). RNA extraction was performed using the Direct-zolRNA MiniPrep Kit (Zymo Research), and retrotranscription (1,000 ng) was performed using the SuperScript VILO cDNA Synthesis Kit (Thermo Fisher Scientific) following the manufacturer's instructions. Quantitative real-time PCR was performed with TaqMan Assay (Applied Biosystems-Thermo Fisher Scientific), with a reaction of 20 μL containing TaqMan Fast Advanced Master Mix 2× (Applied Biosystems), TaqMan Assay probe 20× (Applied Biosystems), 12.5 ng cDNA, and nuclease-free water to reach the final volume. The amplification reaction was performed using the CFx96 Real-Time System C1000 Thermal Cycler (Bio-Rad) repeating the following program for 40 cycles: 2 min at 50°C, 95°C for 20 s, 95°C for 3 s, and 60°C for 30 s. As normalizers, housekeeping genes were identified with NormFinder and Bio-Rad CFX Maestro software. The amount of the gene of interest for the monocultures of hiPSC-CMs was calculated in relation to the housekeeping gene and reference sample (hiPSCs) using the formula $2^{-\Delta\Delta Ct}$. Gene expression levels of co-cultures were calculated with the reference sample monoculture of hiPSC-CMs at day 30 when analyzing cardiac genes and monoculture of hiPSC-SNs at day 30 when analyzing neural genes using the formula $2^{-\Delta\Delta Ct}$. The *GAPDH* gene was used for the normalization step, except for ion channels and axon guidance genes analysis. In this case, *TNNI3* gene was used, since the analysis was specifically focused on CMs. The TaqMan probes are listed in Table S6.

Preparation of hiPSC-CMs and hiPSC-SNs monocultures and co-cultures for MEA analysis

Monocultures

The 24 wells of the MEA plate (CytoView MEA 24-White, Axion Biosystems) were coated with Matrigel specifically for hiPSC-SNs and for hiPSC-CMs, as described above, and incubated for 40 min at 37°C. Deionized sterile water was added in the outer rim of the plate to avoid evaporation. Matrigel was removed by aspiration just before plating the cells without any washing step, and the plate was left open for 5 min under the hood to slightly dry the wells. hiPSC-SNs were detached at days 12–13 (replating day) and hiPSC-CMs at days 17–18 (purification day), respectively, with Accutase and 0.05% trypsin-EDTA, centrifuged at 200 × *g* for 5 min and plated on the MEA plate at a density of 9×10^4 cells for both cell types. The hiPSC-SNs were resuspended in complete NBB27 medium and hiPSC-CMs in CM basal medium, both supplemented with 10 μM Y. In each well of the MEA plate, 7 μL cell suspension was plated dropwise and incubated for 1 h at 37°C. Lastly, the medium was slowly added to each well two times, up to 500 μL, and changed every 2 days. The plates were recorded with the Maestro Edge system by Axion Biosystems and the software AxIS Navigator applying the neural module for hiPSC-SNs and the cardiac module for hiPSC-CMs.

Co-cultures

The co-cultures were created as mentioned above. For this experiment, they were induced on a 6-well MEA plate (CytoView MEA 6-White, Axion Biosystems). After the removal of the insert, the cells were kept in the co-culture medium and changed every 2 days. The plates were recorded with Maestro Edge system by Axion Biosystems using the software AxIS Navigator, and by applying one module at a time, the neural module for SNs and the cardiac module for CMs.

Acquisition of MEA data and analysis

Data were generated using the Axion Maestro Edge MEA system and acquired with AxIS Navigator version 3.7.1. The instrument provided a constant temperature of 37°C and a CO_2 concentration of 5%. The plates in use were of two types: the 6-well MEA plate with 64 electrodes (grid 8 × 8) (CytoView MEA 6-White, Axion Biosystems) and the 24-well MEA plate with 16 electrodes (grid 4 × 4) (CytoView MEA 24-White, Axion Biosystems). Before each measurement, each plate was equilibrated in the instrument for 5–10 min.

Neural recording

MEA recordings with AxIS software used a sampling frequency of 12.5 kHz per well with pass filters of 200 Hz–3 kHz. For the recording of neural activity, the "neural configuration" was used. Baseline measurements were recorded for 10 min. The obtained RAW files were reopened with AxIS software, the neural configuration was applied again, and a threshold of 5.5 times the background noise was applied for the detection of spikes. Spikes profile data were subsequently exported to a Microsoft Excel file. A number of relevant parameters were taken into account, as listed below. The minimum spike rate to consider an electrode active was 5 spikes per minute. Spontaneous activity refers to the parameter WMFR, corresponding to the spontaneous firing normalized for the number of active electrodes. One burst was detected with a minimum number of 5 spikes with an inter-spike interval of 100 ms. The 35% of electrodes in a well should participate in the network burst detection having a minimum number of 50 spikes, with an inter-spike interval of 100 ms. The synchrony index was evaluated with a synchrony window of 20 ms.

CMs recording

MEA recordings with AxIS software used a sampling frequency of 12.5 kHz acquiring field potential data with a bandwidth of 0.1 Hz–2 kHz. For the recording of cardiac activity, the "cardiac configuration" was used. Field potential activity was recorded for 2 min after at least 10 min of signal stabilization. The obtained RAW files were reopened with AxIS software, the cardiac configuration was applied again, and a threshold was applied. Cardiac profile data were provided by the Excel file after setting a threshold between 100 and 350 μV the background noise.

Co-culture recording

MEA recordings were made by measuring one population at a time and using its specific registration module (neural and cardiac modules). During the measurements, no electrodes were deselected from the recording, and all wells were entirely measured. Recordings of the two cell populations were selected, exported, and analyzed separately.

Imaging FFN270 transients

Co-cultures were prepared on a μ-Slide 4 well ibiTreat (Ibidi, catalog no. 80426) slides as described. After 7 days from the removal of the insert (time point T7), 5 μM FFN270 (excited at 405 nm, emission maximum: 475 nm) (Tocris, catalog no. 6717) were added to the culture medium for 30 min at 37°C.

After the incubation, three washes with Hank's balanced salt solution (Gibco) were performed at RT. Images were acquired with the Leica SP8-X confocal microscope with Leica LAS-X software (Leica Microsystems) using the resonant (fast) scanning mode and a 63× oil immersion objective. The region of interest (ROI) was selected using the LAS X Navigator module, creating an area of about 100 fields. The same ROI was acquired before and after the addition of Nic (10 μM, 6 min of incubation). Living cells were maintained at controlled temperature, CO_2, and humidity as reported in the following paragraph. Images were analyzed using ImageJ counting the number of dots or analyzing the integrated intensity.

Live-cell imaging

Live-cell time-lapse imaging was performed on co-cultures of hiPSC-CMs and hiPSC-SNs, using the environmental chamber on the confocal system Leica SP8-X confocal microscope with Leica LAS-X software to maintain temperature, CO_2, and humidity levels. The Leica LAS X software was set to perform 100 stacks ON, at intervals of 5 min, at a resolution of 1,024 × 1,024 pixels.

Cell viability assay

Cell viability was assessed by live and dead assay using ReadyProbes Cell Viability Imaging Kit (Blue/Green) (Invitrogen, catalog no. R37609) where blue (NucBlue live reagent, Hoechst, catalog no. 33342) indicates live cells and green (NucGreen dead reagent) indicates dead cells. Cells were stained for 15 min at 37°C. The staining was removed, and images were acquired using the Leica SP8-X confocal microscope with a 10× objective and Leica LAS-X software (Leica Microsystems).

Statistical analyses

Statistical analyses were performed using GraphPad Prism 9. To analyze the differences between two experimental groups, datasets were first tested for normality; if normally distributed, then a parametric paired *t* test was used. Otherwise, datasets were analyzed with the non-parametric Wilcoxon matched-pairs signed rank test or Mann-Whitney test. One-way ANOVA was used in experiments comparing three or more groups, followed by Tukey's multiple comparisons test, the Holm-Šídák's multiple comparisons test, or the Kruskal-Wallis test. The threshold for significance was set at $p < 0.05$. Median values and interquartile ranges were calculated (except when indicated) and reported in Tables S1 and S2.

DATA AND CODE AVAILABILITY

All data supporting the findings of this study, including raw experimental data and details on statistical analyses and methodology, are available in Tables S1–S6.

ACKNOWLEDGMENTS

The authors thank the Department of Innovation, Research University and Museums of the Autonomous Province of Bozen/Bolzano for covering the Open Access publication costs.This study was supported by the projects INCardio (ITAT1047 grant) funded by the European Regional Development Fund and Interreg V-A Italy-Austria 2014–2020, and PROMOS (ITAT-11-023 grant) funded by the European Regional Development Fund and Interreg VI-A Italy-Austria 2021–2027. The study was also supported by PRIN_202249XEA5 from the Ministero Dell'Universtà e della Ricerca, to M.M. The authors also acknowledge the contribution of Elena Selciato for English-language editing.

AUTHOR CONTRIBUTIONS

G.C. performed the conceptualization, investigation, methodology, data curation, and writing – original draft. C.A. performed supervision, data curation, and writing – review & editing. G.G., L.S.F., P.G., E.P., and A.G.G. performed the investigation. A.A. L., C.V., and L.F. performed the data curation. M.M., L.P., and L.B. performed writing – review & editing. P.P.P. performed supervision. S.Z. performed writing – review & editing and funding acquisition. I.P. performed supervision and writing – review & editing. A.Z. performed supervision, data curation, methodology, and writing – review & editing. A.R. and M.D. performed supervision, data curation, methodology, writing – review & editing, and funding acquisition.

DECLARATION OF INTERESTS

The authors declare no competing interests.

SUPPLEMENTAL INFORMATION

Supplemental information can be found online at https://doi.org/10.1016/j.ymthe.2026.03.034.

REFERENCES

1. Armour, J.A. (2008). Potential clinical relevance of the 'little brain' on the mammalian heart. Exp. Physiol. *93*, 165–176. https://doi.org/10.1113/expphysiol.2007.041178.
2. Vanhoutte, P.M., and Levy, M.N. (1980). Prejunctional cholinergic modulation of adrenergic neurotransmission in the cardiovascular system. Am. J. Physiol. *238*, H275–H281. https://doi.org/10.1152/ajpheart.1980.238.3.H275.
3. Levy, M.N. (1990). Autonomic interactions in cardiac control. Ann. N. Y. Acad. Sci. *601*, 209–221. https://doi.org/10.1111/j.1749-6632.1990.tb37302.x.
4. Choate, J.K., Klemm, M., and Hirst, G.D. (1993). Sympathetic and parasympathetic neuromuscular junctions in the guinea-pig sino-atrial node. J. Auton. Nerv. Syst. *44*, 1–15. https://doi.org/10.1016/0165-1838(93)90374-4.
5. Hamill, R.W., and Shapiro, R.E. (2004). 5 - Peripheral Autonomic Nervous System. In Primer on the Autonomic Nervous System, Second Edition, D. Robertson, I. Biaggioni, G. Burnstock, and P.A. Low, eds. (Academic Press), pp. 20–28. https://doi.org/10.1016/B978-012589762-4/50006-2.
6. VanPatten, S., and Al-Abed, Y. (2017). The challenges of modulating the 'rest and digest' system: acetylcholine receptors as drug targets. Drug Discov. Today *22*, 97–104. https://doi.org/10.1016/j.drudis.2016.09.011.
7. Habecker, B.A., Bers, D.M., Birren, S.J., Chang, R., Herring, N., Kay, M.W., Li, D., Mendelowitz, D., Mongillo, M., Montgomery, J.M., et al. (2025). Molecular and cellular neurocardiology in heart disease. J. Physiol. *603*, 1689–1728. https://doi.org/10.1113/jp284739.
8. Wu, H.F., Hamilton, C., Porritt, H., Winbo, A., and Zeltner, N. (2025). Modelling neurocardiac physiology and diseases using human pluripotent stem cells: current progress and future prospects. J. Physiol. *603*, 1865–1885. https://doi.org/10.1113/jp286416.
9. Pianca, N., Di Bona, A., Lazzeri, E., Costantini, I., Franzoso, M., Prando, V., Armani, A., Rizzo, S., Fedrigo, M., Angelini, A., et al. (2019). Cardiac sympathetic innervation network shapes the myocardium by locally controlling cardiomyocyte size through the cellular proteolytic machinery. J. Physiol. *597*, 3639–3656. https://doi.org/10.1113/jp276200.
10. Jamali, H.K., Waqar, F., and Gerson, M.C. (2017). Cardiac autonomic innervation. J. Nucl. Cardiol. *24*, 1558–1570. https://doi.org/10.1007/s12350-016-0725-7.
11. Fedele, L., and Brand, T. (2020). The Intrinsic Cardiac Nervous System and Its Role in Cardiac Pacemaking and Conduction. J. Cardiovasc. Dev. Dis. *7*, 54. https://doi.org/10.3390/jcdd7040054.
12. Drew, R.C., and Sinoway, L.I. (2012). Chapter 36 - Autonomic Control of the Heart. In Primer on the Autonomic Nervous System, Third Edition, D. Robertson, I. Biaggioni, G. Burnstock, P.A. Low, and J.F.R. Paton, eds. (Academic Press), pp. 177–180. https://doi.org/10.1016/B978-0-12-386525-0.00036-6.
13. Zaccolo, M., and Pozzan, T. (2002). Discrete microdomains with high concentration of cAMP in stimulated rat neonatal cardiac myocytes. Science (New York, N.Y.) *295*, 1711–1715. https://doi.org/10.1126/science.1069982.
14. Rochais, F., Vandecasteele, G., Lefebvre, F., Lugnier, C., Lum, H., Mazet, J.L., Cooper, D.M.F., and Fischmeister, R. (2004). Negative feedback exerted by cAMP-dependent protein kinase and cAMP phosphodiesterase on subsarcolemmal cAMP signals in intact cardiac myocytes: an in vivo study using adenovirus-mediated expression of CNG channels. J. Biol. Chem. *279*, 52095–52105. https://doi.org/10.1074/jbc.M405697200.
15. Burton, R.B., Tomek, J., Ambrosi, C.M., Larsen, H.E., Sharkey, A.R., Capel, R.A., Corbett, A.D., Bilton, S., Klimas, A., Stephens, G., et al. (2020). Optical interrogation of sympathetic neuronal effects on macroscopic cardiomyocyte network dynamics. iScience *23*, 101334. https://doi.org/10.1016/j.isci.2020.101334.
16. DiFrancesco, D., and Borer, J.S. (2007). The funny current: cellular basis for the control of heart rate. Drugs *67*, 15–24. https://doi.org/10.2165/00003495-200767002-00003.
17. Shen, M.J., and Zipes, D.P. (2014). Role of the autonomic nervous system in modulating cardiac arrhythmias. Circ. Res. *114*, 1004–1021. https://doi.org/10.1161/CIRCRESAHA.113.302549.
18. Hanna, P., Rajendran, P.S., Ajijola, O.A., Vaseghi, M., Andrew Armour, J., Ardell, J. L., and Shivkumar, K. (2017). Cardiac neuroanatomy - Imaging nerves to define functional control. Auton. Neurosci. *207*, 48–58. https://doi.org/10.1016/j.autneu.2017.07.008.
19. Zaglia, T., and Mongillo, M. (2017). Cardiac sympathetic innervation, from a different point of (re)view. J. Physiol. *595*, 3919–3930. https://doi.org/10.1113/JP273120.
20. Prando, V., Da Broi, F., Franzoso, M., Plazzo, A.P., Pianca, N., Francolini, M., Basso, C., Kay, M.W., Zaglia, T., and Mongillo, M. (2018). Dynamics of neuroeffector coupling at cardiac sympathetic synapses. J. Physiol. *596*, 2055–2075. https://doi.org/10.1113/jp275693.
21. Shcherbakova, O.G., Hurt, C.M., Xiang, Y., Dell'Acqua, M.L., Zhang, Q., Tsien, R. W., and Kobilka, B.K. (2007). Organization of beta-adrenoceptor signaling compartments by sympathetic innervation of cardiac myocytes. J. Cel Biol. *176*, 521–533. https://doi.org/10.1083/jcb.200604167.
22. Dokshokova, L., Franzoso, M., Di Bona, A., Moro, N., Sanchez Alonso, J.L., Prando, V., Sandre, M., Basso, C., Faggian, G., Abriel, H., et al. (2022). Nerve growth factor transfer from cardiomyocytes to innervating sympathetic neurons activates TrkA receptors at the neuro-cardiac junction. J. Physiol. *600*, 2853–2875. https://doi.org/10.1113/jp282828.
23. Ohnuki, M., and Takahashi, K. (2015). Present and future challenges of induced pluripotent stem cells. Philos. Trans. R. Soc. Lond. B Biol. Sci. *370*, 20140367. https://doi.org/10.1098/rstb.2014.0367.
24. Takahashi, K., and Yamanaka, S. (2006). Induction of pluripotent stem cells from mouse embryonic and adult fibroblast cultures by defined factors. Cell *126*, 663–676. https://doi.org/10.1016/j.cell.2006.07.024.
25. Liang, W., Gasparyan, L., AlQarawi, W., and Davis, D.R. (2019). Disease modeling of cardiac arrhythmias using human induced pluripotent stem cells. Expert Opin. Biol. Ther. *19*, 313–333. https://doi.org/10.1080/14712598.2019.1575359.
26. Winbo, A., Ramanan, S., Eugster, E., Jovinge, S., Skinner, J.R., and Montgomery, J.M. (2020). Functional coculture of sympathetic neurons and cardiomyocytes derived from human-induced pluripotent stem cells. Am. J. Physiol. Heart Circ. Physiol. *319*, H927–H937. https://doi.org/10.1152/ajpheart.00546.2020.
27. Takayama, Y., Kushige, H., Akagi, Y., Suzuki, Y., Kumagai, Y., and Kida, Y.S. (2020). Selective Induction of Human Autonomic Neurons Enables Precise Control of Cardiomyocyte Beating. Sci. Rep. *10*, 9464. https://doi.org/10.1038/s41598-020-66303-3.
28. Li, N., Edel, M., Liu, K., Denning, C., Betts, J., Neely, O.C., Li, D., and Paterson, D.J. (2023). Human induced pluripotent stem cell-derived cardiac myocytes and sympathetic neurons in disease modelling. Philos. Trans. R. Soc. Lond. B Biol. Sci. *378*, 20220173. https://doi.org/10.1098/rstb.2022.0173.
29. Oh, Y., Cho, G.S., Li, Z., Hong, I., Zhu, R., Kim, M.J., Kim, Y.J., Tampakakis, E., Tung, L., Huganir, R., et al. (2016). Functional Coupling with Cardiac Muscle Promotes Maturation of hPSC-Derived Sympathetic Neurons. Cell Stem Cell *19*, 95–106. https://doi.org/10.1016/j.stem.2016.05.002.
30. Frith, T.J.R., and Tsakiridis, A. (2019). Efficient Generation of Trunk Neural Crest and Sympathetic Neurons from Human Pluripotent Stem Cells Via a Neuromesodermal Axial Progenitor Intermediate. Curr. Protoc. Stem Cel Biol. *49*, e81. https://doi.org/10.1002/cpsc.81.
31. Kirino, K., Nakahata, T., Taguchi, T., and Saito, M.K. (2018). Efficient derivation of sympathetic neurons from human pluripotent stem cells with a defined condition. Sci. Rep. *8*, 12865. https://doi.org/10.1038/s41598-018-31256-1.
32. Saito-Diaz, K., Wu, H.F., and Zeltner, N. (2019). Autonomic Neurons with Sympathetic Character Derived From Human Pluripotent Stem Cells. Curr. Protoc. Stem Cel Biol. *49*, e78. https://doi.org/10.1002/cpsc.78.
33. Wu, H.F., and Zeltner, N. (2019). Overview of Methods to Differentiate Sympathetic Neurons from Human Pluripotent Stem Cells. Curr. Protoc. Stem Cel Biol. *50*, e92. https://doi.org/10.1002/cpsc.92.

34. Schmidt, K., Ralle, M., Schaffer, T., Jayakanthan, S., Bari, B., Muchenditsi, A., and Lutsenko, S. (2018). ATP7A and ATP7B copper transporters have distinct functions in the regulation of neuronal dopamine-β-hydroxylase. J. Biol. Chem. *293*, 20085–20098. https://doi.org/10.1074/jbc.RA118.004889.

35. Lopes, F.M., Schröder, R., da Frota, M.L.C., Jr., Zanotto-Filho, A., Müller, C.B., Pires, A.S., Meurer, R.T., Colpo, G.D., Gelain, D.P., Kapczinski, F., et al. (2010). Comparison between proliferative and neuron-like SH-SY5Y cells as an in vitro model for Parkinson disease studies. Brain Res. *1337*, 85–94. https://doi.org/10.1016/j.brainres.2010.03.102.

36. Kovalevich, J., Santerre, M., and Langford, D. (2021). Considerations for the Use of SH-SY5Y Neuroblastoma Cells in Neurobiology. Methods Mol. Biol. *2311*, 9–23. https://doi.org/10.1007/978-1-0716-1437-2_2.

37. Al-Maswary, A.A., O'Reilly, M., Holmes, A.P., Walmsley, A.D., Cooper, P.R., and Scheven, B.A. (2022). Exploring the neurogenic differentiation of human dental pulp stem cells. PLoS One *17*, e0277134. https://doi.org/10.1371/journal.pone.0277134.

38. Kume, T., Kawato, Y., Osakada, F., Izumi, Y., Katsuki, H., Nakagawa, T., Kaneko, S., Niidome, T., Takada-Takatori, Y., and Akaike, A. (2008). Dibutyryl cyclic AMP induces differentiation of human neuroblastoma SH-SY5Y cells into a noradrenergic phenotype. Neurosci. Lett. *443*, 199–203. https://doi.org/10.1016/j.neulet.2008.07.079.

39. England, J., and Loughna, S. (2013). Heavy and light roles: myosin in the morphogenesis of the heart. Cell. Mol. Life Sci. *70*, 1221–1239. https://doi.org/10.1007/s00018-012-1131-1.

40. De Bortoli, M., Meraviglia, V., Mackova, K., Frommelt, L.S., König, E., Rainer, J., Volani, C., Benzoni, P., Schlittler, M., Cattelan, G., et al. (2023). Modeling incomplete penetrance in arrhythmogenic cardiomyopathy by human induced pluripotent stem cell derived cardiomyocytes. Comput. Struct. Biotechnol. J. *21*, 1759–1773. https://doi.org/10.1016/j.csbj.2023.02.029.

41. Meraviglia, V., Cattelan, G., De Bortoli, M., Motta, B.M., Volpato, C., Frommelt, L. S., Rauhe, W., Di Segni, M., Silipigni, R., Pramstaller, P.P., and Rossini, A. (2021). Generation and characterization of three human induced pluripotent stem cell lines (EURACi007-A, EURACi008-A, EURACi009-A) from three different individuals of the same family with arrhythmogenic cardiomyopathy (ACM) carrying the plakophillin2 p.N346Lfs*12 mutation. Stem Cel Res. *55*, 102466. https://doi.org/10.1016/j.scr.2021.102466.

42. Kawashima, K., Mashimo, M., Nomura, A., and Fujii, T. (2024). Contributions of Non-Neuronal Cholinergic Systems to the Regulation of Immune Cell Function, Highlighting the Role of α7 Nicotinic Acetylcholine Receptors. Int. J. Mol. Sci. *25*, 4564. https://doi.org/10.3390/ijms25084564.

43. Narkar, A., Feaster, T.K., Casciola, M., and Blinova, K. (2022). Human in vitro neurocardiac coculture (ivNCC) assay development for evaluating cardiac contractility modulation. Physiol. Rep. *10*, e15498. https://doi.org/10.14814/phy2.15498.

44. Dunn, M., Henke, A., Clark, S., Kovalyova, Y., Kempadoo, K.A., Karpowicz, R.J., Jr., Kandel, E.R., Sulzer, D., and Sames, D. (2018). Designing a norepinephrine optical tracer for imaging individual noradrenergic synapses and their activity in vivo. Nat. Commun. *9*, 2838. https://doi.org/10.1038/s41467-018-05075-x.

45. Garg, P., Garg, V., Shrestha, R., Sanguinetti, M.C., Kamp, T.J., and Wu, J.C. (2018). Human Induced Pluripotent Stem Cell-Derived Cardiomyocytes as Models for Cardiac Channelopathies: A Primer for Non-Electrophysiologists. Circ. Res. *123*, 224–243. https://doi.org/10.1161/circresaha.118.311209.

46. Kussauer, S., David, R., and Lemcke, H. (2019). hiPSCs Derived Cardiac Cells for Drug and Toxicity Screening and Disease Modeling: What Micro- Electrode-Array Analyses Can Tell Us. Cells *8*, 1331. https://doi.org/10.3390/cells8111331.

47. Moretti, A., Laugwitz, K.L., Dorn, T., Sinnecker, D., and Mummery, C. (2013). Pluripotent stem cell models of human heart disease. Cold Spring Harb. Perspect. Med. *3*, a014027. https://doi.org/10.1101/cshperspect.a014027.

48. Kim, C., Wong, J., Wen, J., Wang, S., Wang, C., Spiering, S., Kan, N.G., Forcales, S., Puri, P.L., Leone, T.C., et al. (2013). Studying arrhythmogenic right ventricular dysplasia with patient-specific iPSCs. Nature *494*, 105–110. https://doi.org/10.1038/nature11799.

49. Caspi, O., Huber, I., Gepstein, A., Arbel, G., Maizels, L., Boulos, M., and Gepstein, L. (2013). Modeling of arrhythmogenic right ventricular cardiomyopathy with human induced pluripotent stem cells. Circ. Cardiovasc. Genet. *6*, 557–568. https://doi.org/10.1161/circgenetics.113.000188.

50. Ma, D., Wei, H., Lu, J., Ho, S., Zhang, G., Sun, X., Oh, Y., Tan, S.H., Ng, M.L., Shim, W., et al. (2013). Generation of patient-specific induced pluripotent stem cell-derived cardiomyocytes as a cellular model of arrhythmogenic right ventricular cardiomyopathy. Eur. Heart J. *34*, 1122–1133. https://doi.org/10.1093/eurheartj/ehs226.

51. Vanaja, I.P., Scalco, A., Ronfini, M., Bona, A.D., Olianti, C., Rizzo, S., Chelko, S.P., Corrado, D., Sacconi, L., Basso, C., et al. (2025). Cardiac sympathetic neurons are additional cells affected in genetically determined arrhythmogenic cardiomyopathy. J. Physiol. *603*, 1959–1982. https://doi.org/10.1113/jp286845.

52. Moretti, A., Bellin, M., Welling, A., Jung, C.B., Lam, J.T., Bott-Flügel, L., Dorn, T., Goedel, A., Höhnke, C., Hofmann, F., et al. (2010). Patient-specific induced pluripotent stem-cell models for long-QT syndrome. N. Engl. J. Med. *363*, 1397–1409. https://doi.org/10.1056/NEJMoa0908679.

53. Liang, P., Lan, F., Lee, A.S., Gong, T., Sanchez-Freire, V., Wang, Y., Diecke, S., Sallam, K., Knowles, J.W., Wang, P.J., et al. (2013). Drug screening using a library of human induced pluripotent stem cell-derived cardiomyocytes reveals disease-specific patterns of cardiotoxicity. Circulation *127*, 1677–1691. https://doi.org/10.1161/circulationaha.113.001883.

54. Winbo, A., Ramanan, S., Eugster, E., Rydberg, A., Jovinge, S., Skinner, J.R., and Montgomery, J.M. (2021). Functional hyperactivity in long QT syndrome type 1 pluripotent stem cell-derived sympathetic neurons. Am. J. Physiol. Heart Circ. Physiol. *321*, H217–H227. https://doi.org/10.1152/ajpheart.01002.2020.

55. Egashira, T., Yuasa, S., Suzuki, T., Aizawa, Y., Yamakawa, H., Matsuhashi, T., Ohno, Y., Tohyama, S., Okata, S., Seki, T., et al. (2012). Disease characterization using LQTS-specific induced pluripotent stem cells. Cardiovasc. Res. *95*, 419–429. https://doi.org/10.1093/cvr/cvs206.

56. Chua, C.J., Morrissette-McAlmon, J., Tung, L., and Boheler, K.R. (2023). Understanding Arrhythmogenic Cardiomyopathy: Advances through the Use of Human Pluripotent Stem Cell Models. Genes (Basel) *14*, 1864. https://doi.org/10.3390/genes14101864.

57. Reisqs, J.B., Moreau, A., Sleiman, Y., Boutjdir, M., Richard, S., and Chevalier, P. (2023). Arrhythmogenic cardiomyopathy as a myogenic disease: highlights from cardiomyocytes derived from human induced pluripotent stem cells. Front. Physiol. *14*, 1191965. https://doi.org/10.3389/fphys.2023.1191965.

58. Benzoni, P., Campostrini, G., Landi, S., Bertini, V., Marchina, E., Iascone, M., Ahlberg, G., Olesen, M.S., Crescini, E., Mora, C., et al. (2020). Human iPSC modelling of a familial form of atrial fibrillation reveals a gain of function of If and ICaL in patient-derived cardiomyocytes. Cardiovasc. Res. *116*, 1147–1160. https://doi.org/10.1093/cvr/cvz217.

59. Grandi, E., and Dobrev, D. (2020). Atrial fibrillation in a dish: insights into atrial arrhythmogenesis from induced pluripotent stem cell-derived cardiomyocytes. Cardiovasc. Res. *116*, 1089–1091. https://doi.org/10.1093/cvr/cvz342.

60. Bose, A., Petsko, G.A., and Studer, L. (2022). Induced pluripotent stem cells: a tool for modeling Parkinson's disease. Trends Neurosci. *45*, 608–620. https://doi.org/10.1016/j.tins.2022.05.001.

61. Zeltner, N., Fattahi, F., Dubois, N.C., Saurat, N., Lafaille, F., Shang, L., Zimmer, B., Tchieu, J., Soliman, M.A., Lee, G., et al. (2016). Capturing the biology of disease severity in a PSC-based model of familial dysautonomia. Nat. Med. *22*, 1421–1427. https://doi.org/10.1038/nm.4220.

62. Wu, H.F., Yu, W., Saito-Diaz, K., Huang, C.W., Carey, J., Lefcort, F., Hart, G.W., Liu, H.X., and Zeltner, N. (2022). Norepinephrine transporter defects lead to sympathetic hyperactivity in Familial Dysautonomia models. Nat. Commun. *13*, 7032. https://doi.org/10.1038/s41467-022-34811-7.

63. Wu, H.F., and Zeltner, N. (2020). Efficient Differentiation of Postganglionic Sympathetic Neurons using Human Pluripotent Stem Cells under Feeder-free and Chemically Defined Culture Conditions. J. Vis. Exp. *159*, e60843. https://doi.org/10.3791/60843.

64. Bernardin, A.A., Colombani, S., Rousselot, A., Andry, V., Goumon, Y., Delanoë-Ayari, H., Pasqualin, C., Brugg, B., Jacotot, E.D., Pasquié, J.L., et al. (2022). Impact of Neurons on Patient-Derived Cardiomyocytes Using Organ-On-A-Chip and iPSC Biotechnologies. Cells *11*, 3764. https://doi.org/10.3390/cells11233764.

65. Biffi, E., Regalia, G., Menegon, A., Ferrigno, G., and Pedrocchi, A. (2013). The influence of neuronal density and maturation on network activity of hippocampal cell cultures: a methodological study. PLoS One *8*, e83899. https://doi.org/10.1371/journal.pone.0083899.

66. Geden, M.J., Romero, S.E., and Deshmukh, M. (2019). Apoptosis versus axon pruning: Molecular intersection of two distinct pathways for axon degeneration. Neurosci. Res. *139*, 3–8. https://doi.org/10.1016/j.neures.2018.11.007.

67. Franzoso, M., Zaglia, T., and Mongillo, M. (2016). Putting together the clues of the everlasting neuro-cardiac liaison. Biochim. Biophys. Acta *1863*, 1904–1915. https://doi.org/10.1016/j.bbamcr.2016.01.009.

68. Takeuchi, A., Nakafutami, S., Tani, H., Mori, M., Takayama, Y., Moriguchi, H., Kotani, K., Miwa, K., Lee, J.K., Noshiro, M., and Jimbo, Y. (2011). Device for co-culture of sympathetic neurons and cardiomyocytes using microfabrication. Lab. Chip *11*, 2268–2275. https://doi.org/10.1039/c0lc00327a.

69. Larsen, H.E., Lefkimmiatis, K., and Paterson, D.J. (2016). Sympathetic neurons are a powerful driver of myocyte function in cardiovascular disease. Sci. Rep. *6*, 38898. https://doi.org/10.1038/srep38898.

70. Davis, H., Liu, K., Li, N., Li, D., and Paterson, D.J. (2022). Healthy cardiac myocytes can decrease sympathetic hyperexcitability in the early stages of hypertension. Front. Synaptic Neurosci. *14*, 949150. https://doi.org/10.3389/fnsyn.2022.949150.

71. Miwa, K., Lee, J.K., Takagishi, Y., Opthof, T., Fu, X., Hirabayashi, M., Watabe, K., Jimbo, Y., Kodama, I., and Komuro, I. (2013). Axon guidance of sympathetic neurons to cardiomyocytes by glial cell line-derived neurotrophic factor (GDNF). PLoS One *8*, e65202. https://doi.org/10.1371/journal.pone.0065202.

72. Häkli, M., Jäntti, S., Joki, T., Sukki, L., Tornberg, K., Aalto-Setälä, K., Kallio, P., Pekkanen-Mattila, M., and Narkilahti, S. (2022). Human Neurons Form Axon-Mediated Functional Connections with Human Cardiomyocytes in Compartmentalized Microfluidic Chip. Int. J. Mol. Sci. *23*, 3148. https://doi.org/10.3390/ijms23063148.

73. Reeser, R.S., Salazar, A.K., Prutton, K.M., Roede, J.R., VeDepo, M.C., and Jacot, J.G. (2024). Trisomy 21 Alters Cell Proliferation and Migration of iPSC-Derived Cardiomyocytes on Type VI Collagen. Cell. Mol. Bioeng. *17*, 25–34. https://doi.org/10.1007/s12195-023-00791-x.

74. Moyes, K.W., Sip, C.G., Obenza, W., Yang, E., Horst, C., Welikson, R.E., Hauschka, S.D., Folch, A., and Laflamme, M.A. (2013). Human embryonic stem cell-derived cardiomyocytes migrate in response to gradients of fibronectin and Wnt5a. Stem Cell Dev. *22*, 2315–2325. https://doi.org/10.1089/scd.2012.0586.

75. Kc, P., Shah, M., Shaik, R., Hong, Y., and Zhang, G. (2020). Preseeding of Mesenchymal Stem Cells Increases Integration of an iPSC-Derived CM Sheet into a Cardiac Matrix. ACS Biomater. Sci. Eng. *6*, 6808–6818. https://doi.org/10.1021/acsbiomaterials.0c00788.

76. Li, J., and Zheng, L. (2022). The Mechanism of Cardiac Sympathetic Activity Assessment Methods: Current Knowledge. Front. Cardiovasc. Med. *9*, 931219. https://doi.org/10.3389/fcvm.2022.931219.

77. Ferreira, M.J., Colombani, S., Bernardin, A., Lacampagne, A., Pasquié, J.-L., Costa, P. F., Charlot, B., and Meli, A.C. (2025). Advancing organ-on-chip systems: the role of microfluidics in neuro-cardiac research. Curr. Res. Pharmacol. Drug Discov. *9*, 100227. https://doi.org/10.1016/j.crphar.2025.100227.

78. Weinberger, F., Mannhardt, I., and Eschenhagen, T. (2017). Engineering Cardiac Muscle Tissue: A Maturating Field of Research. Circ. Res. *120*, 1487–1500. https://doi.org/10.1161/CIRCRESAHA.117.310738.

79. Mummery, C.L. (2018). Perspectives on the Use of Human Induced Pluripotent Stem Cell-Derived Cardiomyocytes in Biomedical Research. Stem Cel Rep. *11*, 1306–1311. https://doi.org/10.1016/j.stemcr.2018.11.011.

80. Eder, A., Vollert, I., Hansen, A., and Eschenhagen, T. (2016). Human engineered heart tissue as a model system for drug testing. Adv. Drug Deliv. Rev. *96*, 214–224. https://doi.org/10.1016/j.addr.2015.05.010.

81. Denning, C., Borgdorff, V., Crutchley, J., Firth, K.S.A., George, V., Kalra, S., Kondrashov, A., Hoang, M.D., Mosqueira, D., Patel, A., et al. (2016). Cardiomyocytes from human pluripotent stem cells: From laboratory curiosity to industrial biomedical platform. Biochim. Biophys. Acta *1863*, 1728–1748. https://doi.org/10.1016/j.bbamcr.2015.10.014.

82. Frith, T.J., Granata, I., Wind, M., Stout, E., Thompson, O., Neumann, K., Stavish, D., Heath, P.R., Ortmann, D., Hackland, J.O., et al. (2018). Human axial progenitors generate trunk neural crest cells in vitro. Elife *7*, e35786. https://doi.org/10.7554/eLife.35786.

83. Buoso, C., Seifert, M., Lang, M., Griffith, C.M., Talavera Andújar, B., Castelo Rueda, M.P., Fischer, C., Doerrier, C., Talasz, H., Zanon, A., et al. (2024). Dopamine-iron homeostasis interaction rescues mitochondrial fitness in Parkinson's disease. Neurobiol. Dis. *196*, 106506. https://doi.org/10.1016/j.nbd.2024.106506.

YMTHE, Volume *34*

Supplemental Information

A reproducible and physiologically relevant human iPSC-derived platform for *in vitro* modeling of the neurocardiac junction

Giada Cattelan, Claudia Altomare, Giovanna Gentile, Alexandros A. Lavdas, Laura Sophie Frommelt, Chiara Volani, Luisa Foco, Pietro Girardi, Elisa Peducci, Amparo Guerrero Gerboles, Michele Miragoli, Luisa Petti, Peter P. Pramstaller, Lucio Barile, Serena Zacchigna, Irene Pichler, Alessandra Rossini, Alessandra Zanon, and Marzia De Bortoli

SUPPLEMENTAL FILES

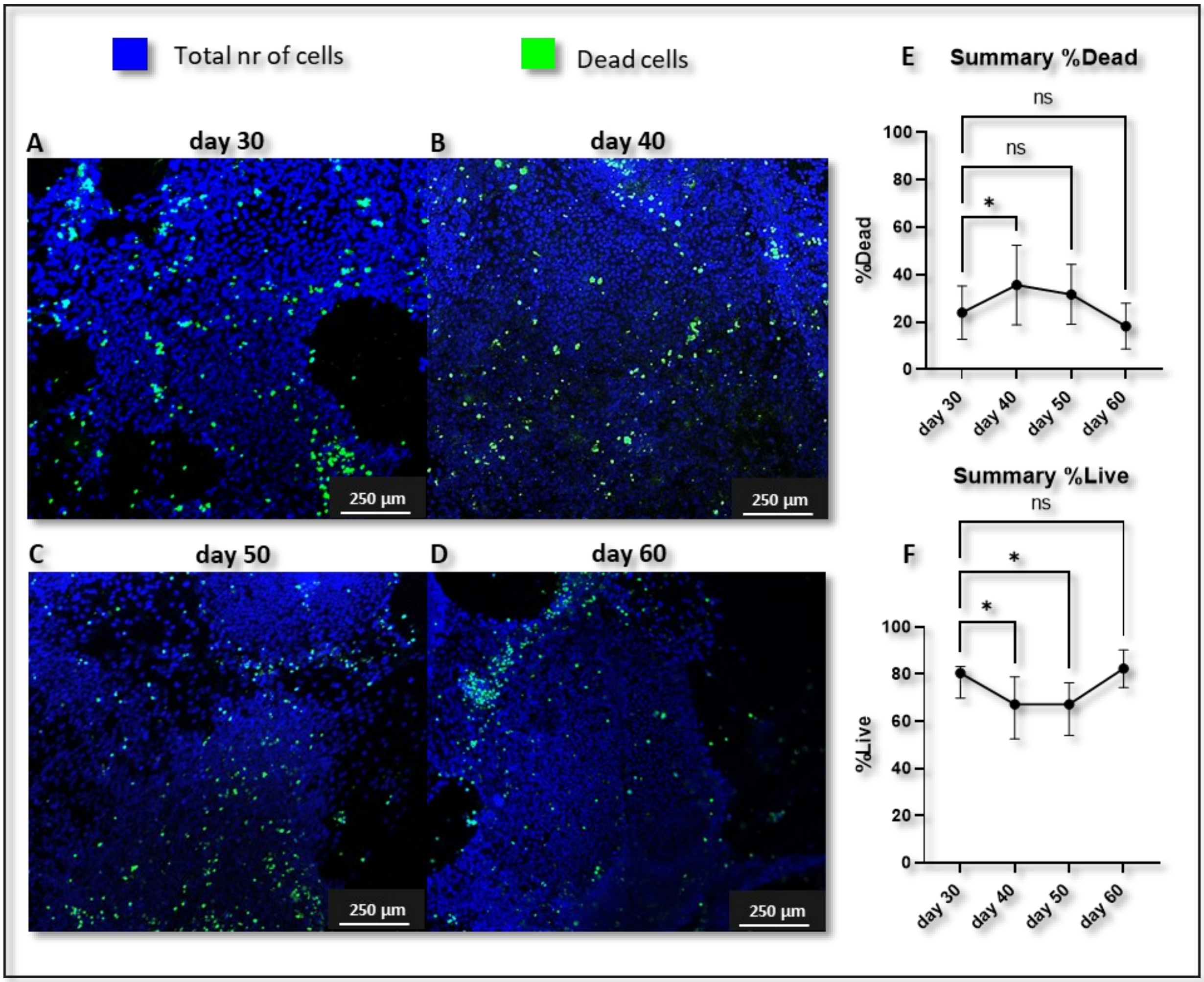

Figure S1: Live and Dead immunostaining of hiPSC-SNs at different timepoints does not show an excessive cell death during the prolonged time in culture.

A-D: Representative images of hiPSC-SN at day 30, day 40, day 50 and day 60 stained in live with the Live and Dead assay. Blue= NucBlue (total number of cells). Green= NucGreen (dead cells). Live cells were obtained subtracting to the total nr. of cells the amount of dead cells. Scale bar 250 µm.
E: Quantitative analysis of the percentage of Dead Cells. IQR with median of 3 differentiations. Test used Kruskal-Wallis: day 30 vs. day 40 p= 0.028
F: Quantitative analysis of the percentage of Live Cells. IQR with median of 3 differentiations. Test used Kruskal-Wallis: day 30 vs. day 40 p= 0.034; day 30 vs. day 50 p= 0.048

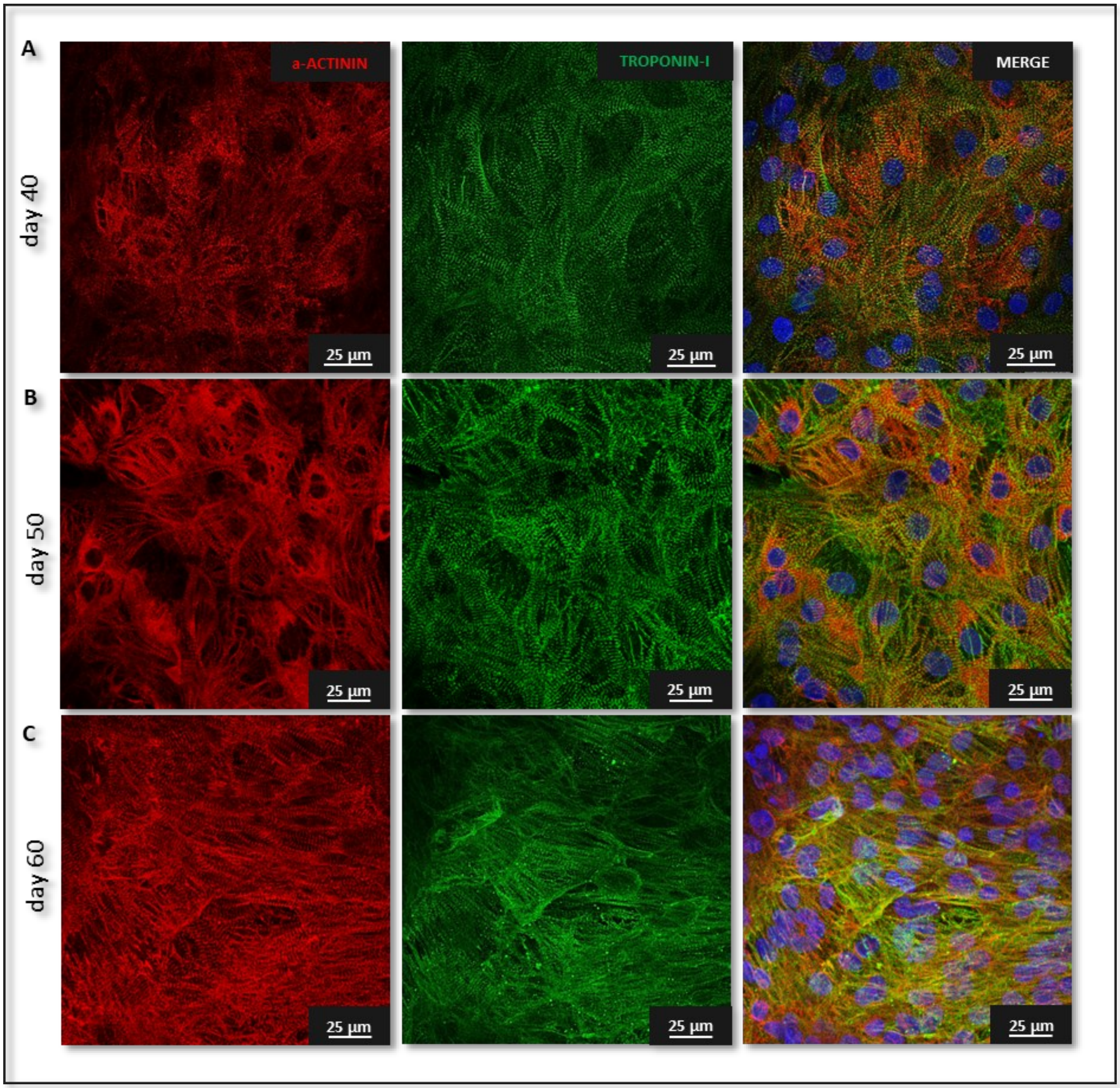

Figure S2: Representative images of hiPSC-CMs at different timepoints stained with sarcomeric markers.

A: hiPSC-CMs at day 40, B: hiPSC-CMs at day 50, C: hiPSC-CMs at day 60. α-actinin is stained in red, troponin-I in green and nuclei with DAPI. Scale bar 25 µm.

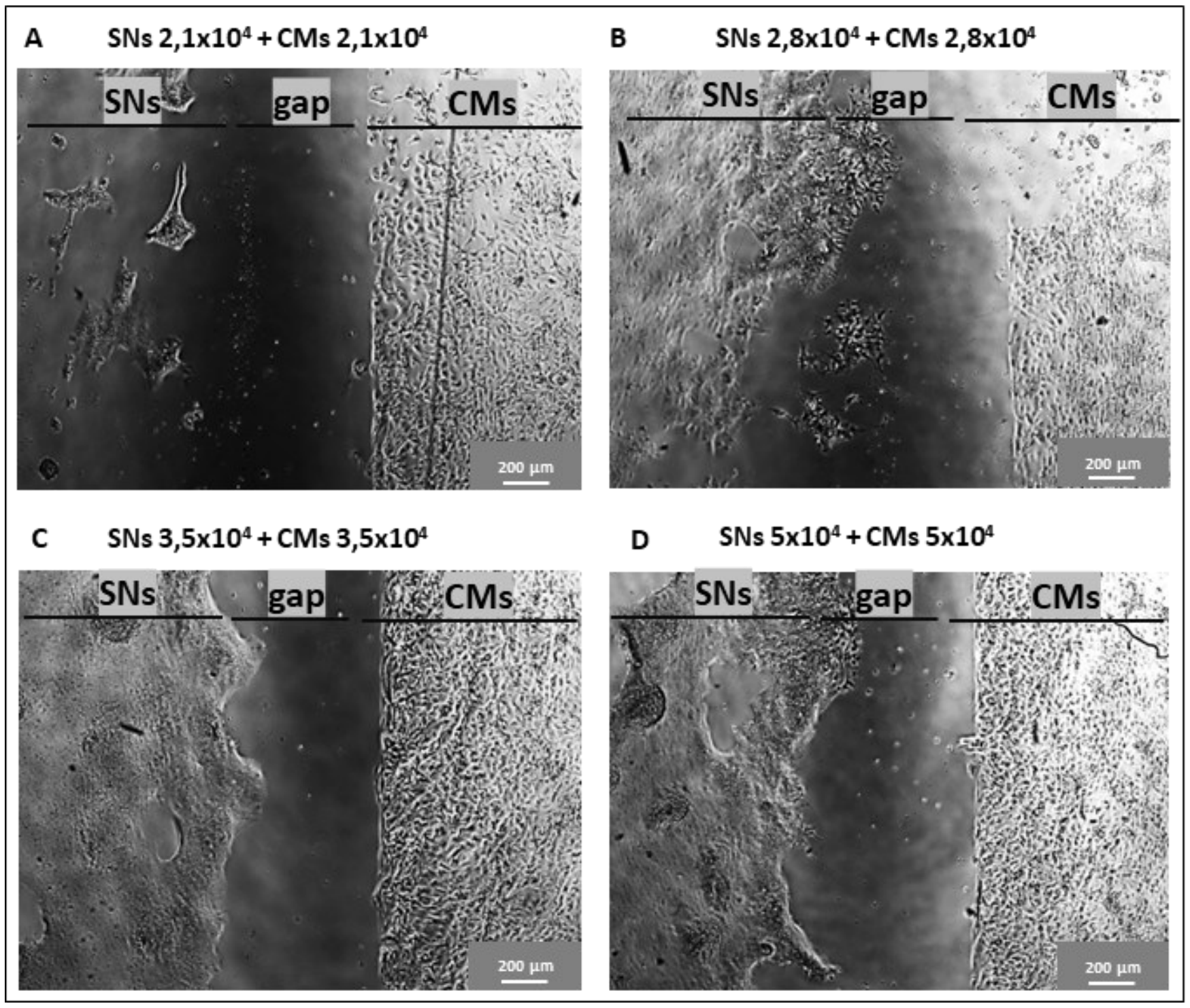

Figure S3: Different cell densities plated in the insert.

A: hiPSC-derived SNs and hiPSC-derived CMs plated at $2,1x10^4$ cell/chamber. Scale bar 200 µm.
B: hiPSC-derived SNs and iPSC-derived CMs plated at $2,8x10^4$ cell/chamber. Scale bar 200 µm.
C: hiPSC-derived SNs and iPSC-derived CMs plated at $3.5x10^4$ cell/chamber. Scale bar 200 µm.
D: hiPSC-derived SNs and iPSC-derived CMs plated at $5x10^4$ cell/chamber. Scale bar 200 µm.

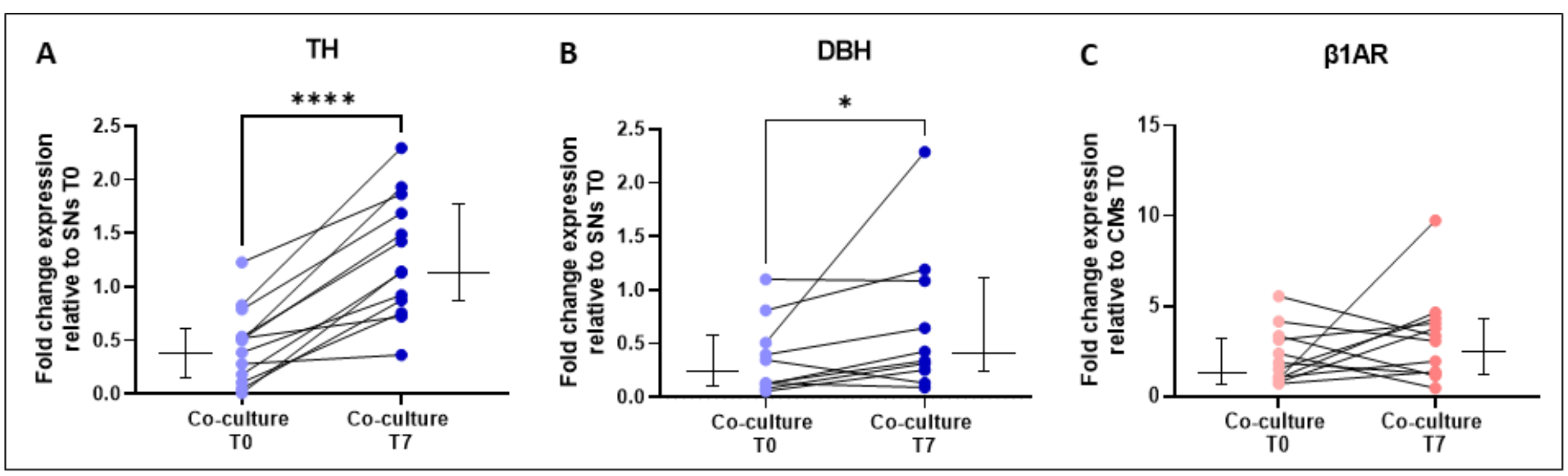

Figure S4: *TH, DBH and β1AR* gene expression analysis after 7 days of co-cultures.

A-C: qRT-PCR of co-cultures at timepoint T0 and timepoint T7.
A: *TH* expression of co-cultures at timepoint T0 vs. timepoint T7. Median with IQR of 13 differentiations with 1 replicate. Wilcoxon test; p value: co-cultures at timepoint T0 vs. timepoint T7 = 0.0002
B: *DBH* expression of co-cultures at timepoint T0 vs. timepoint T7. Median with IQR of 10 differentiations with 1 replicate. Wilcoxon test; p value: co-cultures at timepoint T0 vs. timepoint T7 =0.048
C: *β1AR* expression of co-cultures at timepoint T0 vs. timepoint T7. Median with IQR of 12 differentiations with 1 replicate. Wilcoxon test; p value: co-cultures at timepoint T0 vs. timepoint T7 = Not significant.

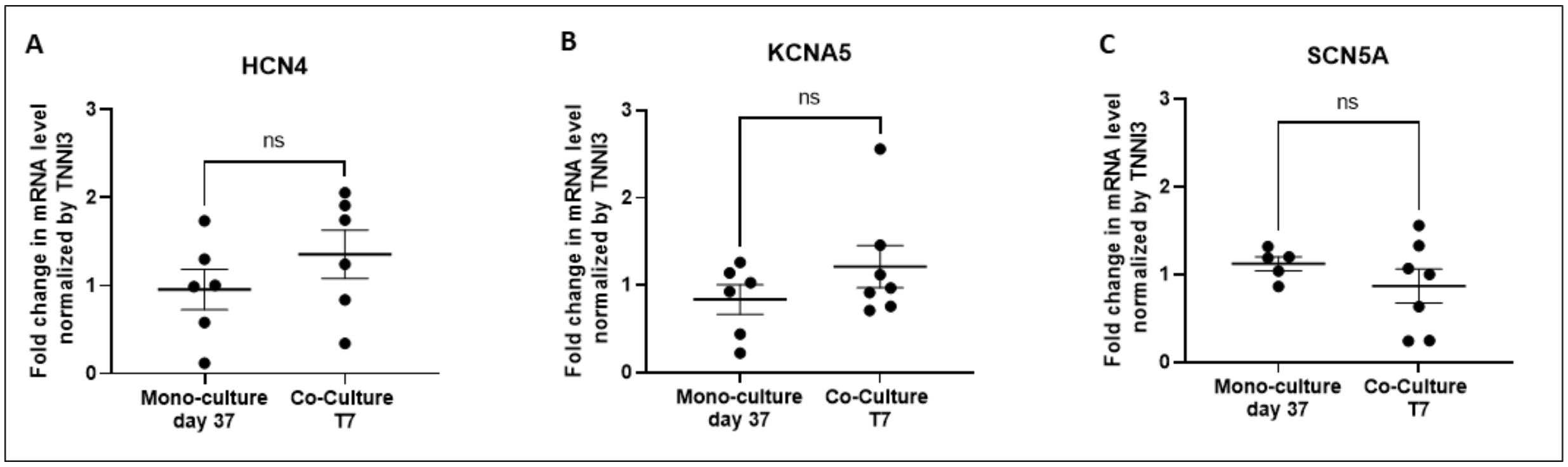

Figure S5: Ion channel genes expression analysis in mono and co-culture hiPSC-CMs.

A-C: qRT-PCR of mono-cultures day 37 and co-cultures T7, corresponding to the same differentiation age.
A: *HCN4* expression of mono-cultures day 37 vs. co-cultures T7. Mean with SEM of 5-7 differentiations with 1 replicate. Unpaired t-test; p value: mono-cultures day 37 and co-cultures T7 = Not significant.
B: *KCNA5* expression of mono-cultures day 37 vs. co-cultures T7. Median with IQR of 5-7 differentiations with 1 replicate. Mann-Whitney test; p value: mono-cultures day 37 and co-cultures T7 = Not significant.
C: *SCN5A* expression of mono-cultures day 37 vs. co-cultures T7. Mean with SEM of 5-7 differentiations with 1 replicate. Unpaired t-test; p value: mono-cultures day 37 and co-cultures T7 = Not significant.

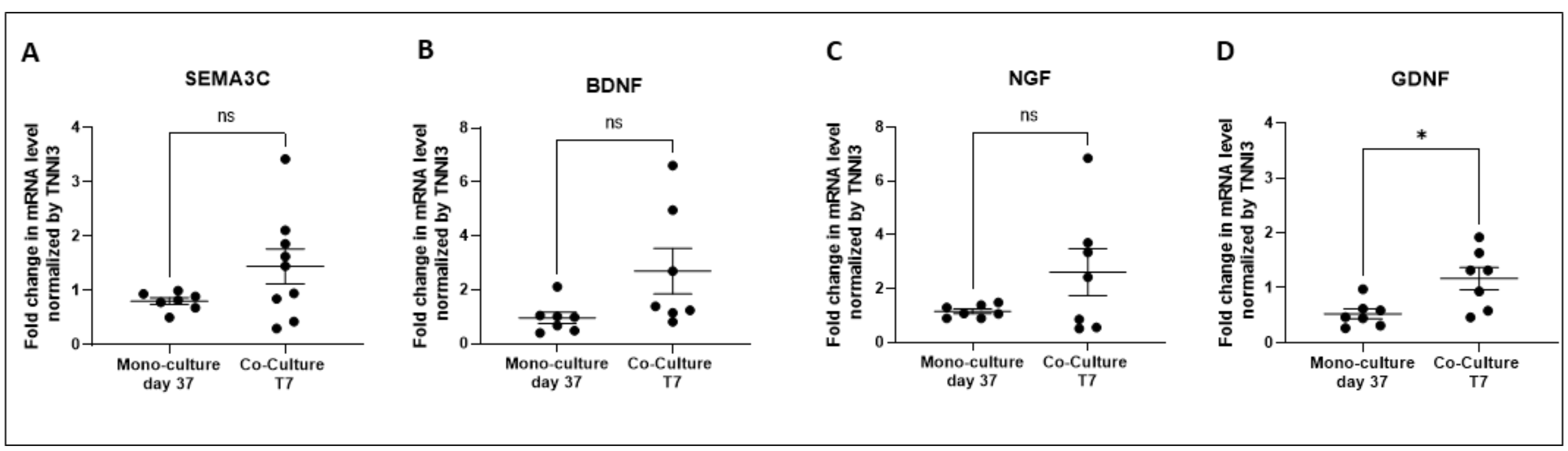

Figure S6: Axon guidance genes expression analysis in mono and co-culture hiPSC-CMs.

A-C: qRT-PCR of mono-culture day 37 and co-cultures T7, corresponding to the same differentiation age
A: *SEMA3C* expression of mono-cultures day 37 vs. co-cultures T7. Mean with SEM of 7-9 differentiations with 1 replicate. Unpaired t-test; p value: mono-cultures day 37 and co-cultures T7 = Not significant.
B: *BDNF* expression of mono-cultures day 37 vs. co-cultures T7. Mean with SEM of 7-9 differentiations with 1 replicate. Unpaired t-test; p value: mono-cultures day 37 and co-cultures T7=Not significant.
C: *NGF* expression of mono-cultures day 37 vs. co-cultures T7. Mean with SEM of 7-9 differentiations with 1 replicate. Unpaired t-test; p value: mono-cultures day 37 and co-cultures T7 = Not significant.
D: *GDNF* expression of mono-cultures day 37 vs. co-cultures T7. Mean with SEM of 7-9 differentiations with 1 replicate. Unpaired t-test; p value: mono-cultures day 37 and co-cultures T7 = 0.013.

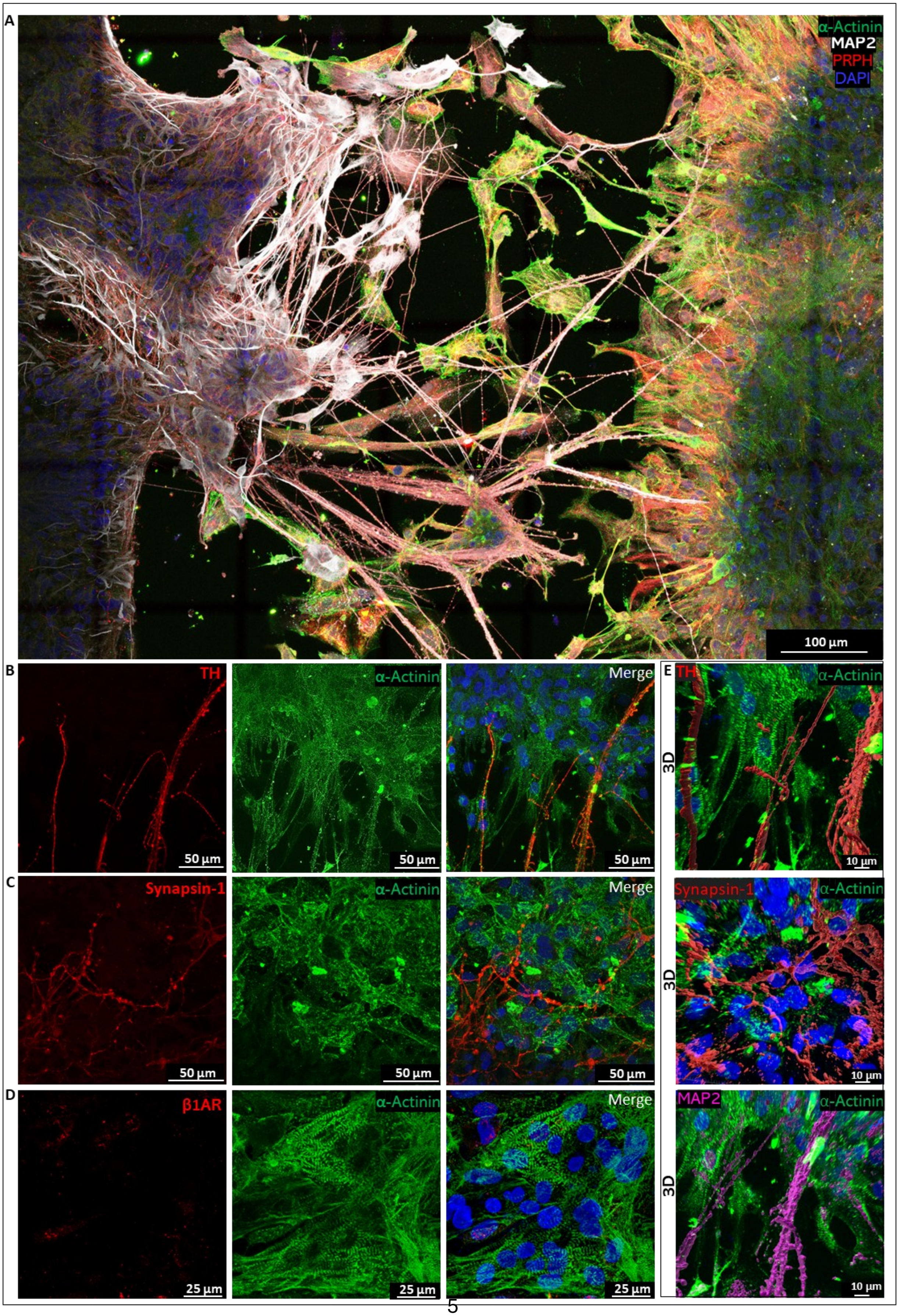
A
α-Actinin
MAP2
PRPH
DAPI
100 µm
B
TH
50 µm
α-Actinin
50 µm
Merge
50 µm
C
Synapsin-1
50 µm
α-Actinin
50 µm
Merge
50 µm
D
β1AR
25 µm
α-Actinin
25 µm
Merge
25 µm
E
TH
α-Actinin
3D
10 µm
Synapsin-1
α-Actinin
3D
10 µm
MAP2
α-Actinin
3D
10 µm

Figure S7: Immunocytochemical images of CTR co-cultures.

A: Reconstruction of a large area of a neurocardiac co-culture. Immunostaining experiment showing neuronal axons in red (PRPH) and white (MAP2), and cardiomyocytes in green (sarcomeric α-actinin). Nuclei were stained with DAPI (blue). Scale bar 100 μm.

B-D: Qualitative immunostaining images of co-culture models at timepoint T7.

B: CTR hiPSC-SNs stained with TH (red) and CTR hiPSC-CMs stained with α-actinin (green). Scale bar 50 μm.

C: CTR hiPSC-SNs stained with Synapsin-1 (red) and CTR hiPSC-CMs stained with α-actinin (green). Scale bar 50 μm.

D: CTR hiPSC-CMs stained with β1AR (red) and α-actinin (green). Scale bar 50 μm.

E: 3D reconstruction of the co-cultures. Scale bar 10 μm. Nuclei were counterstained with DAPI (blu).

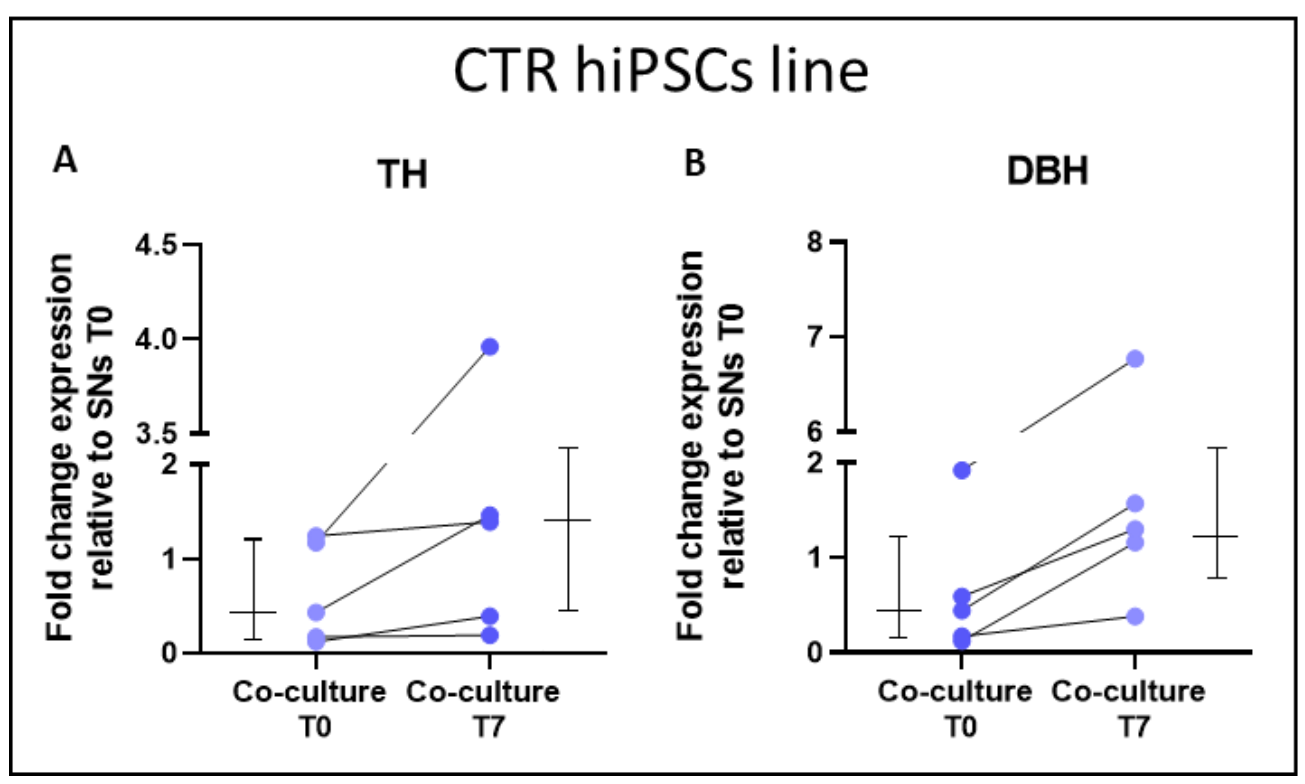

Figure S8: ***TH and DBH*** **gene expression analysis in CTR hiPSCs Line after 7 days of co-cultures.**

A-B: qRT-PCR of CTR co-cultures at timepoint T0 and timepoint T7.

A: *TH* expression of co-cultures at timepoint T0 vs. timepoint T7. Median with IQR of 5 differentiations with 1 replicate. Paired-t test; p value: co-cultures at timepoint T0 vs. timepoint T7 = Not significant.

B: *DBH* expression of co-cultures at timepoint T0 vs. timepoint T7. Median with IQR of 5 differentiations with 1 replicate. Wilcoxon test; p value: co-cultures at timepoint T0 vs. timepoint T7 = Not significant.

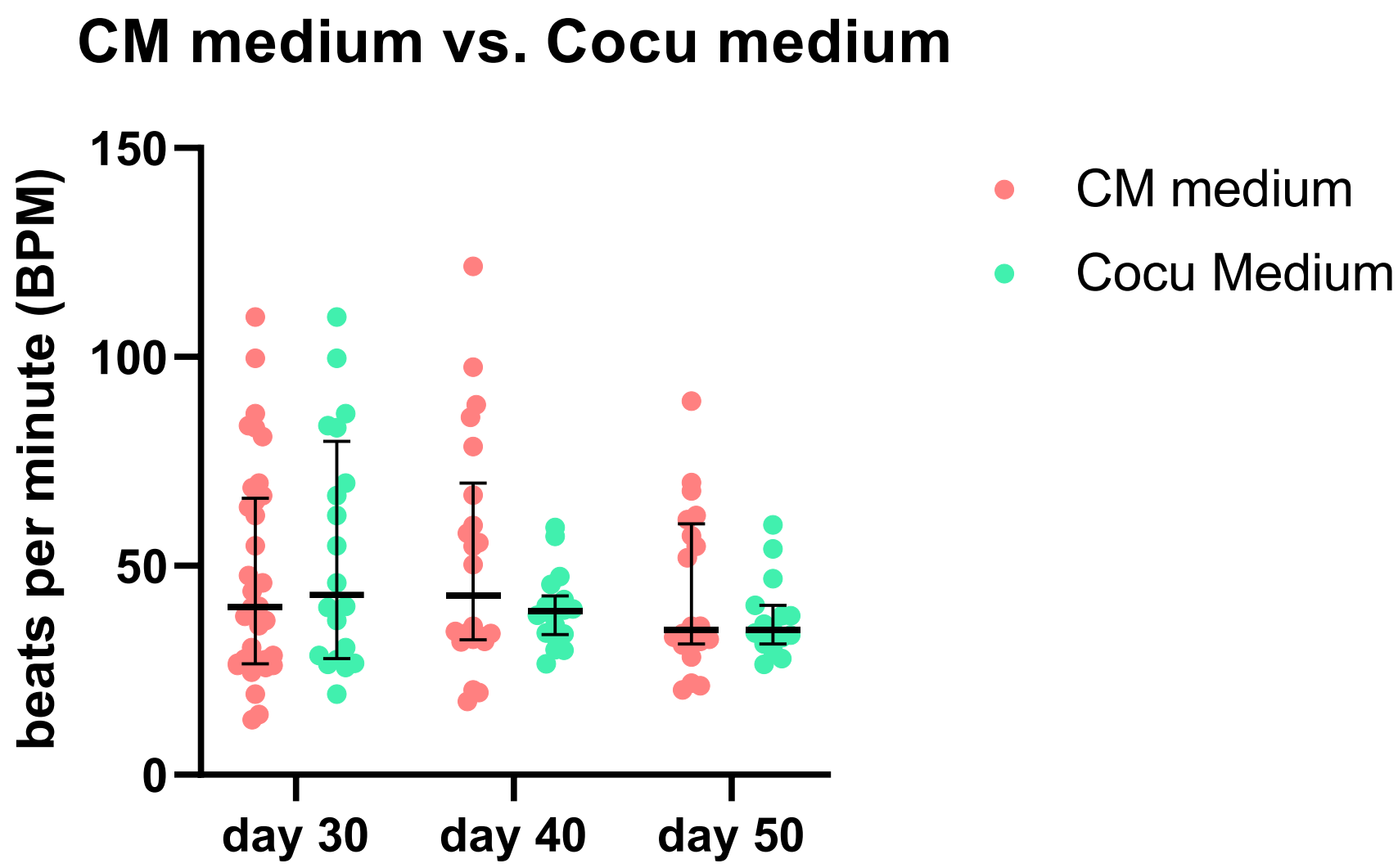

Figure S9: Comparison of the beating rate of monocultured hiPSC-CMs cultivated in standard basal medium or with conditioned co-culture medium.

Comparison of the beating rate of hiPSC-CMs cultured with two different media. The same batch of cells was cultivated with basal medium (RPMI + B27) or co-culture medium (1:1 basal medium of hiPSC-CMs and basal medium hiPSC-SNs). We observed no evidence of a significant difference in the number of beats when moving from the basal to the co-culture medium group (beta -4.96, 95% confidence interval -10.92; 1.00, p=0.103) and no evidence of a difference over time (beta -2.26, 95% CI -6,94; 2.42, p=0.345). Data were analyzed using a multilevel linear random intercept and random slope model (linear mixed effects model), to simultaneously account for both the hierarchical structure of the data (independent experiments) and their longitudinal nature (number of beats observed at different time points in the same cells for each experiment).

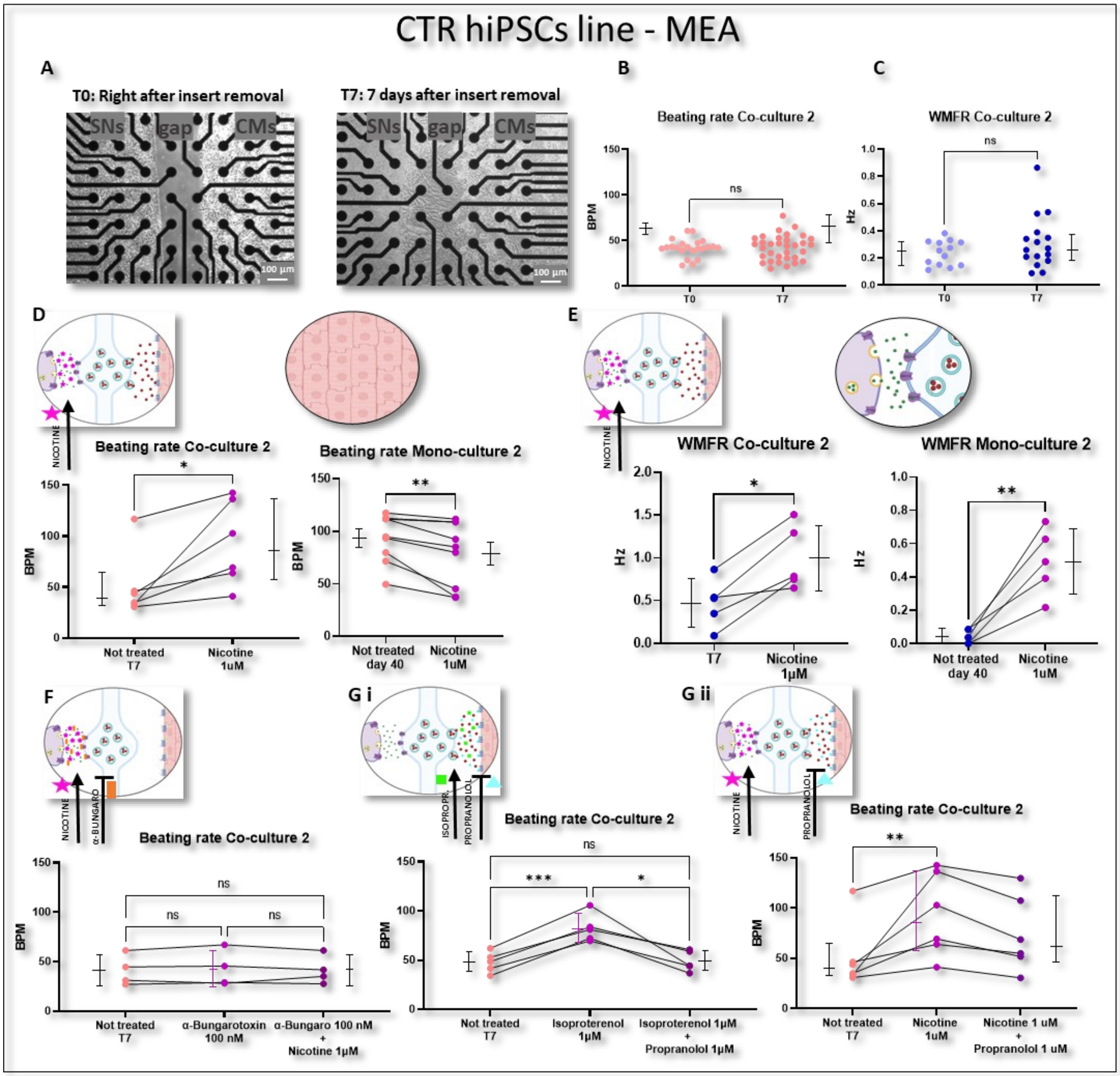

Figure S10: Functional analysis of co-cultures derived from a second CTR hiPSC line confirms that CTR hiPSC-SNs can modulate the beating activity of CTR hiPSC-CMs.

A: Brightfield images of co-cultures plated on MEA plates at the timepoint T0 and timepoint T7. Scale bar 100 µm. **B:** Spontaneous beating rate of CTR hiPSC-CMs in co-cultures evaluated at timepoint T0 (no connections between the two populations) and timepoint T7. Median with IQR of 7 differentiations with 3-6 replicates- each dot represents a technical replicate. Test used: Mann Whitney test: not significant.

C: Spontaneous WMFR of CTR hiPSC-SNs in co-cultures evaluated at timepoint T0 (no connections between the two populations) and timepoint T7. Median with IQR of 11 differentiations with 1-3 replicates - each dot represents a technical replicate; Test used: Mann Whitney test: not significant.

D: Beating rate of CTR hiPSC-CMs in co-cultures at timepoint T7 treated with 1 µM Nic vs. not treated co-culture. Median with IQR of 6 differentiations with 1 replicate. Test used: Wilcoxon test; p value: * = 0.031.

Beating rate of CTR hiPSC-CMs in mono-cultures at day 40 treated with 1 µM Nic vs. not treated mono-culture. Mean with SD of 7 differentiations with 2 replicates - each dot represents one technical replicate. Test used: Paired-t test; p value: **

= 0.007.

E: WMFR of CTR hiPSC-SNs in co-culture/mono-culture treated with 1 µM Nic vs. not treated co-culture/mono-culture. Co-culture vs Not treated (Mean with SD of 5 co-cultures with 1-5 replicates- each dot represents a technical replicate). Test used: Paired t-test; p value: * = 0.01. Mono-culture vs Not treated (Mean with SD of 5 co-cultures with 2-6 replicates- each dot represents a technical replicate). Test used: Paired t-test; p value: * = 0.006.

F: Beating rate of CTR hiPSC-CMs in not treated co-cultures and treated with 100 nM α-Bungarotoxin or α-Bungarotoxin + Nic. Co-culture vs Not treated (Mean with SD of 4 co-cultures with 1 replicate). Test used: Tukey multiple comparison test: not significant.

G i: Beating rate of CTR hiPSC-CMs in co-cultures treated with Isoproterenol (1 µM) vs. Isoproterenol (1 µM) + Propranolol (1 µM) vs. not treated (Mean with SD of 5 co-cultures with 1 replicate). Test used: Tukey multiple comparisons test; p value= Not treated co-cultures vs. Isoproterenol, p value: *** = 0.0007; Isoproterenol vs. Isoproterenol + Propranolol, p value: * = 0.022.

G ii: Beating rate of CTR hiPSC-CMs in co-cultures treated with 1 µM Nic vs. 1 µM Nic + 1 µM Propranolol vs. not treated (Median with IQR of 6 co-cultures with 1 replicate- each dot represents a technical replicate), Test used: Dunn's multiple comparisons test; Not treated co-cultures vs. Nicotine, p value: ** = 0.004.

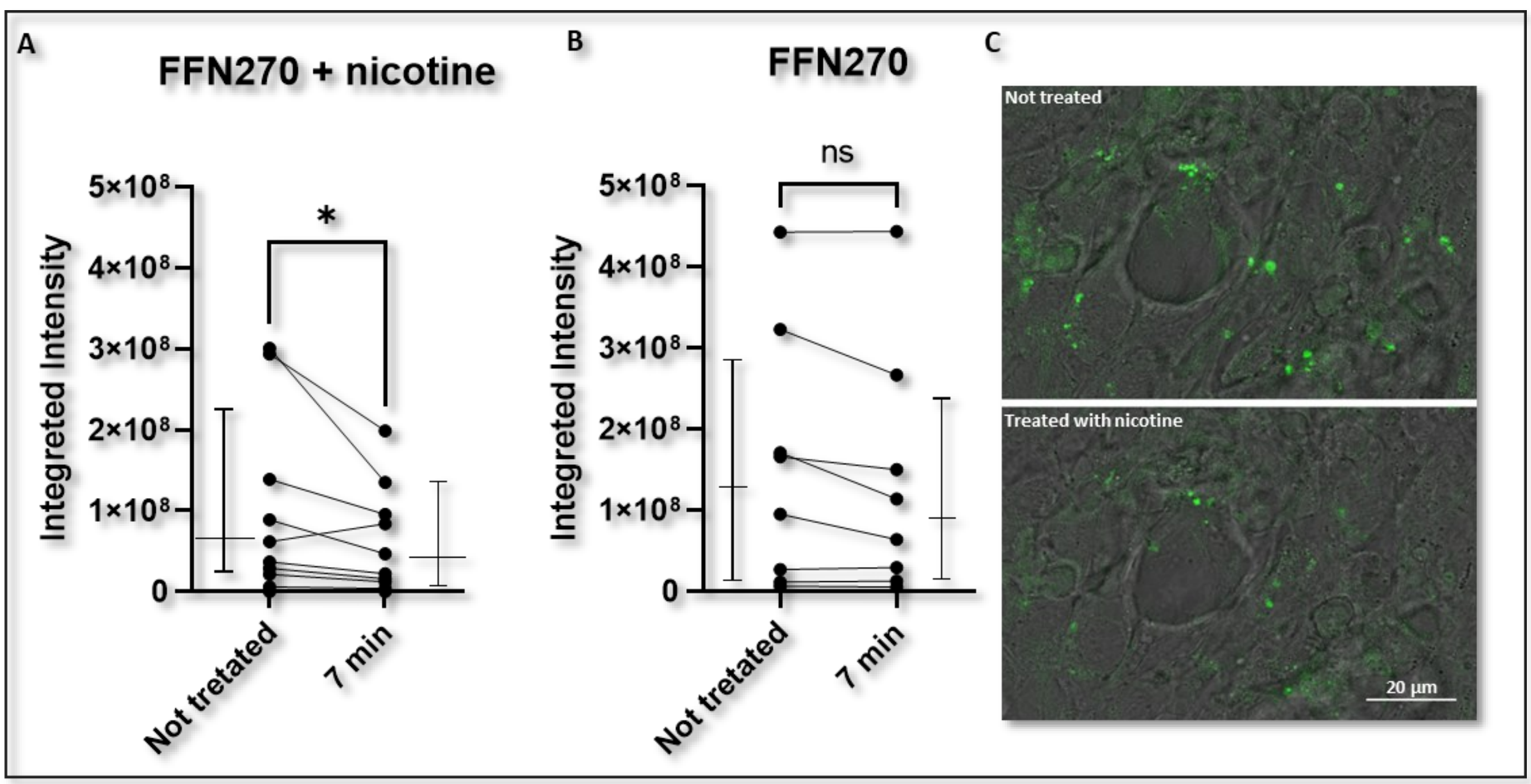

Figure S11: Integrated intensity analysis for the FFN270 assay

A: Co-cultures stained with 5 µM FNN270 and subsequently treated with 10 µM Nic. Images acquisition g was done prior to and 7 minutes following incubation (Median with IQR of 10 differentiations with 1 replicate). Test used: Paired t-test; p value: * = 0.0273.

B: Representation of control co-cultures stained with 5 µM FNN270, without Nic administration. Images acquisition was done prior to 7 minutes following incubation (Median with IQR of 8 differentiations with 1 replicate). Test used: Wilcoxon test, not significant

C: High-magnification images of the co-cultures stained with FFN270 before nicotine administration and after. Scale bar 20 µm.

Table S1: statistic for data not normally distributed.

Excel: “Table S1”

Table S2: statistic for data normally distributed.

Excel: “Table S2”

Table S3: Protocol for the differentiation of hiPSC-SN / CTR-SNs.

Day	Medium	Factors	Procedure
**Day 1**	KSR	LDN/SB	Medium change
**Day 2**	KSR	LDN/SB	Medium change
**Day 3**	KSR	LDN/SB/CH/DAPT /PD	Medium change
**Day 4**	KSR	LDN/SB/CH/DAPT/ PD/SHH/PMP	Medium change
**Day 5**	75% KSR/ 25% NNB	SB/CH/DAPT/PD/ SHH/PMP	Medium change
**Day 6**	75% KSR/ 25% NNB	CH/DAPT/PD/ SHH/PMP	Medium change
**Day 7**	50% KSR/ 50% NNB	CH/DAPT/PD/ SHH/PMP	Medium change
**Day 8**	50% KSR/ 50% NNB	CH/DAPT/PD/ SHH/PMP	Medium change
**Day 9**	25% KSR/ 75% NNB	SHH/PMP	Medium change
**Day 10**	25% KSR/ 75% NNB	SHH/PMP	Medium change
**Day 11**	NBB27	SHH/Pu/BMP4	Medium change
**Day 12**	NBB28	SHH/Pu/BMP4	**Replating**
**Day 13**	NBB29	SHH/Pu/BMP4	Medium change
**Day 14**	NBB30	BMP4	Medium change
**Day 15**	NBB31	BMP4	Medium change
**from Day 16**	NBB32	cAMP/AA/NGF/BDNF/ GDNF	Medium change

Table S4: List of factors used for the hiPSC-SN / CTR-SNs differentiation.

Factors	Complete Name	Brand	Final concentration
LDN	LDN-193189	Stemgent	500nM
SB	SB431542	Tocris	10uM
CH	CHIR99021	Stemgent	3uM
DAPT	DAPT	Tocris	10uM
PD	PD173074	Sigma Aldrich	0,2uM
SHH	Human Sonic Hedgehog	R&D System	100ng/mL
PMP	Purmorphamine	Stemgent	1uM
BMP4	Human Bone Morphogenetic Protein 4	R&D System	10ng/mL
cAMP	dibutyryl-cyclic-A	Enzo LifesciencesMP	0,2mM
AA	ascorbic acid	Sigma Aldrich	0,2mM
NGF	Human β-NGF	Peprotech	10ng/mL
BDNF	Human BDNF	Peprotech	20ng/mL
GDNF	Human GDNF	Peprotech	10ng/mL

Table S5: List of antibodies used in this work.

Excel: “Table S5”

Table S6:

TaqMan probes used in this work.

Gene	Fluorophore	Assay ID
**TH**	FAM	Hs00165941_m1
**DBH**	FAM	Hs01089840_m1
**RPL13**	FAM	Hs00366152_m1
**GAPDH**	FAM	Hs02786624_g1
**TNNI3**	FAM	Hs00165957_m1
**MYH7**	FAM	Hs01110632_m1
**β1AR**	FAM	Hs02330048_s1
**SEMA3C**	FAM	Hs00989373_m1
**NGF**	FAM	Hs00171458_m1
**GDNF**	FAM	Hs01931883_s1
**BDNF**	FAM	Hs02718934_s1
**HCN4**	FAM	Hs00975492_m1
**SCN5A**	FAM	Hs00165693_m1
**KCNA5**	FAM	Hs00969279_s1

Video:

Video S1: Timelapse video of a co-culture at 7 days. Magnification 10x.
